# Pore-scale modelling of methane-driven helium extraction in deep tight sandstones

**DOI:** 10.1038/s41598-026-60903-1

**Published:** 2026-08-25

**Authors:** Hao Zhao, Bowen Zhang, Jingong Zhang, Linyu Liu, Yiqing Yang, Yanan Zhou, Rui Kang

**Affiliations:** 1 Department of Geology, Northwest University, No. 229 Taibaibei Road, Xi’an, Shaanxi 710069 China; 2 AECC Xi’an Engine Control Technology Co., Ltd., No. 750 Daqing Road, Lianhu District, Xi’an, 710077 Shaanxi China; 3 Research Institute of Petroleum Exploration & Development, No. 20 Xueyuan Road, Haidian District, Beijing, 100083 China; 4 Research Institute of Exploration and Development, PetroChina Changqing Oilfield Company, Xi’an, Shaanxi 710018 China

**Keywords:** Helium, Tight sandstone, Methane, Pore-scale transport, Lattice Boltzmann, Energy science and technology, Solid Earth sciences

## Abstract

Helium is a strategic resource for cryogenic, aerospace, semiconductor, and other advanced technologies, but in deep tight sandstone reservoirs it may occur partly as dissolved helium in formation water, and its transfer into a producible gas phase remains poorly quantified. Here we examine methane-driven helium extraction under representative deep tight-reservoir conditions by coupling sub-voxel pore reconstruction, gas–water partitioning calculations, and pore-scale transport simulations. The analysis uses geological boundary constraints from the Ordos Basin, weakly supervised convolutional neural-network reconstruction of sub-voxel porosity, thermodynamic gas–water partitioning calculations, and lattice Boltzmann pore-scale transport simulations. The reconstructed digital rock better preserves micro–nano pore connectivity than simple binary segmentation and is used to estimate Knudsen/slip-controlled transport contrasts between helium and methane. The simulations indicate that helium can maintain a slightly higher apparent permeability than methane in nano-confined pore throats because of its smaller molecular diameter. This relative mobility advantage increases at low pressure but becomes small under the representative deep-reservoir pressure used in this study. Thermodynamic calculations indicate that high temperature, high pressure, and high salinity favour helium partitioning from the aqueous phase into the gas phase, whereas methane flow increases interfacial contact and convective stripping. Multi-slice extraction simulations further suggest that helium release is concentrated near gas–water contact zones and is later limited by diffusion-driven replenishment from adjacent water-bearing pore regions. The results support an analogue pore-scale interpretation of methane-assisted helium mobilization in a tight-sandstone pore network, but their reservoir-scale generality remains limited by the use of an analogue digital-rock dataset and slice-based pore-scale simulations.

**Supplementary Information:**

The online version contains supplementary material available at 10.1038/s41598-026-60903-1.

## Introduction

Helium is a critical resource widely used in advanced technologies due to its unique physical properties, including chemical inertness and extremely low boiling point^1–3^. At present, economically viable helium is mainly produced from natural gas reservoirs, where it typically occurs as a trace component associated with hydrocarbons^1,2,4^. Understanding the mechanisms controlling helium mobilization and enrichment in such systems is therefore important for resource evaluation and exploration.

In deep tight sandstone reservoirs, helium is primarily generated by radioactive decay in uranium- and thorium-bearing rocks and subsequently migrates into overlying formations^5–7^. Owing to its small molecular size and low solubility, helium is commonly present in very low concentrations and is largely dissolved in formation water within micro–nano pore systems^2,7^. Although the co-occurrence of hydrocarbons and helium is well documented^8–14^, the transition of helium from a dissolved state to producible free gas remains poorly quantified. In particular, the dynamic processes governing methane-driven extraction of dissolved helium in complex pore networks are not well constrained.

Previous studies on helium accumulation in sedimentary basins have mainly emphasized source rocks, radiogenic helium generation, carrier systems, structural evolution, and preservation conditions at the basin or reservoir scale^2,4,7^. In parallel, pore-scale studies of multiphase flow in porous media have shown that gas–water interfacial area, pore connectivity, wetting-state distribution, and stagnant or isolated pore regions can influence displacement and mass transfer^15,16^. Laboratory approaches such as core flooding, X-ray microtomography, and microfluidic experiments, together with numerical approaches including direct numerical simulation, pore-network modelling, and lattice Boltzmann methods, have helped constrain pore-scale gas–water transport and interfacial mass transfer^15,17,18^. In unconventional and tight reservoirs, previous studies have also shown that gas transport in nano-confined pores can be affected by Knudsen diffusion, slip flow, and Klinkenberg-type apparent permeability enhancement^17,19,20^. Helium-specific pore-scale and rarefied-gas simulations have also shown that helium may exhibit transport behaviour different from heavier gases under confined or low-pressure conditions, although these studies rarely address methane-driven release of dissolved helium from formation water in tight sandstone pore networks.

However, these advances do not fully resolve the specific problem addressed here. Most helium-related geological studies focus on helium source, migration pathway, and preservation at larger scales, whereas the pore-scale conversion of dissolved helium in formation water into a mobile gas phase remains insufficiently constrained^2,4,7^. Existing pore-scale gas-transport studies commonly focus on CH$_4$, CO$_2$, N$_2$, or shale-gas systems, with less attention to the molecular-size-controlled transport contrast between He and CH$_4$ under deep tight-sandstone conditions^17–19^. Gas–water mass-transfer studies provide useful physical analogues, but few have coupled deep-reservoir gas–water partitioning, methane-driven stripping, sub-voxel pore reconstruction, and pore-scale LBM simulation to examine whether thermodynamically favourable helium release can occur within spatially heterogeneous tight pore networks^15,16^. We therefore use a mechanism-oriented pore-scale modelling workflow to evaluate methane-driven helium extraction from formation water under representative deep tight-reservoir conditions.

To address this pore-scale aspect of the problem, we combine pore-structure reconstruction, thermodynamic gas–water partitioning, and pore-scale flow simulation. A weakly supervised three-dimensional convolutional neural network is used to reconstruct sub-voxel pore structures from CT images and to better characterize micro–nano pore connectivity^21–25^. Multicomponent lattice Boltzmann simulations are then performed on the reconstructed digital cores to examine gas transport behaviour under deep reservoir conditions. Thermodynamic models are incorporated to describe gas–water partitioning and mass transfer under high temperature, high pressure, and high salinity.

Here, methane-driven helium extraction is treated as a mechanism-oriented pore-scale problem. The Ordos Basin data constrain representative reservoir temperature, pressure, and formation-water salinity, whereas the Wilcox Tight Gas Sandstone digital rock is used as an analogue pore geometry rather than as a direct sample from the target interval. We first reconstruct a continuous sub-voxel porosity field from grayscale CT data and derive pore-throat-scale transport parameters, including hydraulic radius, Knudsen number, and He–CH$_4$ apparent permeability contrast. We then couple gas–water partitioning calculations with pore-scale LBM simulations to examine how methane flow promotes dissolved-helium release and how diffusion-limited replenishment controls late-stage extraction. The results are therefore analogue-based simulations designed to test a pore-scale mechanism, not direct reservoir-scale predictions for the Ordos Basin.

## Data and methods

We integrate geological constraints, well-log interpretation, digital rock data, and pore-scale numerical simulation to investigate helium mobilization in deep tight sandstone reservoirs.

Geological and thermodynamic boundary conditions were constrained using reservoir-scale data from the Ordos Basin. The target interval was selected from the deep tight-sandstone section of Well Ninggu 3, and the corresponding well-log profile was moved to the Supplementary Information because it provides geological context rather than pore-scale modelling results (Supplementary Fig. S1). A characteristic burial depth of approximately 3600 m was adopted. Regional burial-depth relationships for the Upper Paleozoic reservoirs were then used to define the representative temperature and pressure conditions of approximately 113$^\circ$C and 32.5 MPa, respectively (Supplementary Fig. S2). These values were used as boundary conditions for the gas–water partitioning calculations and pore-scale transport simulations.

Formation water chemistry is characterized using reported geochemical data from multiple wells in the Sulige gas field. The formation water is dominated by high-salinity CaCl$_2$-type brine, with total dissolved solids ranging from 11.35 to 46.45 g/L (average ~27 g/L) and pH values between 6.0 and 6.9. The detailed ionic composition and water type classification are summarized in Table 5, providing constraints for gas solubility and phase partitioning under reservoir conditions.

**Fig. 1:**
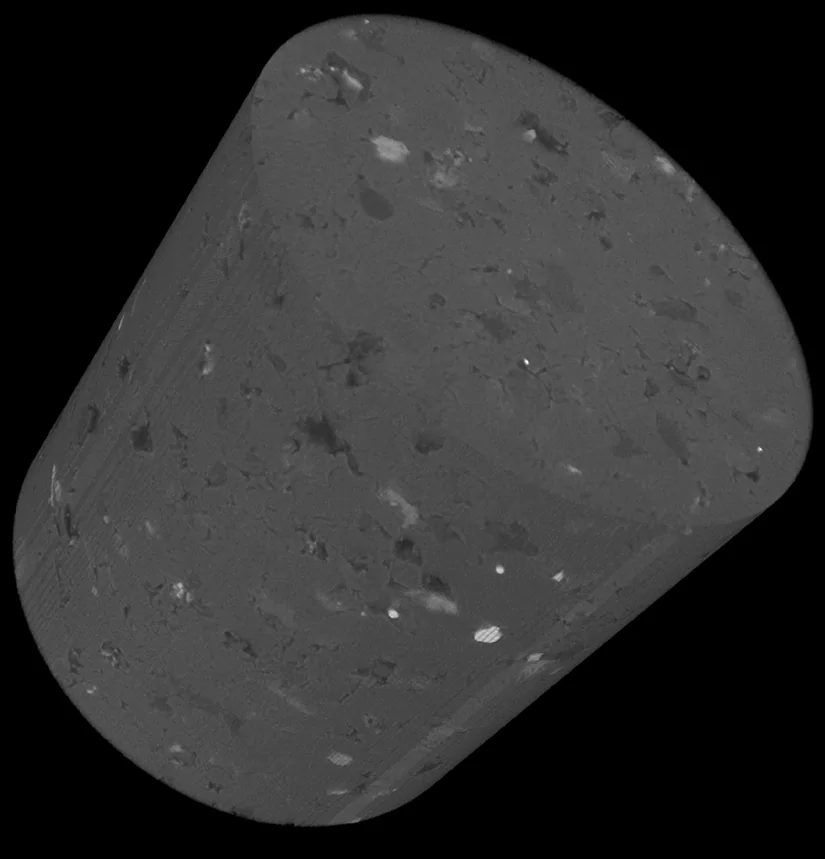
Three-dimensional CT image of the Wilcox Tight Gas Sandstone sample from the Digital Porous Media Portal dataset^26^.

Pore structure data were derived from the Wilcox Tight Gas Sandstone digital rock model^26^, following digital-rock characterization workflows commonly used for tight sandstone pore-system analysis^23–25,27^. The corresponding three-dimensional CT image of the rock sample is shown in Fig. 1, based on the Wilcox Tight Gas Sandstone dataset and consistent with tight-sandstone digital-rock workflows using X-ray microtomography^24,26^. The dataset consists of an 8-bit grayscale volume with dimensions of 915 *×* 924 *×* 910 voxels and a spatial resolution of 2.7 *μ* m. The sample has a porosity of 8.9% and a permeability of 0.1147 mD. It is therefore used here as an analogue low-porosity and low-permeability tight-sandstone pore geometry, rather than as a direct representative sample of the Ninggu 3 reservoir. To improve the characterization of unresolved pore features, a weakly supervised three-dimensional convolutional neural network was applied to reconstruct a continuous sub-voxel porosity field from the grayscale CT data.

### Digital-rock analogue and transferability

The pore-structure model used in this study was derived from the Wilcox Tight Gas Sandstone digital-rock dataset rather than from a core sample of the Ninggu 3 target interval. This choice was made because a high-resolution three-dimensional micro-CT dataset from the target Ordos interval was not available for reproducible pore-scale simulation. The Wilcox dataset was therefore used as an analogue tight-sandstone pore geometry to test the pore-scale mechanism of methane-driven helium mobilization.

This analogue use is supported by the tight-reservoir character of the Wilcox digital rock. The dataset has a measured porosity of 8.9% and a permeability of 0.1147 mD, which fall within the low-porosity and low-permeability range typical of tight sandstone reservoirs. Its micro-CT resolution of 2.7 *μ* m provides a basis for reconstructing sub-voxel pore connectivity and evaluating nano-confined transport effects after applying the weakly supervised reconstruction workflow. In this study, the Ordos Basin data define geological and thermodynamic boundary conditions, including burial depth, temperature, pressure, and formation-water salinity, whereas the Wilcox digital rock provides the pore-network geometry for mechanism testing.

Therefore, the simulations should not be interpreted as direct quantitative predictions for the Ninggu 3 reservoir. Instead, they represent a mechanism-oriented analogue experiment designed to examine whether methane flow can promote dissolved-helium transfer from formation water into a gas phase within a tight-sandstone pore network, and how this process is affected by pore-throat confinement, He–CH$_4$ relative mobility contrast, and diffusion-limited replenishment.

Based on the reconstructed pore space, gas transport was simulated using a multicomponent lattice Boltzmann formulation^18,28–32^. Helium and methane were treated as the main components, and non-continuum effects were incorporated through effective transport parameters accounting for Knudsen diffusion and slippage. Thermodynamic equilibrium between gas and aqueous phases was described using temperature-dependent solubility relationships corrected for pressure and salinity^33–39^. Methane injection was imposed as a continuous driving condition to simulate advective flow, interfacial mass transfer, and helium release from formation water. The simulations tracked helium concentration, mass balance, and spatial redistribution during the extraction process.

To improve transparency and reproducibility, the equations used in this study are grouped into four categories: literature-based equations, modified equations adapted from established models, algebraically derived relationships, and implementation-specific numerical definitions. Standard literature-based equations include the lattice Boltzmann evolution equation, Knudsen-number definition, Klinkenberg-type apparent-permeability relationship, Peng–Robinson equation of state, Henry-law-type solubility, Poynting pressure correction, and Setschenow salinity correction. Modified equations include the Kozeny–Carman-type permeability estimate using an effective hydraulic throat radius and cutoff-based effective porosity. Derived relationships include the inverse grayscale mapping and the He–CH$_4$ mobility inequalities. Implementation-specific definitions include the grayscale weak-label mapping, sliding-window averaging, and local extraction coefficient used in the parameterized LBM extraction model. The provenance of each equation group and the corresponding references are summarized in Supplementary Table S2.

Table 1 summarizes the principal symbols used in the pore-scale calculations. To avoid ambiguity among parameters used in different sub-models, each symbol is used uniquely throughout the manuscript. In particular, $\beta _t$ is reserved for the throat-constriction factor, whereas the velocity-enhancement coefficient in the extraction model is denoted by $\chi _u$.

**Table 1:** Key symbols used in the pore-scale modelling workflow.

Symbol	Definition	Unit/normalization	Model module
$\hat{\phi }(\textbf{x})$	Reconstructed effective sub-voxel porosity	dimensionless	CNN reconstruction
$\phi _{\textrm{cut}}(\textbf{x})$	Cutoff-based effective porosity used for permeability estimation	dimensionless	Hydraulic-radius model
$\phi _c$	Porosity cutoff threshold	dimensionless	Hydraulic-radius model
$\phi _w(\textbf{x})$	Grayscale-derived weak-label porosity	dimensionless	CNN weak-label generation
$\phi _{\textrm{bound}}$	Bound-water pore fraction used in the extraction model	dimensionless	LBM extraction model
$S_{wr}$	Residual water saturation assigned to gas-accessible pores	dimensionless	Fluid distribution
$S_{v,\textrm{reg}}(\textbf{x})$	Regularized specific surface area	m$^{-1}$	Geometry mapping
$r_{\textrm{geo}}(\textbf{x})$	Geometric pore radius	m	Geometry mapping
$r_{\textrm{hyd}}(\textbf{x})$	Effective hydraulic throat radius	m	Hydraulic-radius model
$r_{\min },\,r_{\max }$	Lower and upper bounds of hydraulic radius	m	Hydraulic-radius truncation
$\beta _t$	Throat-constriction factor	dimensionless	Hydraulic-radius model
$k_{\textrm{int}}(\textbf{x})$	Intrinsic permeability field	m$^2$ or mD	Permeability model
$k_{\textrm{app}}(\textbf{x})$	Apparent permeability field	m$^2$ or mD	Slip-flow correction
$\textrm{Kn}(\textbf{x})$	Local Knudsen number	dimensionless	Slip-flow analysis
$\omega _{\textrm{bb}}(\textbf{x})$	Porosity-dependent bounce-back weight	lattice unit	LBM model
$\Psi _{\textrm{sc}}(T,P,S)$	Temperature-, pressure-, and salinity-dependent Shan–Chen interaction parameter	lattice unit/normalized	Shan–Chen LBM
$R_{\textrm{diff}}(\textbf{x})$	Porosity-weighted diffusion-length proxy	normalized	Helium scalar transport
$C_{\textrm{Kn}}(\textbf{x})$	Knudsen hindrance factor for helium diffusion	dimensionless	Helium scalar transport
*m*, *n*	Phase indices for water and methane	dimensionless	Shan–Chen LBM
$\chi _u$	Velocity-enhancement coefficient for local helium removal	lattice-time normalized	LBM extraction model
$k_{\textrm{loc}}(\textbf{x},t)$	Dimensionless per-step helium removal fraction	dimensionless per time step	LBM extraction model
$K_i^{\textrm{g}/\textrm{w}}$	Gas-to-water partition coefficient of component *i*	dimensionless	Gas solubility model

**Table 2:** Key parameters supporting the use of the Wilcox digital rock as a tight-sandstone analogue.

Parameter	Ordos tight sandstone	Wilcox digital rock	Role in this study
Geological use	Ninggu 3/Sulige constraint	Analogue pore geometry	Boundary vs. pore model
Depth	~3600 m	–	Ordos constraint
Temperature	~113 $^\circ$ C	–	Ordos constraint
Pressure	~32.5 MPa	–	Ordos constraint
Formation water	CaCl$_2$ type; TDS 11.35–46.45 g/L; avg. ~27 g/L; pH 6.0–6.9	–	Ordos constraint
Lithology	Tight sandstone	Tight gas sandstone	Analogue basis
Porosity	1.36–9.55%; ~8.8%	8.9%	Comparable low porosity
Permeability	0.004–1.3625 mD; ~0.39 mD	0.1147 mD	Comparable low permeability
Pore-throat scale	0.006–10 *μ* m; locally nano–micro scale	voxel size 2.7 *μ* m; sub-voxel reconstruction	Pore-scale transport
Main pore types	Intergranular pores; dissolution pores; micropores; microfractures	Cemented sandstone; feldspar dissolution; microporosity	Tight pore network
Interpretation	Target reservoir setting	Analogue digital rock	Mechanistic simulation only

As summarized in Table 2, the Wilcox digital rock is not treated as a direct sample from the Ninggu 3 interval. It is used as an analogue tight-sandstone pore geometry because its porosity and permeability fall within the low-porosity and low-permeability range reported for Ordos tight sandstones. In this study, the Ordos data constrain the burial depth, temperature, pressure, and formation-water chemistry, whereas the Wilcox dataset provides the pore-network geometry for testing methane-driven helium mobilization. Therefore, the results are interpreted as mechanism-oriented pore-scale simulations rather than direct reservoir-specific predictions.

All equations used in the modelling workflow were classified according to their provenance as literature-based, modified from established models, algebraically derived in this study, or implementation-specific numerical definitions. A summary of equation provenance and supporting references is provided in Supplementary Table S2. Standard models are cited at their first use in the main text.

## Sub-voxel pore structure reconstruction

Tight sandstones are characterized by complex pore systems dominated by micro–nano-scale pores and throats that are often below the resolution of conventional micro-CT imaging. As a result, binary segmentation methods fail to capture sub-resolution pore structures and may underestimate pore connectivity^40–43^. To reduce this limitation, a sub-voxel reconstruction approach was developed to generate a continuous porosity field from grayscale CT data, providing a physically consistent representation of pore geometry for subsequent transport simulations.

### Grayscale–porosity mapping

The grayscale–porosity mapping used here is an implementation-specific weak-label construction based on a partial-volume interpretation of CT grayscale intensity, rather than a standard mineral-resolved porosity inversion. In multi-mineral tight sandstones, grayscale intensity may also be affected by X-ray attenuation contrasts among quartz, feldspar, clay minerals, and cement. Therefore, Eqs. (1)–(4) are used to generate physically guided weak labels for CNN training, not to provide an independently calibrated mineral-specific porosity map. The clipping and smoothing operations are numerical steps designed to reduce the influence of isolated noise and to preserve spatial continuity in the weak-label field.

**Fig. 2:**
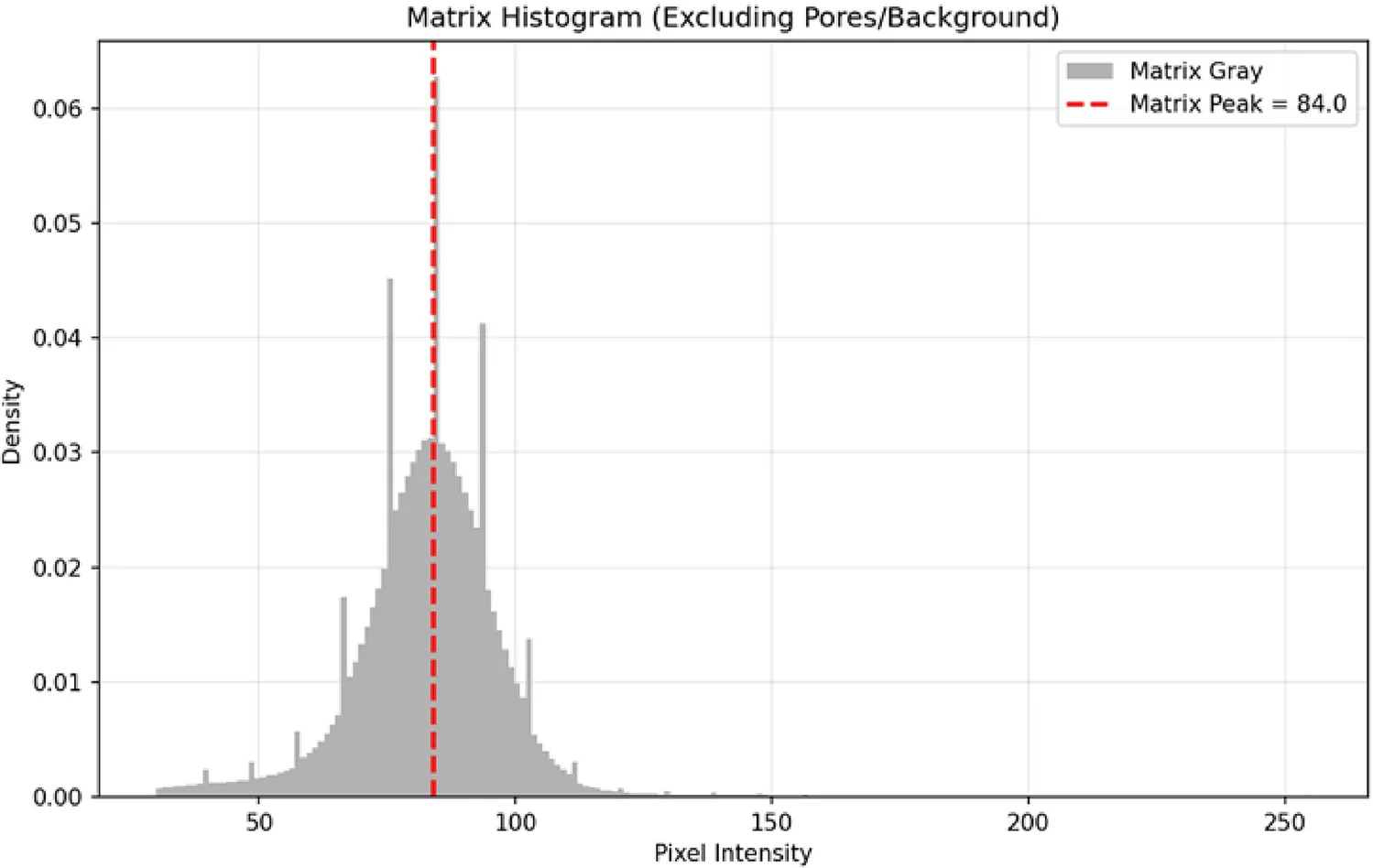
Global grayscale histogram of the CT image used to calibrate the pore and solid reference intensities.

First, the raw grayscale field $I(\textbf{x})$ was clipped to the physically meaningful range defined by the pore and solid reference intensities:

1 $$\begin{aligned} I_c(\textbf{x})=\min \!\bigl (\max \!\left( I(\textbf{x}), I_{\textrm{pore}}\right) , I_{\textrm{solid}}\bigr ) \end{aligned}$$

where $I_{\textrm{solid}}$ and $I_{\textrm{pore}}$ represent the reference grayscale values of solid matrix and pore space, respectively. In this study, these values were calibrated from the global grayscale histogram (Fig. 2) and set to:

2 $$\begin{aligned} I_{\textrm{solid}}=84, \qquad I_{\textrm{pore}}=15 \end{aligned}$$

Based on the clipped grayscale field, the weak-label porosity field $\phi _w(\textbf{x})$ was obtained through a linear transformation:

3 $$\begin{aligned} \phi _w(\textbf{x})=1-\frac{I_c(\textbf{x})-I_{\textrm{pore}}}{I_{\textrm{solid}}-I_{\textrm{pore}}+\varepsilon } \end{aligned}$$

where *\varepsilon* is a small constant to avoid numerical instability.

To suppress imaging noise and enforce spatial continuity, a three-dimensional averaging filter was applied:

4 $$\begin{aligned} \tilde{\phi }_w(\textbf{x})=\frac{1}{|\Omega _{\textbf{x}} |}\sum _{i\in \Omega _{\textbf{x}}} \phi _w(i), \qquad \Omega _{\textbf{x}} \in 3\times 3\times 3 \end{aligned}$$

This “soft label” formulation reduces non-physical fluctuations while preserving gradual transitions at pore–matrix interfaces, providing stable supervision for neural network training.

### Weakly supervised 3D CNN reconstruction

A weakly supervised three-dimensional convolutional neural network based on a lightweight 3D U-Net architecture was employed to reconstruct the sub-voxel porosity field^21,22,44–48^. The network maps the normalized CT image $I(\textbf{x})$ to a continuous porosity prediction $\hat{\phi }(\textbf{x})$, constrained within the range [0, 1] using a sigmoid activation function.

**Fig. 3:**
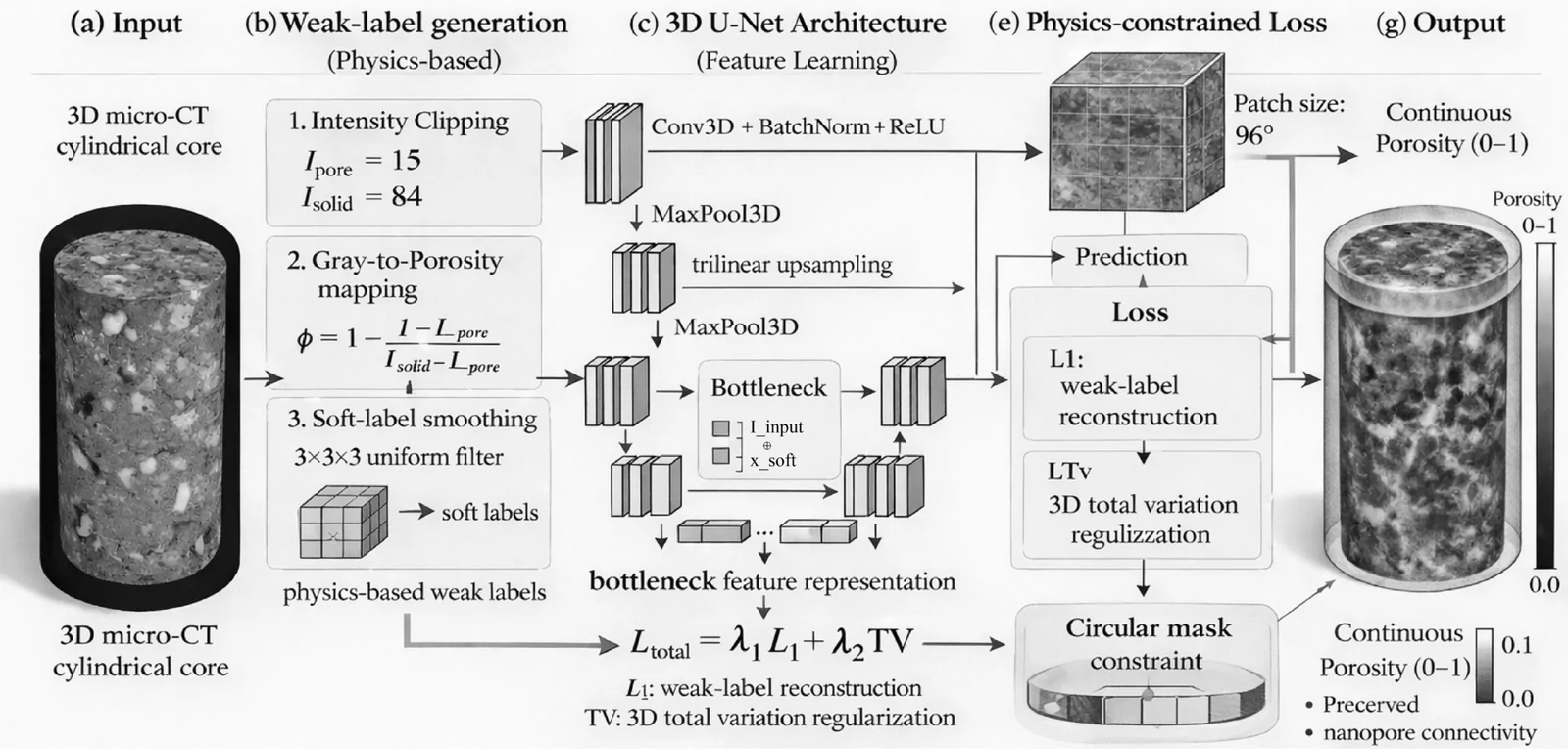
Workflow of the physics-constrained weakly supervised 3D U-Net framework for sub-voxel porosity reconstruction in tight sandstone. (**a**) Input 3D micro-CT grayscale cylindrical core. (**b**) Generation of physics-based weak labels, including intensity clipping using calibrated thresholds ($I_{\textrm{pore}}=15$, $I_{\textrm{solid}}=84$), grayscale-to-porosity mapping, and $3\times 3\times 3$ uniform filtering to obtain continuous soft labels. (**c**) Lightweight 3D U-Net architecture for feature learning, consisting of convolution, batch normalization, ReLU activation, max pooling, and trilinear upsampling with skip connections. (**d**) Joint loss function combining L1 weak-label reconstruction loss and three-dimensional total variation (TV) regularization. (**e**) Sliding-window inference using overlapping patches with a patch size of $96^3$ voxels, an overlap of 16 voxels, and a stride of 80 voxels. (**f**) Cylindrical mask applied to remove boundary artefacts and non-core background regions. (**g**) Output continuous sub-voxel porosity field (0–1), representing pore connectivity and spatial heterogeneity in tight sandstone.

The model consists of three encoding–decoding levels with skip connections, enabling simultaneous extraction of global context and local spatial features. This encoder–decoder strategy is also consistent with recent digital-rock studies that use volumetric CNNs to connect pore morphology with petrophysical properties and flow response^49,50^. The network architecture follows the encoder–decoder concept of U-Net^47^ and its three-dimensional extension for volumetric segmentation^48^. The loss function combines an L1 weak-label reconstruction term with total-variation regularization. The total-variation term follows the general principle of TV-based image regularization originally introduced for edge-preserving image denoising^51^, and is used here to suppress high-frequency fluctuations in the predicted effective porosity field. The AdamW optimizer and cosine learning-rate scheduling follow established deep-learning optimization strategies^52,53^. The reconstruction workflow is illustrated in Fig. 3.

To ensure both fidelity to the weak labels and spatial smoothness, a joint loss function was defined as:

5 $$\begin{aligned} L=\lambda _{\textrm{weak}}L_{\textrm{L1}}+\lambda _{\textrm{TV}}L_{\textrm{TV}} \end{aligned}$$

where the L1 reconstruction loss is:

6 $$\begin{aligned} L_{\textrm{L1}}=\frac{1}{N}\sum _{\textbf{x}} \left| \hat{\phi }(\textbf{x})-\tilde{\phi }_w(\textbf{x})\right| \end{aligned}$$

and the total variation regularization term is defined as:

7 $$\begin{aligned} L_{\textrm{TV}}=\frac{1}{N}\sum _{\textbf{x}} \left[ (\nabla _x\hat{\phi }(\textbf{x}))^2+(\nabla _y\hat{\phi }(\textbf{x}))^2+(\nabla _z\hat{\phi }(\textbf{x}))^2\right] \end{aligned}$$

where *N* is the number of voxels. In this study, the weighting coefficients were set to:

8 $$\begin{aligned} \lambda _{\textrm{weak}}=5.0, \qquad \lambda _{\textrm{TV}}=0.01 \end{aligned}$$

The network was trained using the AdamW optimizer with an initial learning rate of $5\times 10^{-4}$ and cosine annealing scheduling. Training was performed on randomly sampled patches of size $96^3$, enabling efficient learning of local pore structures.

For full-volume reconstruction, a sliding-window inference strategy with overlap was applied. The final prediction was obtained by averaging overlapping patches:

9 $$\begin{aligned} \hat{\phi }(\textbf{x})=\frac{\sum _{k=1}^{M} w_k(\textbf{x})\,\hat{\phi }_k(\textbf{x})}{\sum _{k=1}^{M} w_k(\textbf{x})} \end{aligned}$$

where $w_k(\textbf{x})$ indicates whether voxel *x* is covered by the *k*-th patch.

To remove boundary artefacts and non-core regions, a cylindrical mask was applied during post-processing, with a radius defined as 98% of the shorter lateral dimension of the sample. The resulting effective sub-voxel porosity field was used as a continuous pore-geometry input for the subsequent pore-scale transport simulations.

### Model training and porosity field reconstruction

The proposed 3D U-Net model was trained using an AdamW optimizer with weight decay to improve generalization. The initial learning rate was set to $5\times 10^{-4}$, and a cosine annealing learning-rate scheduler was employed to ensure stable convergence:

10 $$\begin{aligned} \eta _t=\eta _{\min }+\frac{1}{2}(\eta _0-\eta _{\min })\left( 1+\cos \frac{t\pi }{T}\right) \end{aligned}$$

where $\eta _0$ is the initial learning rate, $\eta _{\min }$ is the minimum learning rate, *t* is the current epoch, and *T* is the total number of epochs.

Due to the large size of the 3D CT volume, a patch-based training strategy was adopted. The input data were randomly sampled into cubic patches of size $96^3$, enabling efficient training while preserving local spatial features. Automatic mixed precision (AMP) was used to accelerate training and reduce memory consumption.

To evaluate the reconstruction performance, the predicted porosity field was compared with the measured bulk porosity ($\phi _{\textrm{ref}}=8.9\%$). The mean porosity of the reconstructed field is defined as:

11 $$\begin{aligned} \bar{\phi }=\frac{1}{N}\sum _{\textbf{x}} \hat{\phi }(\textbf{x}) \end{aligned}$$

where *N* is the total number of voxels. The relative error is calculated as:

12 $$\begin{aligned} \varepsilon =\frac{\left| \bar{\phi }-\phi _{\textrm{ref}}\right| }{\phi _{\textrm{ref}}} \end{aligned}$$

The final reconstruction model used cubic patches of $96^3$ voxels. During full-volume sliding-window inference, an overlap of 16 voxels was used between adjacent patches, corresponding to a stride of 80 voxels. The configuration with 25 training epochs, $I_{\textrm{pore}}=15$, and a final loss below 0.1 yielded a reconstructed mean porosity close to the measured bulk porosity. This configuration was therefore selected for the final model.

**Fig. 4:**
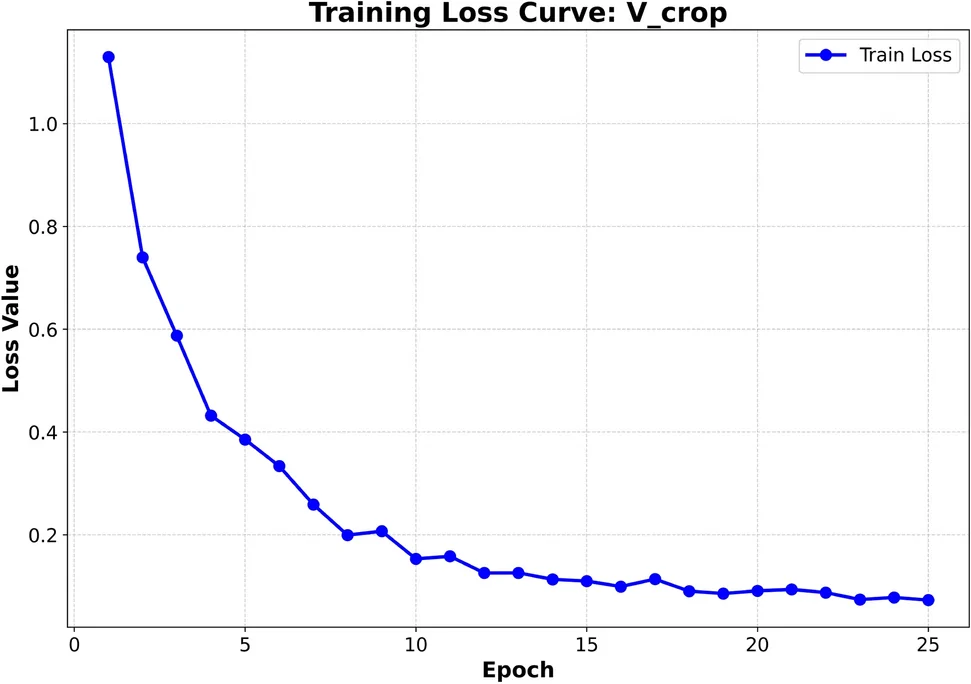
Training loss curve of the 3D U-Net model for sub-voxel porosity reconstruction. The model was trained for 25 epochs using the AdamW optimizer with an initial learning rate of $5\times 10^{-4}$ and cosine annealing scheduling. The total loss consists of L1 weak-label reconstruction loss and total variation (TV) regularization. The loss decreases rapidly during the initial stage and gradually converges without oscillation, indicating stable training behaviour.

The training loss evolution is shown in Fig. 4, where the loss decreases rapidly during the initial epochs and gradually converges under cosine annealing without noticeable oscillations, indicating stable training behaviour.

After training, full-volume reconstruction was performed using a sliding-window inference strategy with overlapping patches. Unless otherwise stated, all full-volume inference results reported in this study were generated using a patch size of $96^3$ voxels and an overlap of 16 voxels. The final porosity field was obtained by averaging overlapping predictions (9). A cylindrical mask was applied to remove boundary artefacts and background noise.

**Fig. 5:**
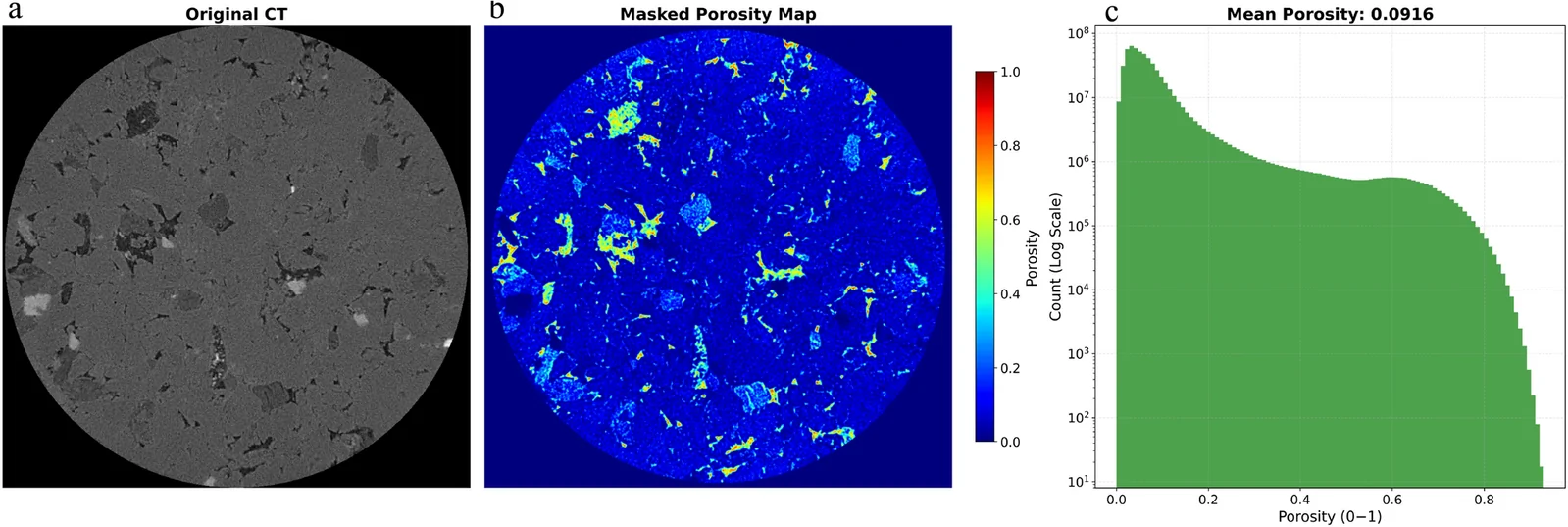
Comparison between original CT slices and reconstructed sub-voxel porosity field. (**a**) Original grayscale CT image. (**b**) Reconstructed continuous porosity field (0–1). (**c**) Porosity distribution histogram within the valid cylindrical region. The reconstructed field preserves large pore structures while enhancing micro–nano-scale connectivity. The mean porosity is approximately 9.16%, consistent with the measured bulk porosity (8.9%).

The reconstructed results are shown in Fig. 5, which compares the original CT slice and the predicted porosity field. The model successfully preserves large pore structures while revealing previously unresolved micro–nano-scale connectivity. The reconstructed mean porosity is approximately 9.16%, close to the measured bulk porosity of 8.9%. This agreement supports the use of the reconstructed field as an effective pore-geometry input for the subsequent pore-scale transport simulations (Table 3).

**Table 3:** Sensitivity analysis of training epochs, pore-reference intensity, and reconstruction loss for porosity field reconstruction. The final sliding-window inference used a fixed patch size of $96^3$ voxels and an overlap of 16 voxels.

Epoch	$I_{\textrm{pore}}$	Final loss value	Mean porosity	Reference porosity	Error
25	0	0.18	11.57	8.9	30%
25	0	0.05	12.84	8.9	44%
25	0	0.06	11.25	8.9	26%
25	15	0.05	9.29	8.9	4%
25	15	0.07	9.16	8.9	3%
25	15	0.054	9.24	8.9	4%
50	0	0.01	8.81	8.9	1%
50	0	0.009	8.32	8.9	7%
50	0	0.011	8.4	8.9	6%
50	15	0.008	6.69	8.9	25%
50	15	0.01	7.37	8.9	17%
50	15	0.009	7.44	8.9	16%

### Physical self-consistency check of sub-voxel reconstruction

Direct ground-truth measurement of sub-voxel porosity in tight sandstone is not available for the present dataset because of resolution limitations and the lack of an independently calibrated mineral-resolved porosity map. Therefore, the inverse grayscale comparison was used as a physical self-consistency check rather than as full independent validation. This check evaluates whether the reconstructed effective porosity field can reproduce the main grayscale texture and intensity distribution of the original CT image after inverse mapping. It does not prove that each voxel-scale porosity value is unique or mineralogically exact. The inverse grayscale relationship in Eq. (13) is algebraically derived from the grayscale–porosity mapping in Eq. (3) and is used only for internal self-consistency checking. The image-comparison metrics in Eqs. (14)–(17), including MAE, RMSE, PSNR, and SSIM, are standard image-quality and error metrics. In particular, SSIM follows the structural similarity index proposed by Wang et al.^54^. These metrics support the internal consistency of the reconstruction but do not constitute independent voxel-scale validation of a mineral-resolved porosity field.

#### Inverse grayscale reconstruction

Based on the grayscale–porosity mapping defined in Section "Grayscale–porosity mapping", the reconstructed grayscale field $\hat{I}(\textbf{x})$ can be obtained from the predicted porosity field:

13 $$\begin{aligned} \hat{I}(\textbf{x})=I_{\textrm{solid}}-\hat{\phi }(\textbf{x})\left( I_{\textrm{solid}}-I_{\textrm{pore}}\right) \end{aligned}$$

This inverse mapping allows direct comparison between reconstructed and original CT images.

#### Quantitative error metrics

To quantify grayscale-domain self-consistency, four standard metrics were used:

(1) Mean absolute error (MAE)

14 $$\begin{aligned} \textrm{MAE}=\frac{1}{N}\sum _{\textbf{x}} \left| I(\textbf{x})-\hat{I}(\textbf{x})\right| \end{aligned}$$

(2) Root mean square error (RMSE)

15 $$\begin{aligned} \textrm{RMSE}=\sqrt{\frac{1}{N}\sum _{\textbf{x}} \left( I(\textbf{x})-\hat{I}(\textbf{x})\right) ^2} \end{aligned}$$

(3) Peak signal-to-noise ratio (PSNR)

16 $$\begin{aligned} \textrm{PSNR}=10\log _{10}\left( \frac{I_{\max }^2}{\textrm{RMSE}^2}\right) \end{aligned}$$

(4) Structural similarity index (SSIM)

17 $$\begin{aligned} \textrm{SSIM}=\frac{(2\mu _I\mu _{\hat{I}}+C_1)(2\sigma _{I\hat{I}}+C_2)}{(\mu _I^2+\mu _{\hat{I}}^2+C_1)(\sigma _I^2+\sigma _{\hat{I}}^2+C_2)} \end{aligned}$$

where *μ*, *σ*, and $\sigma _{I\hat{I}}$ denote mean, variance, and covariance, respectively.

#### Self-consistency check results

**Fig. 6:**
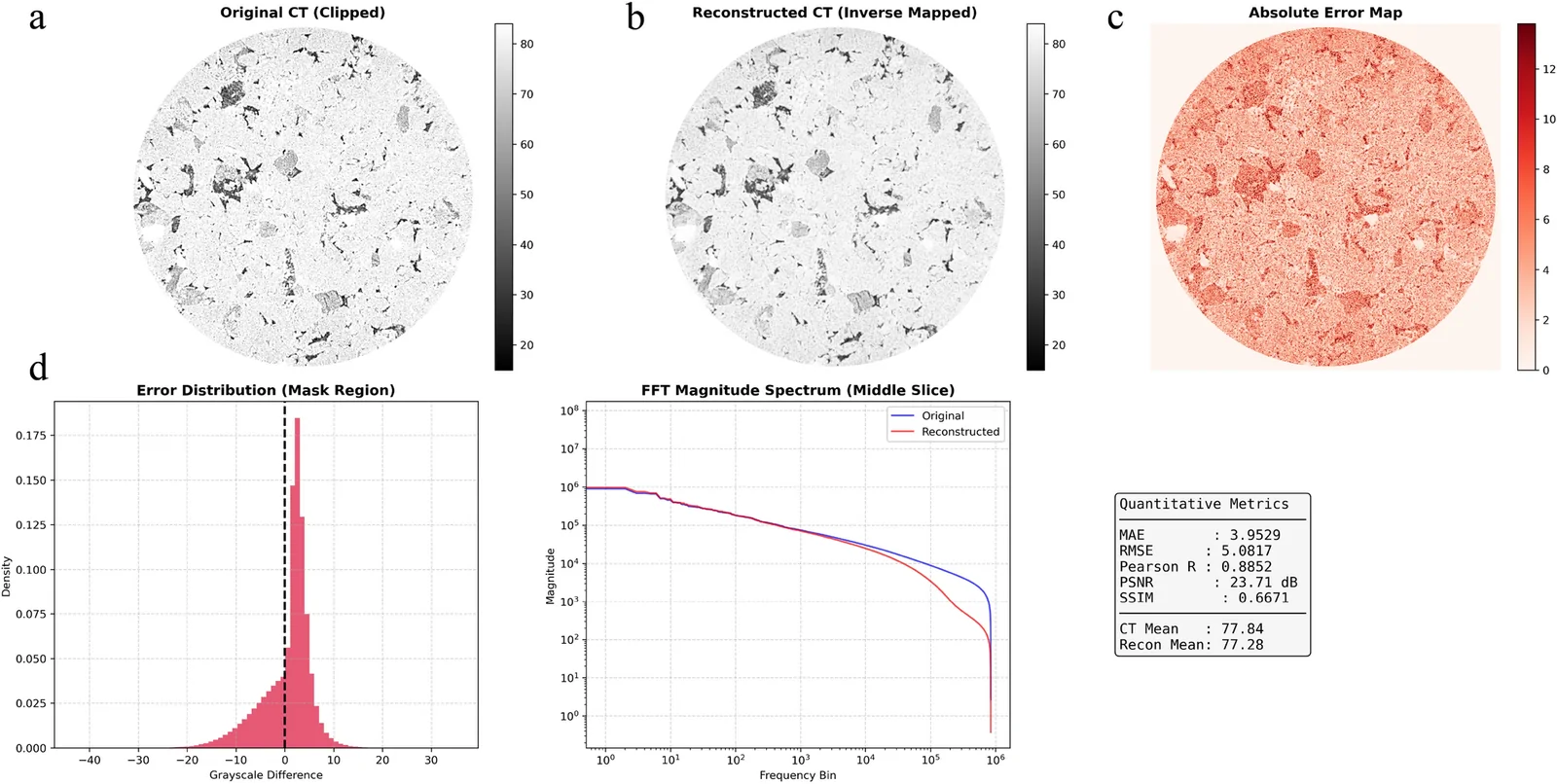
Physical self-consistency check of the reconstructed effective porosity field through inverse grayscale mapping. (**a**) Original CT image. (**b**) Reconstructed grayscale field obtained from the predicted effective porosity field. (**c**) Difference map between original and reconstructed images. (**d**) Grayscale-value comparison and statistical distribution. The original CT mean grayscale value is 77.84, and the reconstructed mean grayscale value is 77.28. Quantitative metrics include MAE = 3.9529, RMSE = 5.0817, Pearson *R = 0.8852*, PSNR = 23.71 dB, and SSIM = 0.6671. These metrics provide an internal consistency check rather than independent voxel-scale validation.

The comparison between the reconstructed and original CT images is shown in Fig. 6. Visually, the reconstructed grayscale field reproduces the main matrix texture and high-porosity regions of the original CT image, while suppressing part of the high-frequency imaging noise. Quantitatively, the mean grayscale value of the original CT image is 77.84, whereas the reconstructed grayscale field has a mean value of 77.28. The MAE and RMSE are 3.9529 and 5.0817, respectively, and the Pearson correlation coefficient is 0.8852. The PSNR is 23.71 dB, and the SSIM is 0.6671. These metrics support the internal consistency of the grayscale-based reconstruction and indicate that the main grayscale structure is retained after inverse mapping.

However, this comparison should not be interpreted as independent validation of a mineral-resolved sub-voxel porosity field. Because the inverse grayscale image is calculated using the same grayscale–porosity relationship used to generate the weak labels, the test mainly checks whether the reconstruction is consistent with the adopted mapping and with the global CT intensity structure. The reconstructed porosity field is therefore used as an effective pore-geometry input for subsequent pore-scale transport simulations, and the uncertainty associated with mineral attenuation contrasts is considered in the Discussion.

### Benchmark-supported plausibility assessment of the reconstructed porosity field

The inverse grayscale reconstruction was used only as a physical self-consistency check rather than as an independent validation of the true sub-voxel pore space. To provide additional benchmark support for the CNN-derived continuous porosity field, three complementary tests were performed: a porosity-matched threshold-segmentation baseline, hold-out slice self-consistency checks, and porosity–permeability plausibility assessment.

**Fig. 7:**
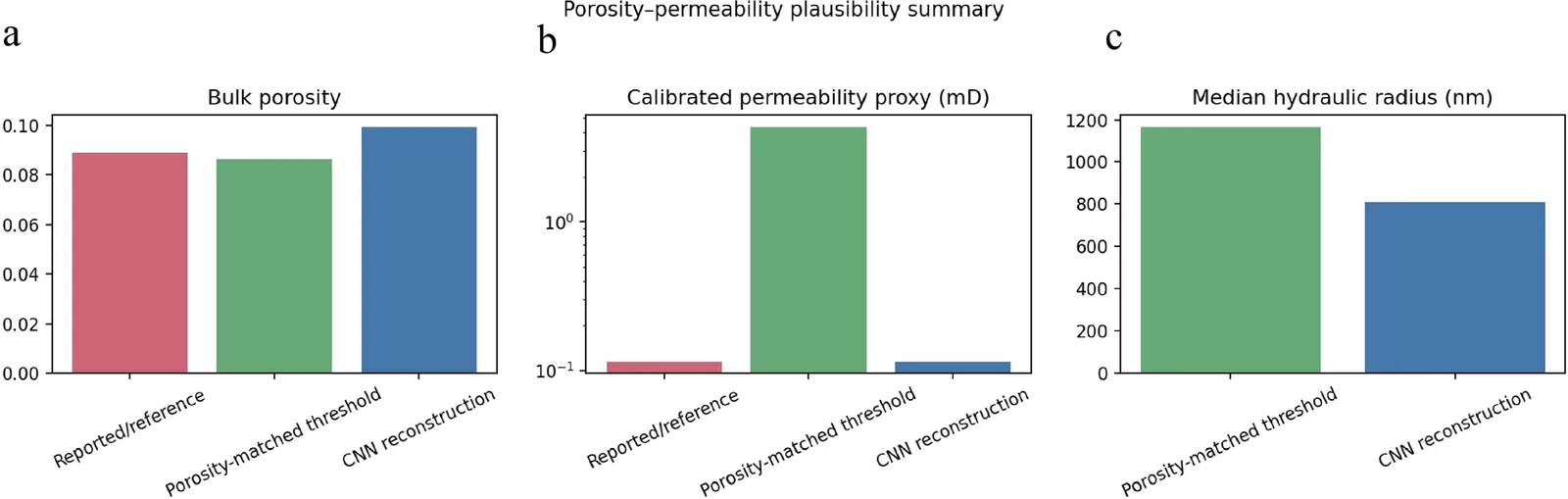
Benchmark comparison of the CNN-derived continuous porosity field with a porosity-matched threshold-segmentation reference. (**a**) Bulk porosity comparison among the reported Wilcox value, the porosity-matched threshold case, and the CNN-derived field. (**b**) Calibrated permeability-proxy comparison. (**c**) Median hydraulic-radius comparison. The comparison shows that similar bulk porosity can still produce different pore–throat connectivity and flow-capacity estimates. The threshold-segmentation case is therefore used only as a porosity-matched reference, not as ground-truth pore segmentation.

First, a conventional threshold-segmentation baseline was constructed for comparison. The threshold was selected to reproduce a bulk porosity close to the reported Wilcox tight sandstone porosity, and was therefore used as a porosity-matched reference case rather than as ground-truth pore segmentation. The CNN-derived bulk porosity was 0.099, close to the reported value of 0.089, whereas the porosity-matched threshold baseline yielded a similar bulk porosity but a much higher permeability proxy and a broader hydraulic-radius distribution. This indicates that binary thresholding can reproduce bulk porosity after tuning, but may distort pore–throat connectivity and flow-capacity estimates (Fig. 7).

**Fig. 8:**
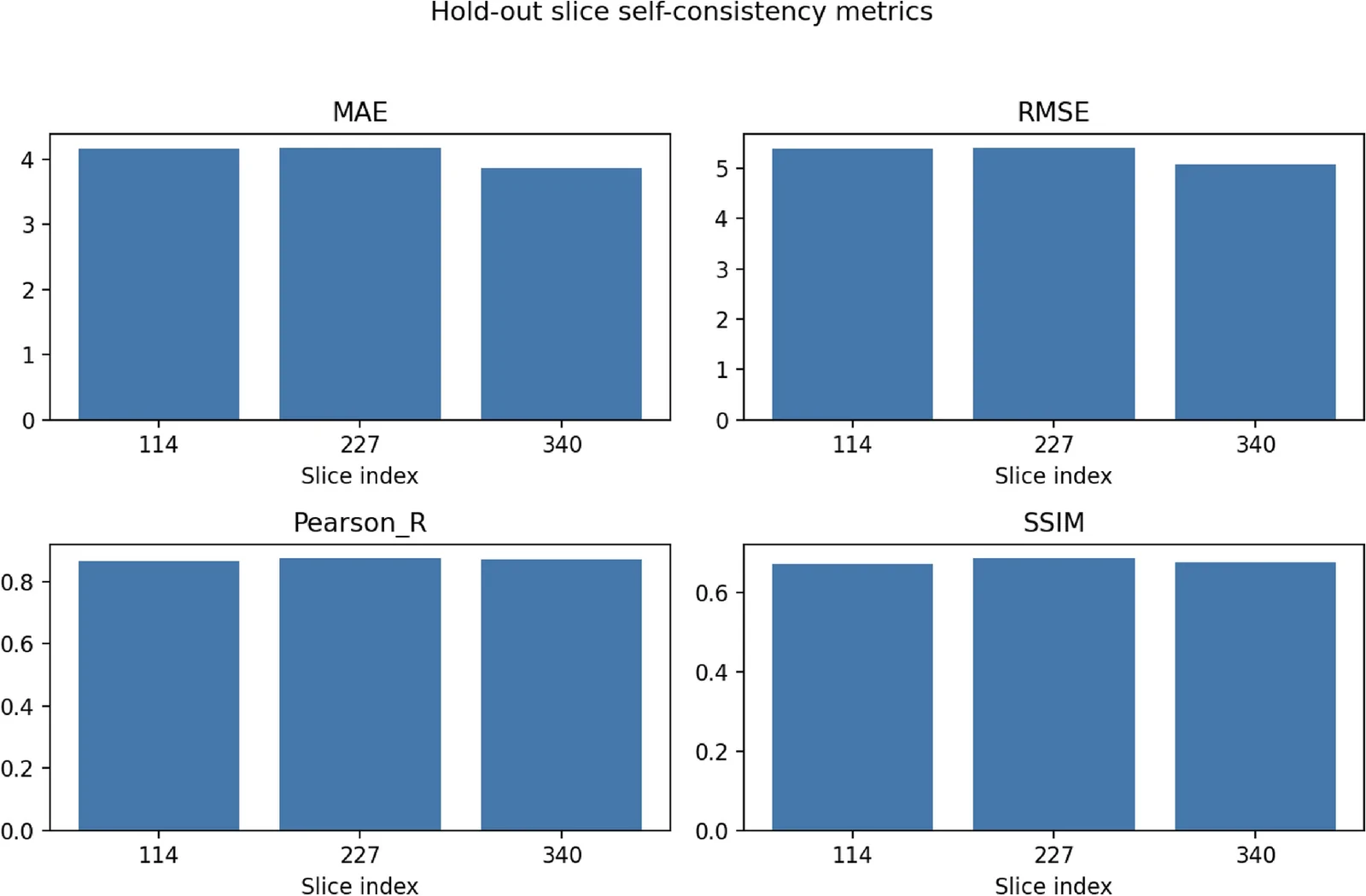
Hold-out slice evaluation of inverse grayscale self-consistency for the CNN-derived porosity field. MAE, RMSE, Pearson correlation, and SSIM are reported for representative slices along the *z* direction, indicating that the grayscale-domain consistency is reproducible across multiple slices rather than restricted to a single representative image.

Second, the spatial robustness of the grayscale-domain self-consistency was evaluated using three representative hold-out slices along the *z* direction. For each slice, the CNN-derived porosity field was inversely mapped to the grayscale domain and compared with the original CT image. The hold-out slices showed stable MAE, RMSE, Pearson correlation, and SSIM values, indicating that the reconstruction behaviour was not restricted to a single representative slice (Fig. 8).

**Fig. 9:**
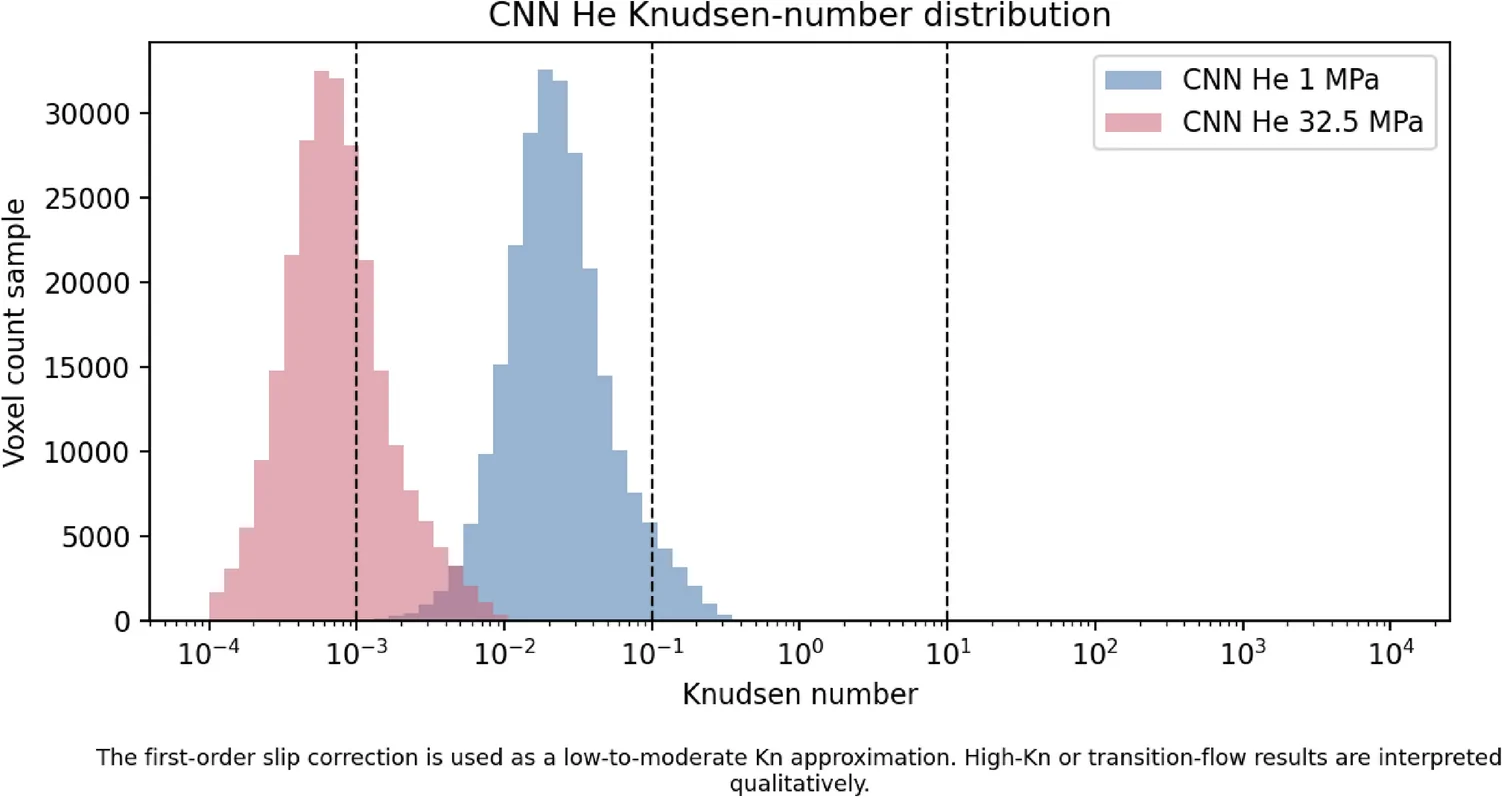
Pressure-dependent Knudsen-number and apparent-permeability assessment for the CNN-derived pore–throat field. The distributions and transport indicators show that the helium Knudsen number and the He/CH$_4$ apparent-permeability ratio decrease from low-pressure conditions toward representative deep-pressure conditions, providing a mechanism-oriented plausibility check for subsequent pore-scale transport simulations.

Finally, the reconstructed porosity field was evaluated in terms of petrophysical plausibility and Knudsen-number consistency. The CNN-derived median hydraulic radius was 808 nm, consistent with a nano- to micro-scale tight sandstone pore–throat system. Under the reconstructed pore–throat constraints, the median helium Knudsen number decreased from 0.02197 at 1 MPa to 0.000676 at 32.5 MPa, indicating a transition from slip-flow-dominated behaviour at low pressure toward near-continuum behaviour under representative deep pressure. Accordingly, the He/CH$_4$ apparent-permeability ratio decreased from 1.0457 at 1 MPa to 1.00147 at 32.5 MPa. These results support the physical plausibility of the reconstructed porosity field for mechanism-oriented pore-scale transport simulations, while not constituting direct ground-truth validation of sub-voxel pores (Fig. 9).

## Preferential gas-phase migration and slippage mechanisms in tight pore–throat systems

Gas transport in tight sandstone reservoirs is governed by the coupled effects of pore–throat geometry and non-continuum flow behaviour. Based on the reconstructed sub-voxel porosity field (Section "Sub-voxel pore structure reconstruction"), a pore-scale geometrical–physical workflow was used to quantify helium preferential migration and the molecular-size-controlled relative slip advantage of He over CH$_4$ across changing pressure conditions, including representative deep-reservoir high-pressure conditions.

### Sub-voxel pore-scale geometrical–physical fluid model

The reconstructed porosity field $\hat{\phi }(\textbf{x})$ was first transformed into physically meaningful pore–throat parameters through a geometry–flow mapping.

#### Specific surface area and geometric pore radius

The local specific surface area was estimated from the gradient magnitude of the reconstructed porosity field. To avoid singular behaviour in locally homogeneous pore regions where $|\nabla \hat{\phi }(\textbf{x})|\rightarrow 0$, a lower bound was applied and the regularized specific surface area was defined as:

18 $$\begin{aligned} S_{v,\textrm{reg}}(\textbf{x})=\max \left( \frac{4\,|\nabla \hat{\phi }(\textbf{x})|}{\Delta x},\,S_{v,\min }\right) \end{aligned}$$

where *Δ x* is the voxel resolution and $S_{v,\min }=10^{2}\,\textrm{m}^{-1}$ is a numerical lower bound for the specific surface area. This regularization suppresses nonphysical singular radii in nearly homogeneous pore regions.

#### Tight-reservoir constraints and hydraulic radius

To account for the strong confinement effects in tight sandstone and to avoid singular values in subsequent transport calculations, a cutoff-based porosity constraint and hydraulic-radius truncation were introduced.

First, a percolation threshold was applied to define a cutoff-based effective porosity:

19 $$\begin{aligned} \phi _{\textrm{cut}}(\textbf{x})={\left\{ \begin{array}{ll} \hat{\phi }(\textbf{x}), & \hat{\phi }(\textbf{x})>\phi _c,\\ \varepsilon , & \hat{\phi }(\textbf{x})\le \phi _c, \end{array}\right. } \end{aligned}$$

where $\phi _c=0.035$ is the porosity cutoff threshold and *\varepsilon* is a small numerical constant.

The geometric pore radius was then calculated as:

20 $$\begin{aligned} r_{\textrm{geo}}(\textbf{x})=\frac{2\phi _{\textrm{cut}}(\textbf{x})}{S_{v,\textrm{reg}}(\textbf{x})} \end{aligned}$$

The hydraulic throat radius was obtained using the throat-constriction factor:

21 $$\begin{aligned} r_{\textrm{hyd}}(\textbf{x})=\beta _t\,r_{\textrm{geo}}(\textbf{x}) \end{aligned}$$

where $\beta _t = 0.08$ is the throat-constriction factor used in the hydraulic-radius model. This parameter is only used to scale the geometric pore radius into an effective flow-conducting throat radius.

Finally, the hydraulic radius was bounded to avoid nonphysical values:

22 $$\begin{aligned} r_{\textrm{hyd}}(\textbf{x})=\min \!\left[ \max \!\left( r_{\textrm{hyd}}(\textbf{x}),r_{\min }\right) ,\,r_{\max }\right] \end{aligned}$$

In the numerical implementation, $r_{\min }=5~\textrm{nm}$ and $r_{\max }=10^{-4}~\textrm{m}$ were used to remove unresolved numerical artefacts and prevent singular values in the subsequent Knudsen number and permeability calculations.

#### Intrinsic permeability model

The geometric radius, hydraulic throat radius, and radius truncation definitions in Eqs. (20)–(22) are implementation-specific geometry–flow mappings introduced in this study to convert the reconstructed effective porosity field into pore-throat-scale transport parameters. The intrinsic permeability in Eq. (23) is a modified Kozeny–Carman-type relationship, in which the characteristic pore size is represented by the effective hydraulic throat radius and the porosity term is represented by cutoff-based effective porosity. The Kozeny–Carman relation is a classical permeability–porosity model for flow through porous or granular media^55,56^, but the present form is modified for the reconstructed tight-sandstone pore-throat field rather than used as a direct packed-bed equation.

23 $$\begin{aligned} k_{\textrm{int}}(\textbf{x})=\frac{r_{\textrm{hyd}}^{2}(x)\,\phi _{\textrm{cut}}(\textbf{x})}{4c\tau ^{2}} \end{aligned}$$

where *c* is the Kozeny constant and *τ* is tortuosity.

This formulation reduces permeability compared with conventional models, reflecting the ultra-low permeability nature of tight sandstone.

#### Multicomponent transport framework

**Fig. 10:**
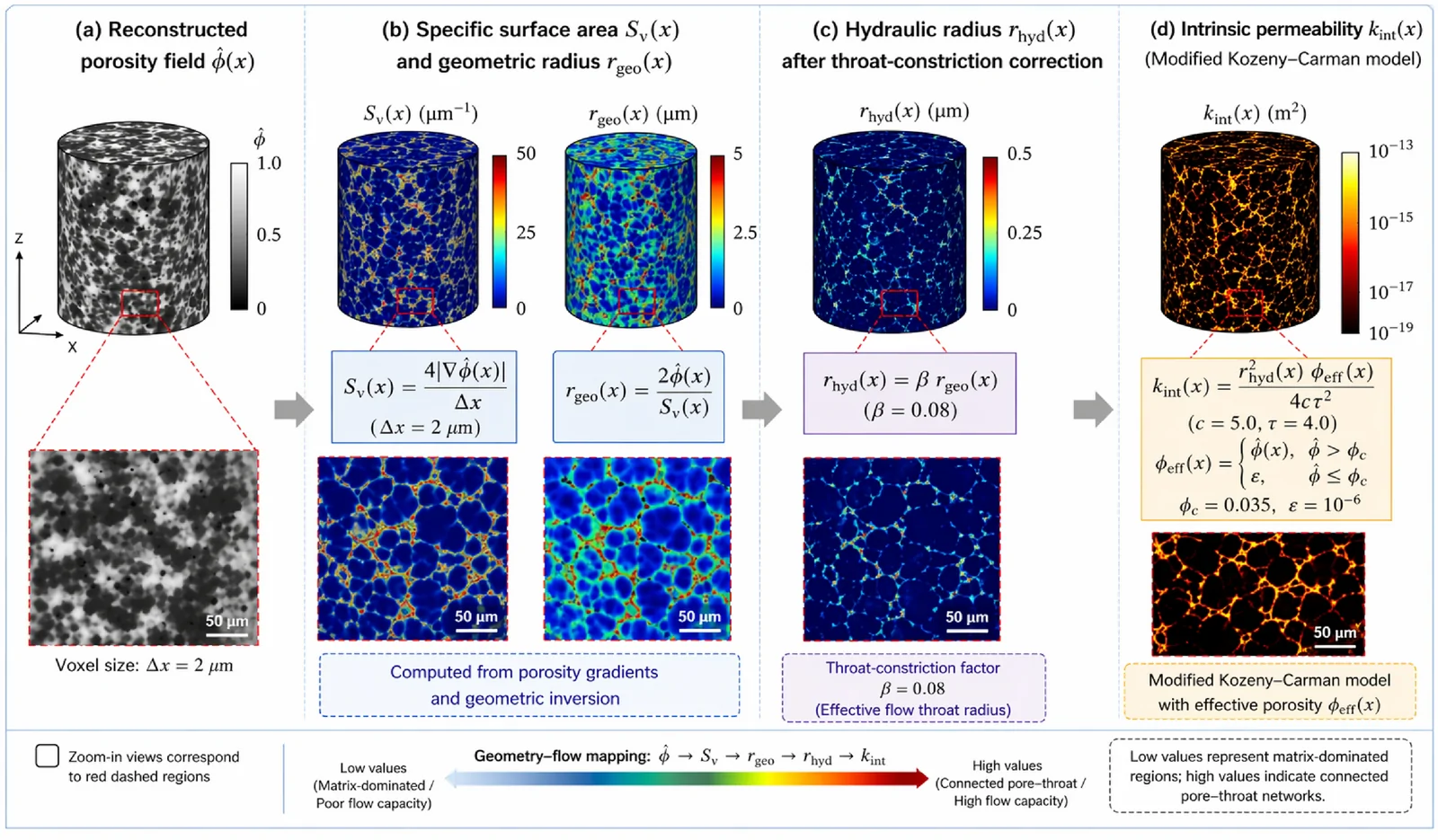
Pore-scale geometrical–physical modelling workflow for tight sandstone. (**a**) Reconstructed sub-voxel porosity field obtained from the physics-constrained convolutional neural network. (b) Specific surface area and geometric pore radius derived from the porosity gradient field, representing the transition from pore structure to geometric characterization. (**c**) Hydraulic radius after throat-constriction correction, reflecting the effective flow-conducting pore–throat size in tight sandstone. (**d**) Intrinsic permeability field calculated using a modified Kozeny–Carman model incorporating cutoff-based effective porosity constraints. Zoom-in views correspond to the red dashed regions. The workflow illustrates the geometry–flow mapping from porosity to transport properties, capturing the progressive transformation from pore structure to flow capacity. Low values represent matrix-dominated regions with limited connectivity, whereas high values indicate well-connected pore–throat networks with enhanced flow potential.

Gas transport was simulated using a lattice Boltzmann formulation based on the BGK-type collision operator and a Shan–Chen-type multicomponent interaction framework^57–59^. Eqs. (24)–(25) are therefore literature-based LBM equations, while the specific parameter choices and coupling to the reconstructed effective porosity field are implementation-specific to this study (Fig. 10).

24 $$\begin{aligned} f_i^{k}(x+c_i\Delta t,t+\Delta t)=f_i^{k}(x,t)-\frac{1}{\tau _k}\left[ f_i^{k}(x,t)-f_i^{k,\textrm{eq}}(x,t)\right] \end{aligned}$$

where *k* denotes gas components (He and CH$_4$).

Macroscopic variables are given by:

25 $$\begin{aligned} \rho _k=\sum _i f_i^{k}, \qquad u_k=\frac{1}{\rho _k}\sum _i f_i^{k}c_i \end{aligned}$$

### Knudsen number distribution and flow regime evolution

#### Mean free path and Knudsen number

The molecular mean free path and Knudsen number definitions in Eqs. (26)–(28) follow standard gas kinetic theory and are used here to classify local flow regimes in nano-confined pore throats^60^. The flow-regime thresholds in Eq. (29) are used as conventional regime boundaries for continuum, slip, transition, and free-molecular flow.

The molecular mean free path is defined as:

26 $$\begin{aligned} \lambda =\frac{k_B T}{\sqrt{2}\,\pi d^2 P} \end{aligned}$$

where *d* is molecular diameter.

The Knudsen number at voxel scale is:

27 $$\begin{aligned} \textrm{Kn}(\textbf{x})=\frac{\lambda }{r_{\textrm{hyd}}(\textbf{x})} \end{aligned}$$

A representative Knudsen number for the entire domain was further defined using the median hydraulic radius:

28 $$\begin{aligned} \textrm{Kn}_{\textrm{median}}=\frac{\lambda }{\textrm{median}(r_{\textrm{hyd}})} \end{aligned}$$

#### Pore-throat radius distribution and flow-regime classification

Flow regimes were classified using the local Knudsen number:

29 $$\begin{aligned} {\left\{ \begin{array}{ll} \textrm{Kn}<0.001, & \text {continuum flow},\\ 0.001<\textrm{Kn}<0.1, & \text {slip flow},\\ 0.1<\textrm{Kn}<10, & \text {transition flow},\\ \textrm{Kn}>10, & \text {free molecular flow}. \end{array}\right. } \end{aligned}$$

**Fig. 11:**
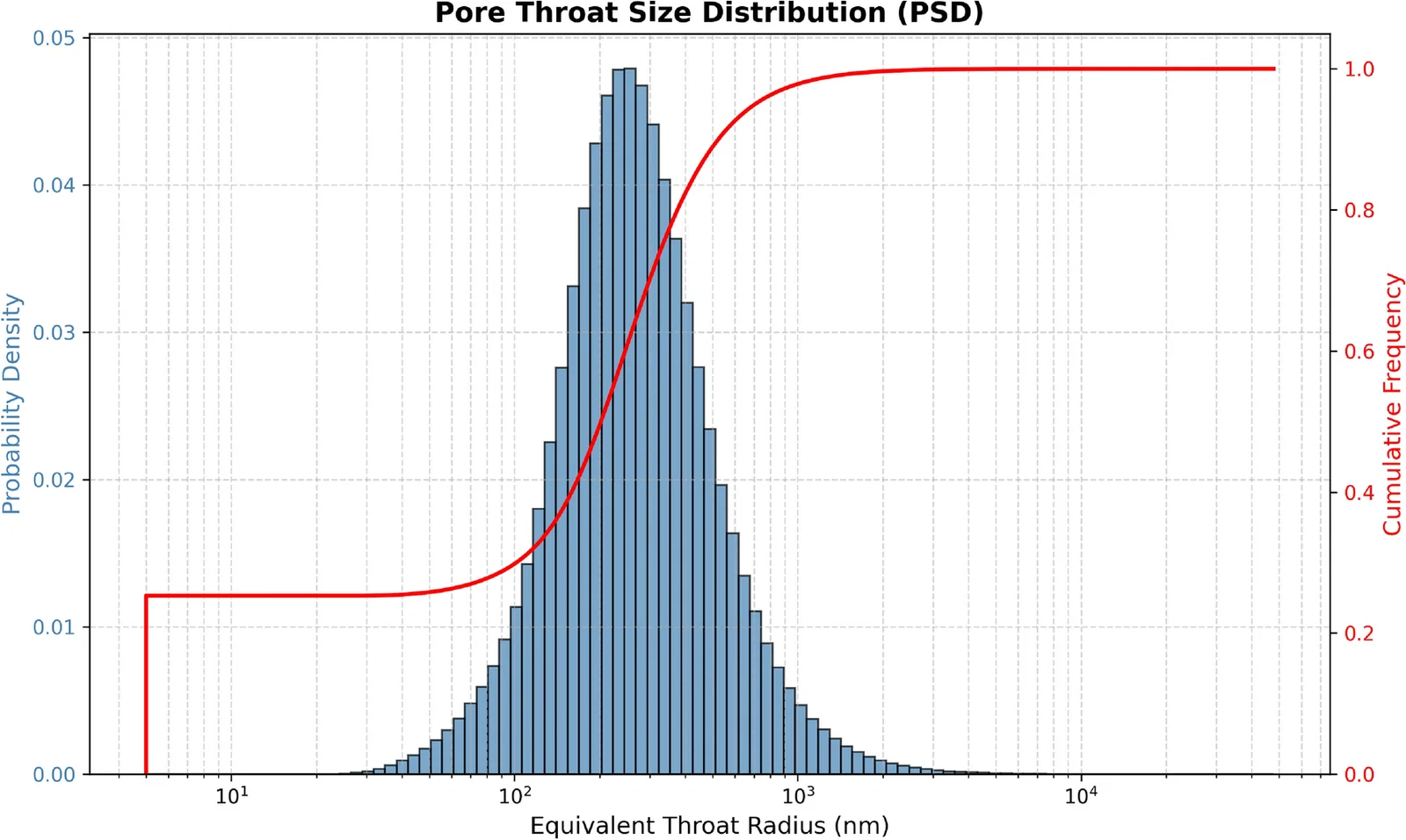
Probability density function (PDF) and cumulative distribution function (CDF) of the equivalent hydraulic pore-throat radius derived from the reconstructed effective porosity field. The PDF shows a right-skewed distribution with a dominant nano- to submicron-scale peak, and the CDF indicates that most effective flow channels are smaller than 1 *μ* m. This pore-throat radius distribution supports the tight-sandstone character of the reconstructed pore network and provides the geometric basis for subsequent Knudsen-number calculations.

The equivalent hydraulic pore-throat radius distribution derived from the reconstructed effective porosity field is shown in Fig. 11. This figure presents the probability density function and cumulative distribution function of $r_{\textrm{hyd}}$, rather than the Knudsen-number distribution itself. The radius distribution provides the geometric basis for evaluating Knudsen number because, for a given gas species and pressure–temperature condition, $\textrm{Kn}$ is inversely proportional to the effective hydraulic throat radius. Therefore, smaller nano-scale throats are expected to be more sensitive to slip-flow and Knudsen effects than larger micron-scale pores.

#### Pressure-dependent Knudsen-number distribution and slip-flow permeability enhancement

**Fig. 12:**
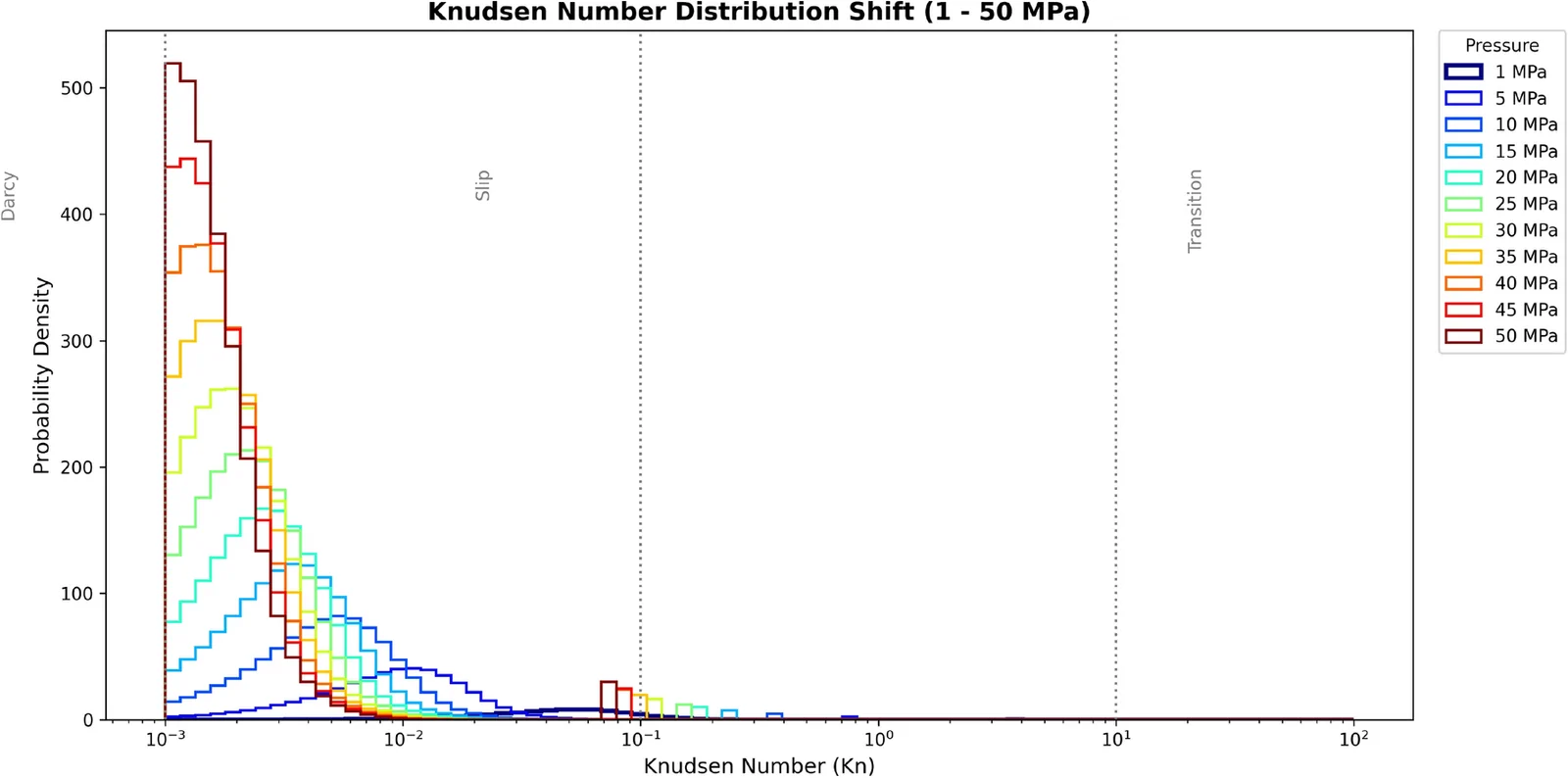
Pressure-dependent Knudsen-number distribution of helium within the reconstructed pore network under reservoir pressures of 1–50 MPa. At low pressures of 1–5 MPa, a considerable fraction of pore throats falls within the slip-flow and low-end transition-flow ranges. As pressure increases, the Kn distribution shifts progressively toward lower values because of the reduced molecular mean free path. Under representative deep-reservoir pressure, the absolute contribution of slip-flow enhancement is therefore strongly suppressed.

Using the hydraulic pore-throat radius distribution shown in Fig. 11, the pressure-dependent Knudsen-number distributions were calculated for helium under reservoir pressures of 1–50 MPa. These results are shown in Fig. 12. At low pressures of 1–5 MPa, a substantial fraction of the calculated Kn values falls within the slip-flow and low-end transition-flow ranges, indicating that molecule–wall interactions can enhance apparent permeability in nano-confined pore throats. With increasing pressure, the gas mean free path decreases and the Kn distribution shifts toward lower values. At representative deep-reservoir pressure, the distribution is displaced toward the continuum or near-continuum regime, indicating that the absolute slip-flow contribution is strongly suppressed.

To quantify this pressure-dependent non-continuum effect, the apparent permeability was calculated using a first-order Klinkenberg-type correction^20,61–63^:

30 $$\begin{aligned} k_{\textrm{app}}(\textbf{x})=k_{\textrm{int}}(\textbf{x})\left[ 1+\alpha \,\textrm{Kn}(\textbf{x})\right] , \end{aligned}$$

where $k_{\textrm{app}}$ is the apparent permeability, $k_{\textrm{int}}$ is the intrinsic permeability, and *α* is the slip coefficient. This correction is treated as a low-to-moderate Kn approximation. Therefore, Fig. 12 should be interpreted as showing the pressure-dependent evolution of the helium transport regime, whereas Fig. 11 should be interpreted as the underlying pore-throat radius distribution used to calculate the Knudsen number.

#### Applicability of the first-order slip correction

The first-order apparent-permeability correction was used as a low-to-moderate Kn approximation rather than as a complete transition-flow model. Quantitative interpretation was therefore restricted mainly to the continuum and slip-flow regimes, particularly $\textrm{Kn}<0.1$. Results falling within the transition-flow regime ($0.1<\textrm{Kn}<10$) were retained only to indicate the direction of non-continuum enhancement and were not interpreted as fully quantitative permeability predictions.

**Fig. 13:**
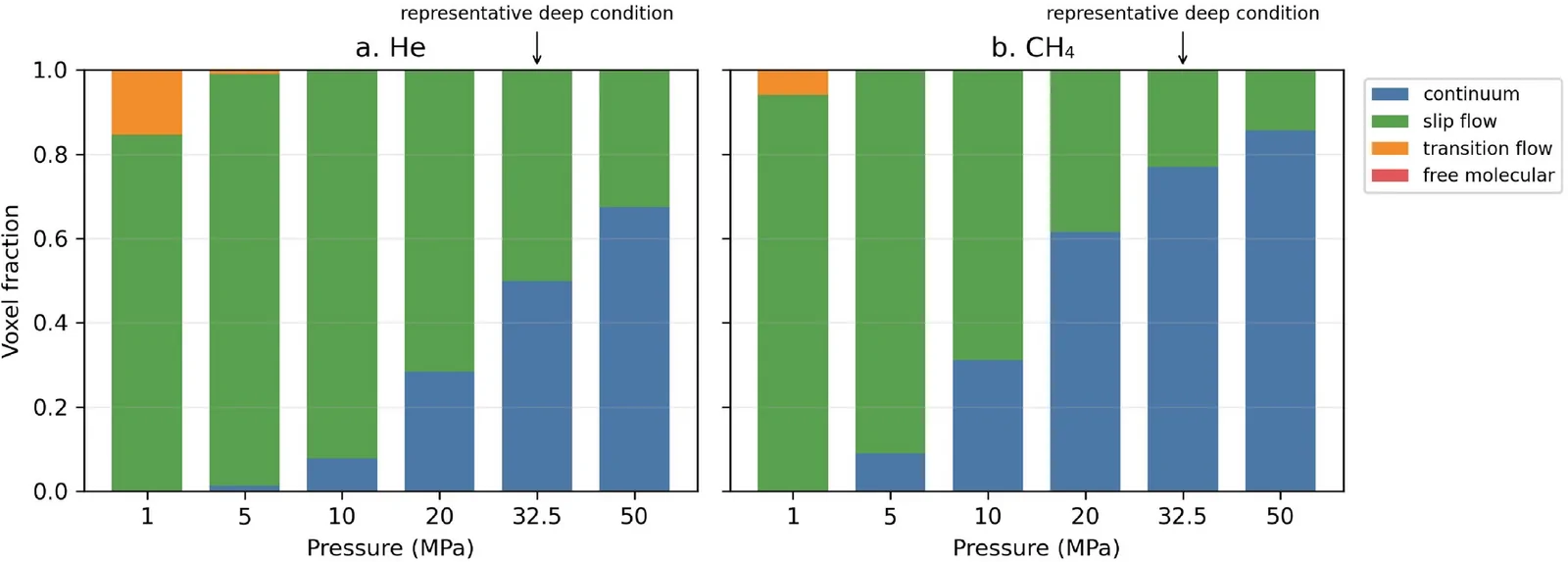
Knudsen-regime fractions and applicability of the first-order slip correction. The active pore voxels were classified into continuum flow ($\textrm{Kn}<0.001$), slip flow ($0.001\le \textrm{Kn}<0.1$), transition flow ($0.1\le \textrm{Kn}<10$), and free-molecular flow ($\textrm{Kn}\ge 10$). The first-order apparent-permeability correction was treated as a low-to-moderate Kn approximation. Cases with a substantial transition-flow contribution were interpreted as qualitative or semi-quantitative trends rather than fully quantitative permeability predictions. The representative deep condition of 32.5 MPa falls entirely within the continuum/slip-flow range for He.

To define the applicability range of this correction, the Knudsen-regime fractions were quantified at pressures from 1 to 50 MPa (Fig. 13). At 1 MPa, the median He Knudsen number is 0.0326 and approximately 15.4% of He-bearing active voxels fall into the transition-flow regime. Therefore, the 1 MPa case is interpreted as a semi-quantitative estimate. At 5 MPa and above, more than 98.9% of He-bearing active voxels are within the continuum/slip-flow range, supporting the use of the first-order correction as a low-to-moderate Kn approximation. Under the representative deep condition of 32.5 MPa, the median He Knudsen number decreases to 0.0010, with no transition-flow voxels detected in the active domain.

**Fig. 14:**
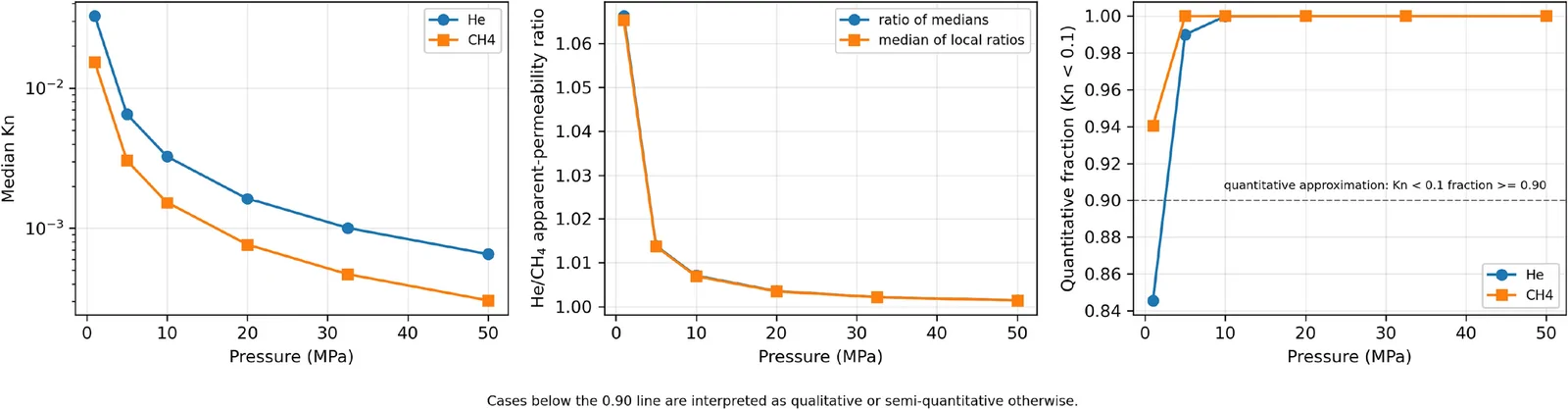
Pressure-dependent He/CH$_4$ mobility contrast under the low-to-moderate Kn approximation. The median Knudsen number decreases with increasing pressure, and the He/CH$_4$ apparent-permeability ratio approaches unity under deep-pressure conditions. The fraction of active voxels with $\textrm{Kn}<0.1$ was used as an applicability diagnostic for the first-order correction. Cases below the 0.90 threshold were interpreted as semi-quantitative or qualitative trends, whereas cases above this threshold were treated as low-to-moderate Kn approximations.

The pressure-dependent applicability analysis also constrains the interpretation of the He/CH$_4$ apparent-permeability contrast (Fig. 14). The He/CH$_4$ apparent-permeability ratio decreases from 1.066 at 1 MPa to 1.002 at 32.5 MPa and 1.001 at 50 MPa. This indicates that the relative He mobility advantage is amplified under low-pressure conditions but becomes very weak under representative deep pressure. Accordingly, transition-flow and high-Kn results are discussed only as trend-level indicators, whereas deep-pressure results are treated as low-to-moderate Kn approximations.

#### Preferential helium migration mechanism

Eqs. (31)–(33) are algebraic inequalities derived in this study from the molecular-diameter dependence of mean free path under identical pore-throat radius, temperature, and pressure conditions. They are not introduced as new transport laws. Instead, they provide a simplified mechanistic explanation for why helium can have a higher Knudsen number and slightly higher apparent permeability than methane in the same nano-confined pore geometry. Because helium has a smaller molecular diameter than methane, it has a larger molecular mean free path under the same temperature and pressure conditions:

31 $$\begin{aligned} \lambda _{\textrm{He}}>\lambda _{\textrm{CH}_4} \end{aligned}$$

Therefore, for the same hydraulic throat radius, helium has a higher Knudsen number:

32 $$\begin{aligned} \textrm{Kn}_{\textrm{He}}>\textrm{Kn}_{\textrm{CH}_4} \end{aligned}$$

and a higher apparent permeability:

33 $$\begin{aligned} k_{\textrm{app,He}}>k_{\textrm{app,CH}_4} \end{aligned}$$

This relationship explains why He can exhibit a molecular-size-controlled relative slip advantage over CH$_4$ in nano-confined pore throats.

Figure 12 shows that this slip-related transport regime is strongly pressure dependent. At low pressures (1–5 MPa), the Kn distribution of helium is concentrated in the slip-flow and transition regimes. Increasing pressure shifts the Kn distribution toward the low-Kn region and progressively toward the continuum-flow regime. Thus, under representative deep high-pressure conditions, the absolute slip contribution becomes much weaker.

**Fig. 15:**
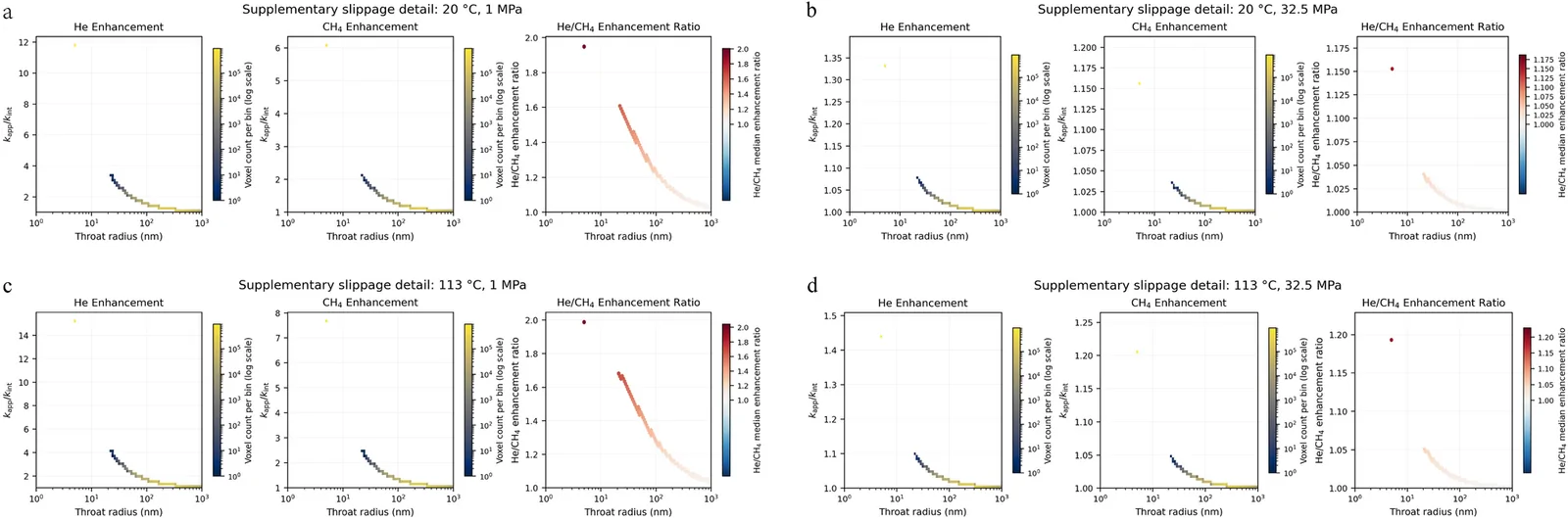
Pressure–temperature dependence of He apparent-permeability enhancement. Two-dimensional voxel-count heatmaps showing the relationship between hydraulic throat radius and the apparent-permeability enhancement factor $k_{\textrm{app}}/k_{\textrm{int}}$ for He under four temperature–pressure conditions: (**a**) 20 $^\circ$ C, 1 MPa; (**b**) 20 $^\circ$ C, 32.5 MPa; (**c**) 113 $^\circ$ C, 1 MPa; and (**d**) 113 $^\circ$ C, 32.5 MPa. The panels were replotted using a high-contrast, color-blind-friendly color scale and a logarithmic voxel-count colorbar to improve readability. Vertical reference lines indicate the nano-throat domain and the transition toward micron-scale pores. Each panel was locally scaled to improve visualization; the comparison shows that slip enhancement is strongest in small throats and under low-pressure conditions, whereas enhancement is strongly reduced at 32.5 MPa.

**Fig. 16:**
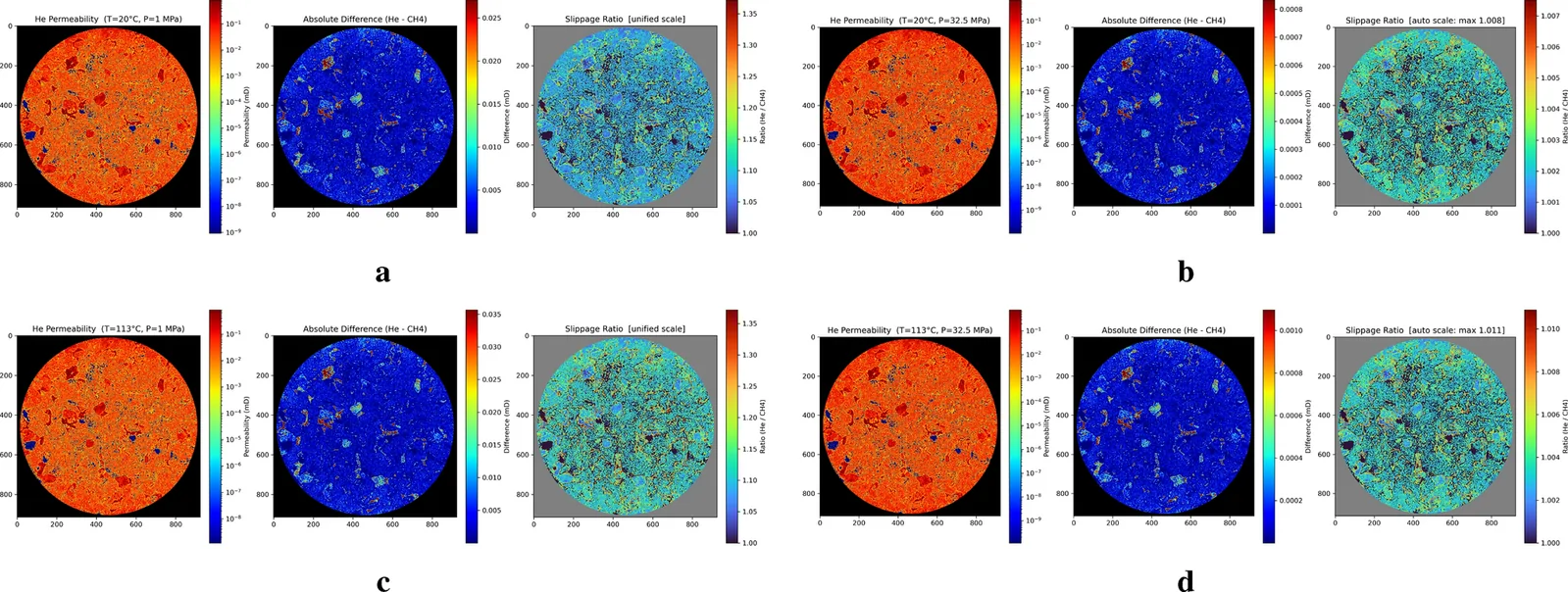
Spatial decomposition of He–CH$_4$ apparent-permeability contrast under four temperature–pressure conditions: (**a**) 20 $^\circ$ C, 1 MPa; (**b**) 20 $^\circ$ C, 32.5 MPa; (**c**) 113 $^\circ$ C, 1 MPa; and (**d**) 113 $^\circ$ C, 32.5 MPa. In each case, the composite map compares the intrinsic permeability field, the voxel-scale absolute difference ($k_{\textrm{app,He}}-k_{\textrm{app,CH}_4}$), and the voxel-scale ratio ($k_{\textrm{app,He}}/k_{\textrm{app,CH}_4}$). Elevated ratio values are local values concentrated mainly in nano-confined throats, pore margins, and microfracture-like narrow regions. Local ratios may approach or exceed 1.3 under the 1 MPa cases, whereas under the representative deep condition of 113 $^\circ$ C and 32.5 MPa the local ratio is close to unity, approximately 1.008–1.011. These local ratios should be distinguished from the domain-averaged permeability contrast shown in Figure 17.

The direct He–CH$_4$ comparison is provided by Figs. 15 and 16, which compare slippage sensitivity and apparent permeability contrast under four temperature–pressure conditions. Together, these results show that helium retains a molecular-size-controlled relative slip advantage over CH$_4$. The advantage is larger at low pressure and markedly reduced under representative deep-reservoir conditions.

Under these model assumptions, helium is expected to be relatively more mobile than methane in nano-confined throats, especially at low pressure. This provides a pore-scale mechanism that may contribute to helium enrichment where methane contacts helium-bearing formation water in tight sandstone.

### Pressure–temperature dependence of preferential helium mobility

To test whether the He–CH$_4$ mobility contrast persists under representative deep-reservoir conditions, four pressure–temperature scenarios were compared: 20 $^\circ$ C and 1 MPa, 20 $^\circ$ C and 32.5 MPa, 113 $^\circ$ C and 1 MPa, and 113 $^\circ$ C and 32.5 MPa. These cases separate the effects of pressure and temperature and explicitly include the representative deep condition used in this study.

Figure 15 shows that throat radius is the primary geometric control on $k_{\textrm{app}}/k_{\textrm{int}}$. Nano-scale throats show stronger enhancement than micron-scale pores in all four cases. However, pressure strongly controls the magnitude of enhancement. At 1 MPa, the enhancement is pronounced, whereas at 32.5 MPa it decreases sharply. Temperature produces a secondary modulation through the molecular mean free path, but it does not reverse the pressure-controlled trend. In this sense, nano-throats are the main locations where pressure-dependent non-continuum effects are activated.

Figure 16 provides the direct spatial comparison between helium and methane under four temperature–pressure conditions: 20 $^\circ$ C and 1 MPa, 20 $^\circ$ C and 32.5 MPa, 113 $^\circ$ C and 1 MPa, and 113 $^\circ$ C and 32.5 MPa. In each case, the plotted quantities are the voxel-scale apparent-permeability difference, ($k_{\textrm{app,He}}-k_{\textrm{app,CH}_4}$), and the voxel-scale apparent-permeability ratio, ($k_{\textrm{app,He}}/k_{\textrm{app,CH}_4}$), within the connected effective pore-throat network. The largest local ratios occur in nano-confined throats and pore-margin regions under the 1 MPa cases, where selected local values approach or exceed 1.3. These local high-ratio values should not be interpreted as a domain-averaged permeability increase. Under the representative deep condition of 113 $^\circ$ C and 32.5 MPa, the local ratio is close to unity, approximately 1.008–1.011 in the connected nano-throat regions, indicating that the relative mobility advantage of helium persists but is strongly suppressed by high pressure.

These results indicate that helium preferential migration should be interpreted as a pressure-dependent relative slip-flow advantage rather than a uniform deep-reservoir slippage response. Low pressure or pressure depletion amplifies the He–CH$_4$ contrast, whereas deep high pressure reduces the absolute magnitude of slip enhancement. Under the representative deep condition of 113 $^\circ$ C and 32.5 MPa, the absolute slip enhancement is greatly reduced, but helium still shows a slightly higher apparent permeability than methane in nano-confined pore throats.

### Pressure-dependent apparent permeability and Klinkenberg-type scaling

To relate the pore-scale non-continuum transport analysis to an upscaled permeability response, the apparent permeability was calculated over the reconstructed pore network under different pressure conditions. The pressure dependence of apparent permeability in Eqs. (34)–(35) follows the Klinkenberg-type relationship between gas apparent permeability and inverse pressure^20,61^. The high-pressure limit in Eq. (35) is algebraically derived from Eq. (34), indicating convergence toward intrinsic permeability as pressure increases. In the revised manuscript, this comparison is interpreted as a consistency check for the adopted pore-scale slip-flow correction rather than as independent experimental validation. The pressure dependence was interpreted using the classical Klinkenberg-type relationship:

34 $$\begin{aligned} k_{\textrm{app}} = k_{\textrm{int}}\left( 1+\frac{b}{P}\right) , \end{aligned}$$

where $k_{\textrm{app}}$ is the apparent permeability, $k_{\textrm{int}}$ is the intrinsic permeability, *P* is pressure, and *b* is the Klinkenberg slip factor. In this formulation, a linear relationship between $k_{\textrm{app}}$ and 1/*P* indicates that the pressure-dependent permeability enhancement can be described by a first-order slip-flow correction.

The corresponding high-pressure limit is:

35 $$\begin{aligned} \lim _{P\rightarrow \infty } k_{\textrm{app}} = k_{\textrm{int}} \end{aligned}$$

**Fig. 17:**
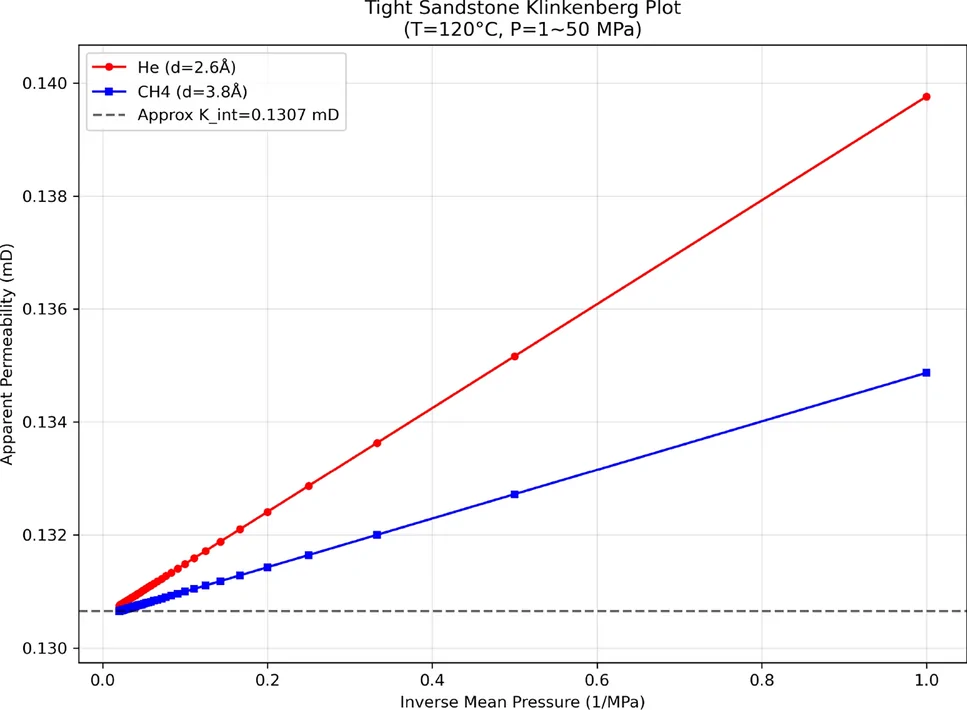
Klinkenberg-type apparent-permeability scaling for helium and methane over the tested pressure range of 1–50 MPa. The plotted values represent upscaled apparent permeability calculated over the connected effective pore-throat network. The He–CH$_4$ contrast is quantified as $\Delta k_{\textrm{rel}}=(\langle k_{\textrm{app,He}}\rangle -\langle k_{\textrm{app,CH}_4}\rangle )/\langle k_{\textrm{app,CH}_4}\rangle \times 100\%$, where $\langle k_{\textrm{app}}\rangle$ is the domain-averaged apparent permeability. The largest contrast occurs at the lowest-pressure condition tested (1 MPa), where the relative difference is on the order of 10% depending on the temperature case. At the representative deep-reservoir pressure of 32.5 MPa, the contrast is strongly reduced, and both gases converge toward the intrinsic permeability in the high-pressure limit. This result indicates a pressure-dependent relative mobility contrast rather than uniformly strong helium slippage under deep high-pressure conditions.

As shown in Fig. 17, the simulated apparent permeability decreases approximately linearly with decreasing 1/*P* for both helium and methane. This trend supports the internal consistency between the reconstructed pore-throat geometry, the slip-flow correction, and the expected Klinkenberg-type pressure dependence. However, this comparison should be interpreted as a consistency check for the adopted pore-scale transport model rather than as an independent experimental validation.

The fitted pressure dependence differs between helium and methane. Helium shows a steeper $k_{\textrm{app}}$–1/*P* trend than methane, corresponding to a larger effective slip factor. This difference is consistent with the smaller molecular diameter of helium, which gives helium a longer molecular mean free path and a larger Knudsen number than methane under the same pore-throat radius, temperature, and pressure conditions. Consequently, the apparent permeability of helium is slightly higher than that of methane over the simulated pressure range.

The He–CH$_4$ permeability contrast is most evident in the lowest-pressure case tested. In Fig. 17, the reported permeability contrast refers to the upscaled apparent permeability calculated over the connected effective pore-throat network, expressed as

36 $$\begin{aligned} \Delta k_{\textrm{rel}}= \frac{\langle k_{\textrm{app,He}}\rangle -\langle k_{\textrm{app,CH}_4}\rangle }{\langle k_{\textrm{app,CH}_4}\rangle }\times 100\% , \end{aligned}$$

where $\langle k_{\textrm{app}}\rangle$ denotes the domain-averaged apparent permeability obtained from the reconstructed pore network. Under the lowest-pressure condition of the tested range (1 MPa), this relative contrast is on the order of 10%, depending on the temperature case. This value represents an upscaled model-derived apparent-permeability contrast for the reconstructed pore network, not a universal permeability increase for all pore throats. At the representative deep-reservoir pressure of 32.5 MPa, the He–CH$_4$ contrast decreases sharply and both gases approach the intrinsic permeability in the high-pressure limit.

### Parameter sensitivity and calibration limits

To evaluate the influence of empirical and semi-empirical parameters on the pore-scale results, a compact one-at-a-time sensitivity analysis was performed for the throat-constriction factor $\beta _t$, porosity cutoff $\phi _c$, minimum hydraulic-radius cutoff $r_{\min }$, bound-water porosity threshold $\phi _{\textrm{bound}}$, and residual water saturation $S_{wr}$. The representative slice used for the sensitivity analysis has a mean porosity of 0.099, close to the mean porosity of the 3D reconstructed volume. The geometry-related parameters $\beta _t$, $\phi _c$, and $r_{\min }$ were evaluated in terms of hydraulic-radius statistics, Knudsen-number distributions, and He/CH$_4$ apparent-permeability ratio, whereas $\phi _{\textrm{bound}}$ and $S_{wr}$ were evaluated using water-volume and extraction-response proxies.

Among the geometry-related parameters, $\beta _t$ has the strongest influence on the He/CH$_4$ apparent-permeability ratio because it directly scales the effective hydraulic radius. Decreasing $\beta _t$ from 0.08 to 0.04 increases the median He Knudsen number at 1 MPa from 0.040 to 0.080 and increases the He/CH$_4$ apparent-permeability ratio from 1.080 to 1.150, whereas increasing $\beta _t$ to 0.12 reduces the ratio to 1.054. In contrast, changing $\phi _c$ from 0.02 to 0.05 substantially changes the flow-active voxel fraction, but only weakly affects the median Kn and He/CH$_4$ ratio (Table 4).

**Table 4:** Compact sensitivity summary for pore-geometry parameters.

Parameter	Tested range	Main affected quantity	Maximum response	Interpretation
$\beta _t$	0.04–0.12	Hydraulic radius, Kn, He/CH$_4$ ratio	He/CH$_4$ ratio changes from 1.150 to 1.054 at 1 MPa	Most influential geometry parameter
$\phi _c$	0.02–0.05	Active pore fraction	Active pore fraction changes from 0.967 to 0.601	Minor effect on median Kn
$r_{\min }$	1–20 nm	High-Kn tail	No median-Kn change because cutoff $<P_{10}$ radius	Dataset-specific robustness
$r_{\min }$ stress	1–200 nm	Flow-regime fractions	Transition-flow fraction decreases at 200 nm	Truncation matters once cutoff enters radius distribution

The minimum hydraulic-radius cutoff $r_{\min }$ was examined separately because it can artificially truncate small pore throats and shift the high-Kn tail. Within the physically plausible range of 1–20 nm, $r_{\min }$ does not change the median Kn distribution in the representative slice because the active hydraulic-radius distribution has $P_{10}\approx 188.7$ nm. This result should therefore be interpreted as a dataset-specific robustness check rather than evidence that $r_{\min }$ is universally unimportant. An extended stress test with $r_{\min }$ up to 200 nm further shows that hydraulic-radius truncation begins to affect flow-regime fractions when the cutoff intersects the sampled hydraulic-radius distribution. Nevertheless, the pressure-controlled trend remains unchanged: the He/CH$_4$ contrast is amplified at low pressure but strongly suppressed under deep high-pressure conditions.

**Fig. 18:**
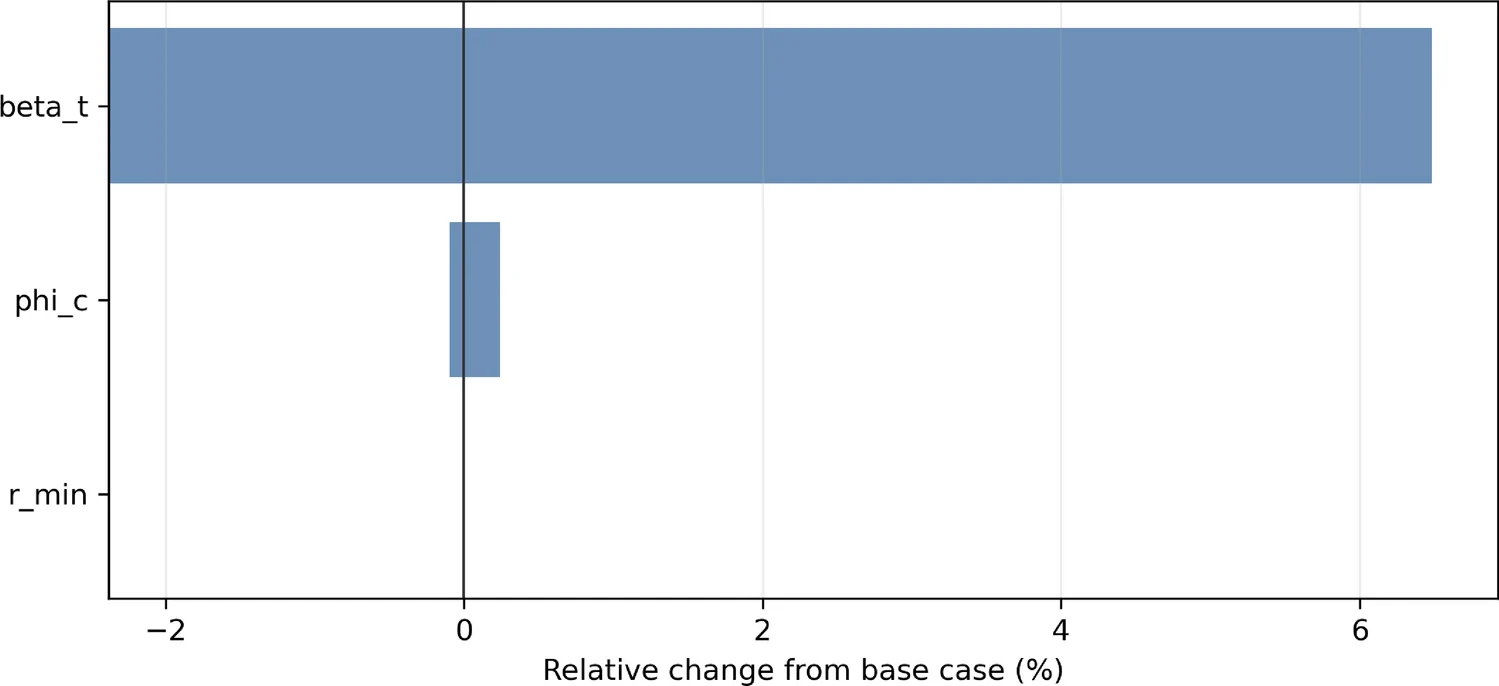
Sensitivity of the He/CH$_4$ apparent-permeability ratio to pore-geometry parameters. The tornado plot shows the relative change from the base case for the throat-constriction factor $\beta _t$, porosity cutoff $\phi _c$, and minimum hydraulic-radius cutoff $r_{\min }$. $\beta _t$ has the largest influence because it directly scales the effective hydraulic radius and therefore controls the Knudsen-number distribution. In contrast, $\phi _c$ and $r_{\min }$ have smaller effects within the tested range. The results indicate that the absolute magnitude of the He/CH$_4$ mobility contrast is parameter dependent, whereas the pressure-controlled trend remains unchanged.

This pore-geometry sensitivity is summarized in Fig. 18.

These sensitivity tests indicate that the absolute magnitude of Kn-related enhancement and extraction response is parameter dependent. Therefore, the LBM results are interpreted as parameterized pore-scale simulations rather than fully calibrated recovery predictions.

## Multicomponent gas solubility and macroscopic stripping kinetics under deep conditions

Under deep burial conditions (*>2000* m), gas–water interactions in tight sandstone reservoirs are controlled by coupled thermodynamic partitioning and dynamic mass transfer processes. Unlike shallow systems governed mainly by equilibrium dissolution, deep environments characterized by high temperature, high pressure, and high salinity promote non-ideal behaviour of multicomponent gases. These conditions alter gas solubility, phase distribution, and interfacial transport, and therefore influence the efficiency of methane-driven helium extraction (“stripping process”).

To evaluate this mechanism quantitatively, a gas–water partitioning model was constructed by integrating a real-gas equation of state, temperature-dependent Henry solubility, Poynting correction, and salting-out effects.

### Thermodynamic model of gas solubility under high-temperature and high-pressure conditions

Deep tight gas reservoirs in the Ordos Basin are commonly characterized by temperatures exceeding 100 $^\circ$ C and pressures above 30 MPa. Under these conditions, the dissolution behaviour of gas components deviates from ideal Henry-type behaviour and must be corrected for real-gas effects, pressure compression, and salinity. The gas–water solubility model in Eq. (37) combines Henry-law-type solubility, real-gas fugacity, Poynting pressure correction, and Setschenow salinity correction. Henry-law constants and gas solubility correlations in water are commonly compiled and parameterized for geochemical and thermodynamic applications^64,65^. The salinity correction follows the Setschenow relation, which is widely used to represent salting-out effects on aqueous solubility and partitioning^66^. In this study, the equilibrium aqueous concentration, or solubility limit, of gas component *i* in formation water was denoted as $C_i^*(T,P,I)$ and calculated as:

37 $$\begin{aligned} C_i^*(T,P,I) = \boldsymbol{K}_{H,i}^{\textrm{sol}}\left( T\right) f_i^{(\textrm{atm})} \exp \!\left[ -\frac{\bar{V}_i (P-P_0)}{RT}\right] 10^{-k_{s,i} I} \end{aligned}$$

where $C_i^*(T,P,I)$ is the equilibrium aqueous concentration, or solubility limit, of gas component *i* in mol kg$_{\textrm{w}}^{-1}$. $\boldsymbol{K}_{H,i}^{\textrm{sol}}\left( T\right)$ is the temperature-dependent solubility-form Henry coefficient, $f_i^{(\textrm{atm})}$ is the fugacity of gas *i* expressed in atm, $\bar{V}_i$ is the partial molar volume, *P* is pressure, $P_0$ is the reference pressure, *R* is the gas constant, *T* is temperature, $k_{s,i}$ is the Setschenow coefficient, and *I* is ionic strength. Here, $\boldsymbol{K}_{H,i}^{\textrm{sol}}\left( T\right)$ has units of mol kg$_{\textrm{w}}^{-1}$ atm$^{-1}$ and should not be confused with the reciprocal pressure-form Henry constant. The fugacity term is converted to atm before multiplication by $\boldsymbol{K}_{H,i}^{\textrm{sol}}\left( T\right)$, whereas the Poynting exponent uses consistent SI units: *P* and $P_0$ in Pa, $\bar{V}_i$ in m$^3$ mol$^{-1}$, and *R=8.314* J mol$^{-1}$ K$^{-1}$. The reference pressure is $P_0=1$ atm. The negative sign in the Poynting and Setschenow terms follows the implementation convention used here, where both finite partial molar volume and salinity reduce the effective aqueous solubility under the adopted high-pressure approximation.

The temperature dependence of the solubility-form Henry coefficient was described using a two-parameter van’t Hoff relationship:

38 $$\begin{aligned} \log _{10} \boldsymbol{K}_{H,i}^{\textrm{sol}}\left( T\right) = A_{1,i} + \frac{A_{2,i}}{T} \end{aligned}$$

This term captures the first-order thermal control on gas dissolution. Increasing temperature generally reduces the ability of formation water to retain dissolved gases, although the magnitude of this effect differs among gas species.

Real-gas non-ideality was incorporated through fugacity rather than pressure. The fugacity of each gas was calculated as:

39 $$\begin{aligned} f_i = \phi _i P, \qquad f_i^{(\textrm{atm})}=\frac{f_i}{P_{\textrm{atm}}} \end{aligned}$$

where $\phi _i$ is the fugacity coefficient and $P_{\textrm{atm}}=101325$ Pa. The fugacity coefficient in Eqs. (39)–(40) was calculated using the Peng–Robinson equation of state^67^. The logarithmic form used to calculate the fugacity coefficient is:

40 $$\begin{aligned} \ln \phi _i = Z - 1 - \ln (Z-B) - \frac{A}{2\sqrt{2}\,B} \ln \!\left[ \frac{Z+(1+\sqrt{2})B}{Z+(1-\sqrt{2})B}\right] \end{aligned}$$

where *Z* is the compressibility factor, and *A* and *B* are the reduced attraction and co-volume parameters of the Peng–Robinson equation. In the numerical implementation, the gas or supercritical phase was represented by the largest real root of the cubic equation for *Z*, which avoids unstable liquid-like roots under high-pressure conditions.

The pressure correction was introduced using the Poynting term, which accounts for the effect of pressure on the partial molar volume of dissolved gas. Salinity was corrected using the Setschenow relation, where increasing ionic strength reduces the available free water for gas dissolution. The salting-out effect is component-dependent; it is significant for CH$_4$, CO$_2$, and N$_2$, but weak for He because of its very small molecular size and extremely low polarity.

**Fig. 19:**
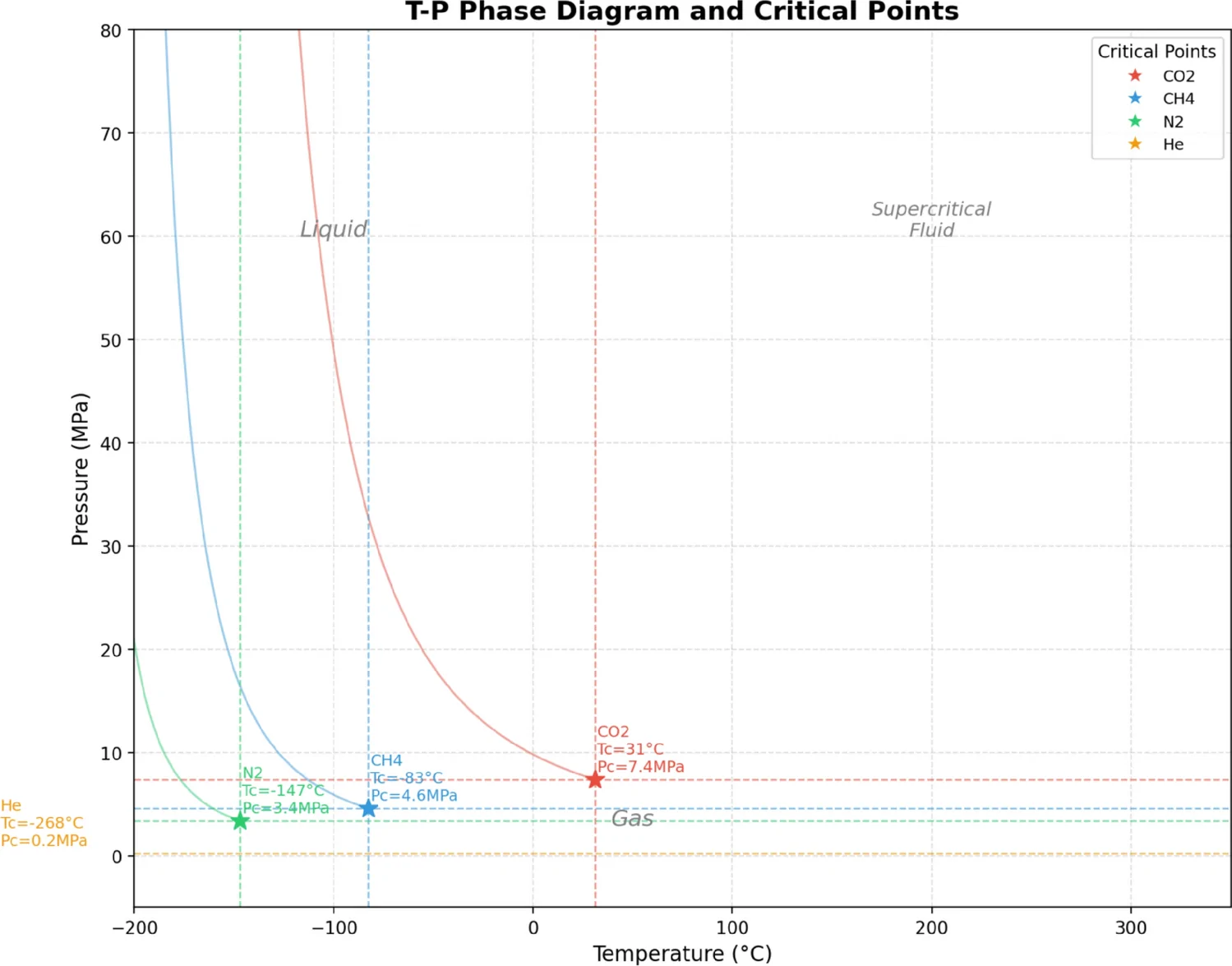
Critical temperature–pressure relationship of major gas components and representative deep reservoir conditions. He and N$_2$ have very low critical temperatures, whereas CH$_4$ and CO$_2$ enter the supercritical domain under deep reservoir conditions. When burial depth exceeds approximately 2000 m, the corresponding temperature and pressure are higher than the critical points of the major gases, indicating a supercritical multicomponent gas system favourable for gas–water mass transfer.

The ionic composition of formation water used to constrain salinity and ionic strength is summarized in Table 5. The SX well-area formation waters are dominated by high-salinity brines, with Na$^{+}$+K$^{+}$, Ca$^{2+}$, and Cl$^{-}$ as the major ions. These data provide the hydrochemical basis for calculating ionic strength and salting-out corrections in the solubility model. As shown in Fig. 19, He and N$_2$ have very low critical temperatures, whereas CH$_4$ and CO$_2$ cross their critical states when burial depth exceeds approximately 2000 m, corresponding to reservoir temperatures above 60 $^\circ$ C and pressures above 20 MPa. Therefore, under the representative deep reservoir conditions considered in this study, all four gases can be treated as supercritical or highly non-ideal gas phases. This supercritical state reduces gas–water interfacial resistance and favours methane invasion into thin water films, providing a thermodynamic basis for gas–water mass transfer and helium stripping.

**Table 5:** Ionic composition of formation water from the SX well area in the Ordos Basin^68^.

Well No.	Formation	K$^+$+Na$^+$ (mg/L)	Ca$^{2+}$ (mg/L)	Mg$^{2+}$ (mg/L)	Cl$^-$ (mg/L)	SO$_4^{2-}$ (mg/L)	HCO$_3^-$ (mg/L)	TDS (mg/L)	pH
SX-29-21	Lower submember of He 8	14210.6	1652.9	0	23019.5	1980.8	594.1	41457.8	6.5
SX-52-52	Shan 1 Member	3834	1217	308	8324	486	476	14650	6.0
SX-56-56	Upper submember of He 8	9634	3718	615	22196	810	734	37710	6.0
SX-46–50	Lower submember of He 8	6405	1420	31	9510	3645	471	21480	6.0
SX-46-39	Lower submember of He 8	7870.9	2172.9	125.4	14379.2	1980.8	375.2	26094.5	6.4
SX-56–59	Lower submember of He 8	3151	1014	62	6243	486	394	11350	6.0
SX-44-43	Lower submember of He 8	4689	1539	125	9816	0	855	17020	6.0
SX-44–53	Shan 1 Member	4669	1369	185	9313	608	680	16820	6.0
SX-51-50	Shan 1 Member	7270	2253	105	34380	2080	365	46454	6.5
SX-54-53	Lower submember of He 8	8311	1573	0	14893	1275	587	26640	6.5
SX-59–71	Lower submember of He 8	5797	1231	498	9942	3197	454	21119	6.0
SX-54–64	Shan 1 Member	6860	1847	125	11075	3689	698	24290	6.0
SX-61-42	Lower submember of He 8	14260	2197	636	24832	3726	234	45890	6.0
SX-24–39	Shan 1 Member	4717.5	4785.5	846.7	15906.4	2867.4	315.4	29438.9	6.9

### Temperature–pressure sensitivity of multicomponent gas solubility

Based on the thermodynamic model described above, the solubility responses of CH$_4$, He, N$_2$, and CO$_2$ were calculated over a broad range of reservoir temperatures and pressures. The calculations were performed under fixed formation-water chemistry, with salinity represented by ionic strength. The model uses PR-EOS fugacity, van’t Hoff temperature correction, Poynting pressure correction, and Setschenow salting-out correction, consistent with the computational workflow used for the solubility simulator.

**Fig. 20:**
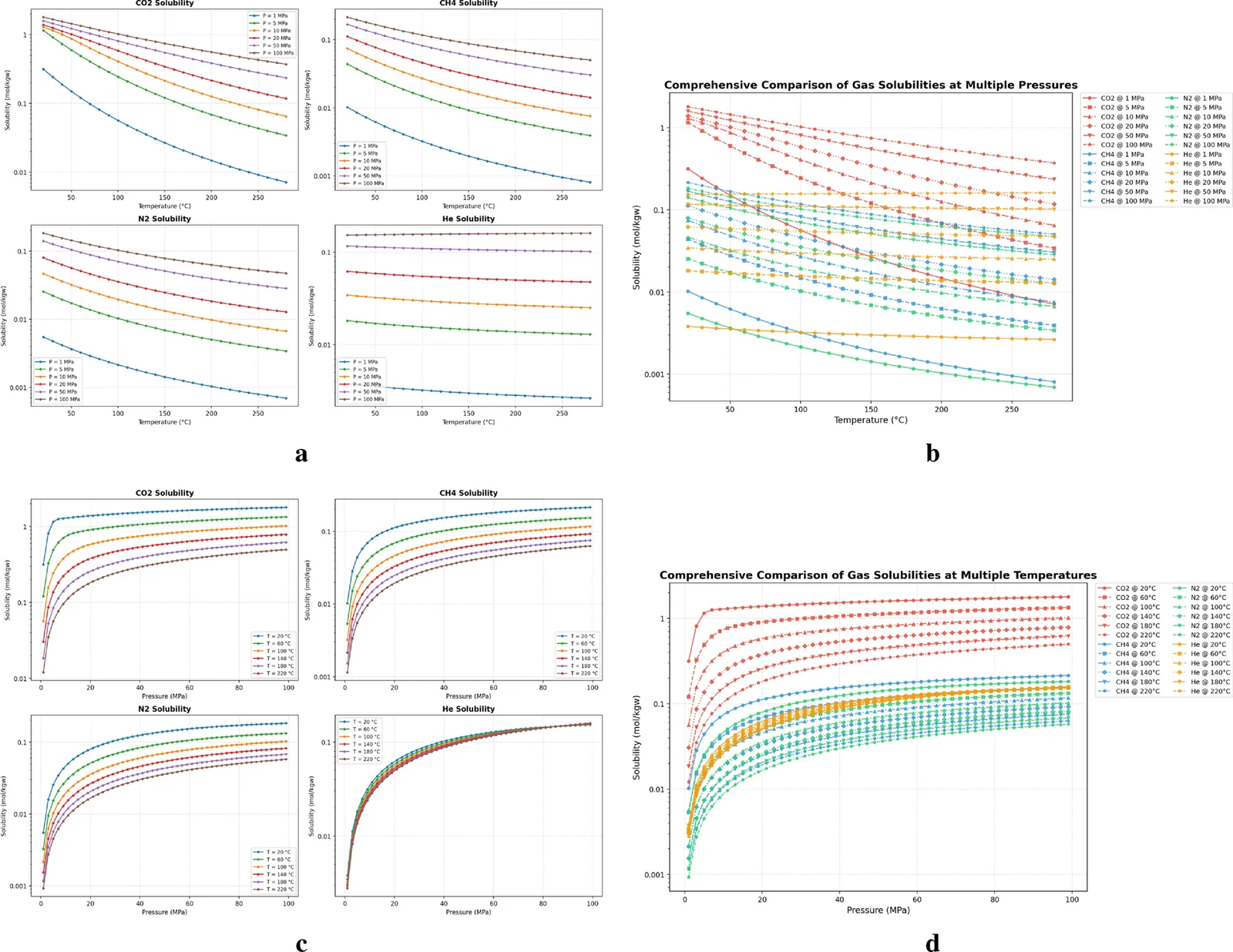
Temperature and pressure sensitivity of multicomponent gas solubility in formation water. (**a**) Single-component temperature-sensitivity curves for He, N$_2$, CH$_4$, and CO$_2$ under fixed-pressure cases. (**b**) Combined temperature-sensitivity comparison at multiple pressures. (**c**) Single-component pressure-sensitivity curves under fixed-temperature cases. (**d**) Combined pressure-sensitivity comparison at multiple temperatures. CO$_2$ shows the highest solubility, followed by CH$_4$, N$_2$, and He. The persistently low solubility of He indicates its strong preference for the gas phase, providing the thermodynamic basis for methane-driven helium stripping under deep reservoir conditions.

The temperature sensitivity curves in Fig. 20(a) and (b) show that gas solubility generally decreases with increasing temperature. This trend is consistent with the exothermic nature of gas dissolution and is expressed through the temperature-dependent solubility-form Henry coefficient. CO$_2$ exhibits the highest solubility because of its stronger interaction with water, whereas He remains at the lowest concentration level throughout the investigated temperature range. CH$_4$ and N$_2$ occupy intermediate positions. The weak temperature response of He indicates that its dissolved concentration is mainly limited by its intrinsically low water affinity rather than by thermal perturbation alone.

The pressure sensitivity curves in Fig. 20(c) and (d) show that solubility increases with pressure at low to moderate pressures because of enhanced gas fugacity. However, under high-pressure conditions, the response becomes progressively nonlinear due to real-gas behaviour and the Poynting correction. This nonlinearity is especially important for deep reservoirs, where pressure enhancement does not translate into a proportional increase in aqueous gas storage capacity.

The calculated solubility hierarchy is:

41 $$\begin{aligned} C_{\textrm{CO}_2} \gg C_{\textrm{CH}_4}> C_{\textrm{N}_2}> C_{\textrm{He}} \end{aligned}$$

This ranking reflects differences in molecular interaction with water, polarity, and real-gas behaviour. CO$_2$ is strongly soluble because of hydration and chemical affinity with water, whereas He has the weakest interaction with the aqueous phase. Therefore, even though formation water may act as a long-term helium carrier, its capacity to retain helium is limited.

### Thermodynamic convention and gas–water partition definition

The thermodynamic notation and numerical implementation were checked and unified in this study. The Henry coefficient used here is the solubility-form coefficient, $K_{H,i}^{\textrm{sol}}(T)$, with units of mol kg$_{\textrm{w}}^{-1}$ atm$^{-1}$. The equilibrium aqueous concentration, or solubility limit, is denoted consistently as $C_i^*(T,P,I)$:

42 $$\begin{aligned} C_i^*(T,P,I)=K_{H,i}^{\textrm{sol}}(T) f_i^{(\textrm{atm})}\Pi _i S_i, \end{aligned}$$

where $f_i=\phi _iP$ is the fugacity, $f_i^{(\textrm{atm})}=f_i/P_{\textrm{atm}}$ is the fugacity expressed in atm, $\Pi _i=\exp [-\bar{V}_i(P-P_0)/(RT)]$ is the Poynting correction, and $S_i=10^{-k_{s,i}I}$ is the Setschenow salting-out correction. The Peng–Robinson fugacity coefficient was calculated using the gas or supercritical root of the cubic equation, and the fugacity-coefficient equation follows the same sign convention as the numerical implementation.

**Fig. 21:**
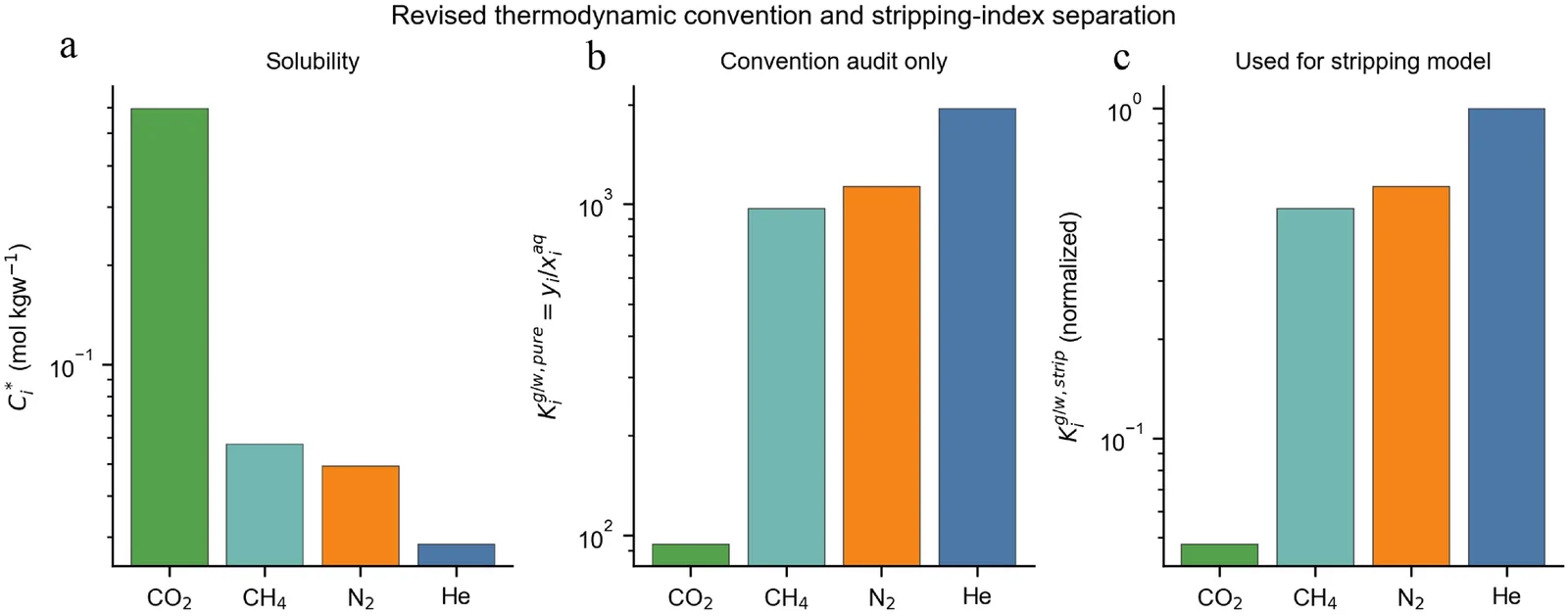
Thermodynamic convention and methane-stripping index separation. (**a**) Calculated single-gas equilibrium solubility $\boldsymbol{C}_{\boldsymbol{i}}^{*}$ under the representative condition of 113 $^\circ$ C, 32.5 MPa, and ionic strength of 0.65 molal. (**b**) Pure-gas reference gas-to-water coefficient $K_i^{\textrm{g}/\textrm{w,pure}}=\boldsymbol{y}_{\boldsymbol{i}}/\boldsymbol{x}_{\boldsymbol{i}}^{\boldsymbol{aq}}$, used only for convention auditing. (**c**) Normalized methane-stripping effective index $K_i^{\textrm{g}/\textrm{w,strip}}$, used in the 0D Rayleigh stripping model. A larger $K_i^{\textrm{g}/\textrm{w}}$ denotes stronger gas-phase preference. $K_i^{\textrm{g}/\textrm{w,strip}}$ is a parameterized stripping index rather than a fully calibrated multicomponent equilibrium constant.

The partition coefficient used in the stripping analysis is defined consistently in the gas-to-water direction:

43 $$\begin{aligned} K_i^{\textrm{g}/\textrm{w}}=\frac{y_i}{x_i^{\textrm{aq}}}, \end{aligned}$$

where $y_i$ is the gas-phase mole fraction and $x_i^{\textrm{aq}}$ is the aqueous mole fraction. Thus, a larger $K_i^{\textrm{g}/\textrm{w}}$ indicates stronger gas-phase preference and faster aqueous depletion during methane stripping. The reciprocal water-to-gas coefficient,

44 $$\begin{aligned} K_i^{\textrm{w}/\textrm{g}}=\frac{x_i^{\textrm{aq}}}{y_i}=\frac{1}{K_i^{\textrm{g}/\textrm{w}}}, \end{aligned}$$

was used only for direction checking and was not used in the stripping equations (Fig. 21).

To avoid mixing different thermodynamic conventions, two gas-to-water quantities were distinguished. The pure-gas reference coefficient, $K_i^{\textrm{g}/\textrm{w,pure}}$, was derived from the single-gas equilibrium solubility and was used only to audit the gas/water direction. The 0D methane-stripping model used a normalized effective stripping index, $K_i^{\textrm{g}/\textrm{w,strip}}$, to represent the relative depletion tendency of trace gases during methane flushing. Both quantities use the same gas-to-water direction, but they do not have the same physical meaning: $K_i^{\textrm{g}/\textrm{w,pure}}$ is a single-gas reference coefficient, whereas $K_i^{\textrm{g}/\textrm{w,strip}}$ is a parameterized and normalized effective index for the Rayleigh-type stripping model. In the stripping model, CH$_4$ was treated as a buffered carrier gas, while He, N$_2$, and CO$_2$ were treated as passive trace gases subject to aqueous depletion.

The thermodynamic diagnostic shows the expected inverse relation between aqueous solubility and gas-to-water partition tendency under the representative condition of 113 $^\circ$ C, 32.5 MPa, and ionic strength of 0.65 molal. The calculated solubility follows $\textrm{CO}_2>\textrm{CH}_4>\textrm{N}_2>\textrm{He}$, whereas the pure-gas reference coefficient follows $K_{\textrm{He}}^{\textrm{g}/\textrm{w,pure}}>K_{\textrm{N}_2}^{\textrm{g}/\textrm{w,pure}}>K_{\textrm{CH}_4}^{\textrm{g}/\textrm{w,pure}}>K_{\textrm{CO}_2}^{\textrm{g}/\textrm{w,pure}}$. This is consistent with the adopted convention that low aqueous solubility corresponds to high gas-phase preference. The normalized methane-stripping index used in the 0D Rayleigh model follows the same trace-gas tendency, with He assigned the strongest stripping response.

**Fig. 22:**
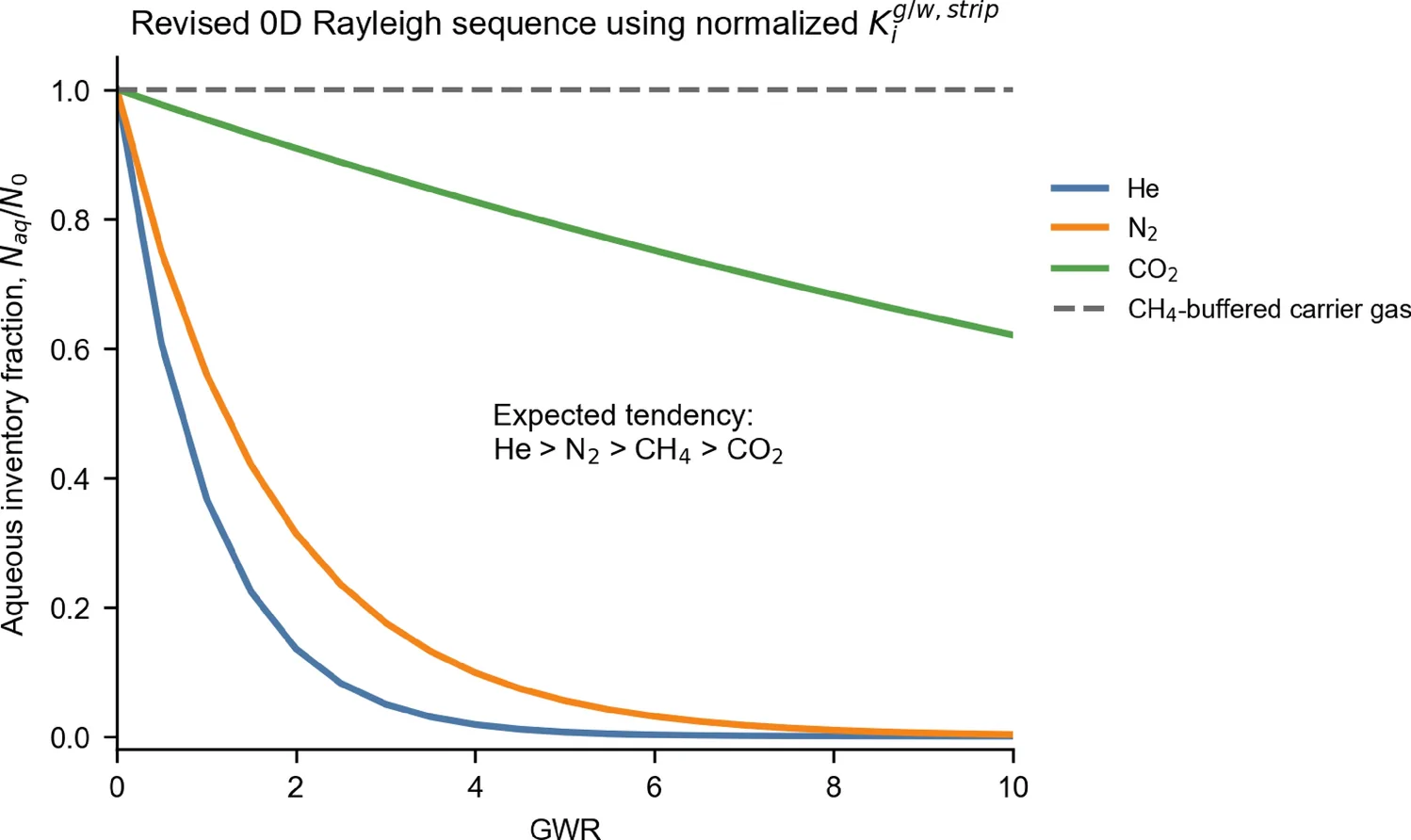
Revised 0D Rayleigh stripping sequence using normalized gas-to-water stripping indices. The aqueous inventory fraction $\boldsymbol{N}_{\boldsymbol{aq}}/\boldsymbol{N}_{\textbf{0}}$ decreases according to the normalized methane-stripping index $K_i^{\textrm{g}/\textrm{w,strip}}$. He shows the fastest depletion, followed by N$_2$ and CO$_2$. CH$_4$ is treated as a buffered carrier gas and is not passively depleted. The curves are intended to represent relative stripping tendency under methane flushing rather than field-scale recovery prediction.

The revised 0D sequence therefore shows the fastest aqueous depletion for He, followed by N$_2$ and CO$_2$, while CH$_4$ remains buffered as the carrier gas (Fig. 22).

### Spatial distribution of multiphase fluids and dissolved gases

The solubility analysis in Sections "Thermodynamic model of gas solubility under high-temperature and high-pressure conditions" and "Temperature–pressure sensitivity of multicomponent gas solubility" defines the thermodynamic upper limit of gas dissolution in formation water. However, in tight sandstone, dissolved gases are not uniformly distributed throughout the pore system. Their spatial occurrence is controlled by the topology of water-bearing pores, gas-filled pores, and nano-confined pore throats. Therefore, the reconstructed sub-voxel porosity field was further coupled with the gas solubility model to map the initial distribution of free gas, bound water, and dissolved gas components.

In this study, the continuous porosity field $\hat{\phi }(\textbf{x})$ obtained from Section "Sub-voxel pore structure reconstruction" was used as the spatial basis for phase partitioning. A porosity threshold $\phi _{\textrm{bound}}$ was introduced to distinguish bound-water-dominated regions from free-gas-accessible regions. Pores with $\hat{\phi }(\textbf{x}) \le \phi _{\textrm{bound}}$ were treated as bound-water regions, whereas pores with $\hat{\phi }(\textbf{x})> \phi _{\textrm{bound}}$ were assigned as free-gas-accessible pore space. In this study, $\phi _{\textrm{bound}} = 0.10$ was used as the threshold for water-locked nano-pore regions.

The local water saturation was expressed as:

45 $$\begin{aligned} S_w(\textbf{x})= {\left\{ \begin{array}{ll} 1, & \hat{\phi }(\textbf{x}) \le \phi _{\textrm{bound}} \\ S_{wr}, & \hat{\phi }(\textbf{x})> \phi _{\textrm{bound}} \end{array}\right. } \end{aligned}$$

where $\phi _{\textrm{bound}}=0.10$ is the bound-water porosity threshold and $S_{wr}=0.25$ is the residual water saturation assigned to gas-accessible pores. The corresponding local gas saturation is:

46 $$\begin{aligned} S_g(\textbf{x})=1-S_w(\textbf{x}) \end{aligned}$$

The local water-filled and gas-filled pore volumes can therefore be written as:

47 $$\begin{aligned} & V_w(\textbf{x})=\hat{\phi }(\textbf{x})S_w(\textbf{x})\Delta V \end{aligned}$$

48 $$\begin{aligned} & V_g(\textbf{x})=\hat{\phi }(\textbf{x})S_g(\textbf{x})\Delta V \end{aligned}$$

where *Δ V* is the voxel volume.

The thermodynamic solubility limit of gas component *i*, denoted as $C_i^{*}(T,P,I)$, was calculated using the multicomponent gas solubility model described in Section "Thermodynamic model of gas solubility under high-temperature and high-pressure conditions". The dissolved gas amount in each voxel was then mapped as:

49 $$\begin{aligned} n_{i}^{\textrm{aq}}(\textbf{x})=C_i^{*}(T,P,I)\rho _w V_w(\textbf{x}) \end{aligned}$$

where $n_{i}^{\textrm{aq}}(\textbf{x})$ is the amount of dissolved component *i*, $\rho _w$ is the formation-water density, and $V_w(\textbf{x})$ is the local water-filled pore volume. This formulation ensures that dissolved gas is restricted to water-bearing pore space, rather than being artificially distributed throughout the entire pore network.

For visualization, the dissolved gas distribution was expressed in logarithmic form:

50 $$\begin{aligned} C_{i,\log }(x)=\log _{10}\!\left[ n_{i}^{\textrm{aq}}(\textbf{x})+\varepsilon \right] \end{aligned}$$

where *\varepsilon* is a small constant introduced to avoid singularity in water-free regions.

**Fig. 23:**
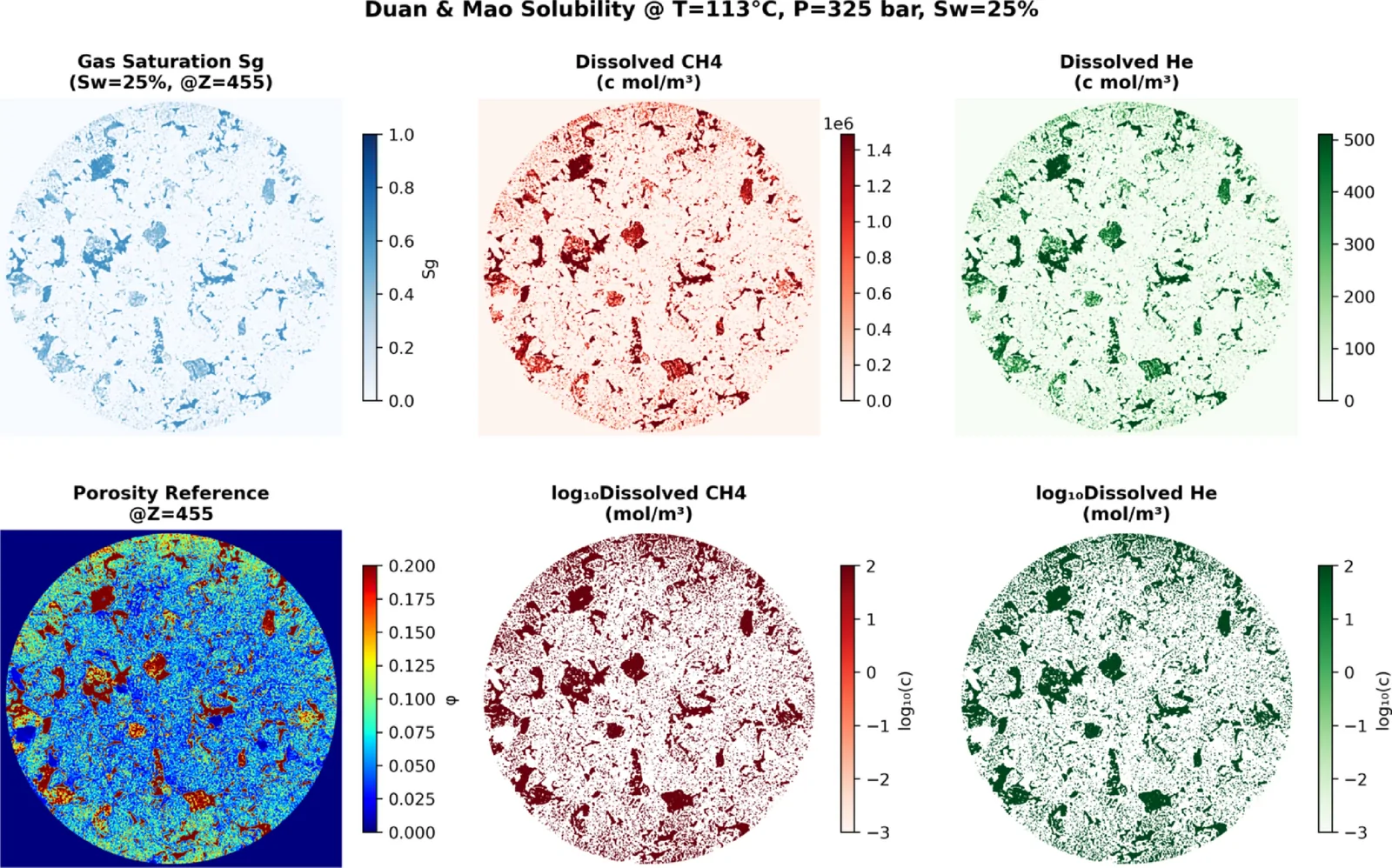
Spatial distribution of multiphase fluids and dissolved gas components constrained by the reconstructed porosity field and thermodynamic solubility model. Free gas mainly occupies large connected pores and microfractures, whereas bound water is retained along pore margins and within low-porosity nano-pore regions. Dissolved gas distributions are mapped only within water-bearing pore space according to the solubility limit $C_i^{*}(T,P,I)$. Helium shows the lowest dissolved abundance and is spatially dispersed within thin bound-water regions, providing the initial condition for methane-driven stripping.

As shown in Fig. 23, free gas mainly occupies large connected pores and microfractures, whereas bound water is concentrated along pore margins and within low-porosity nano-pore regions. The dissolved gas components are therefore spatially controlled by the geometry of water-bearing pore space. Among the investigated gases, helium shows the lowest dissolved amount because of its intrinsically low solubility. Its distribution is highly dispersed and restricted to thin bound-water regions, indicating that helium is initially stored in a low-concentration and spatially fragmented aqueous state.

This spatial configuration has an important implication for methane-driven stripping. Static pressure depletion alone is unlikely to efficiently recover helium from isolated bound-water regions. Instead, effective helium release requires continuous gas–water contact, interfacial mass transfer, and convective flushing by the mobile methane phase. The mapped distribution therefore provides the initial condition for the subsequent dynamic stripping model.

### Macroscopic evolution of supercritical methane stripping: a 0D box model

Continuous charging of supercritical methane into water-bearing tight sandstone does not only provide a mobile gas phase, but also disturbs the gas–water equilibrium of dissolved trace gases. Based on the solubility constraints and partition convention established in Sections "Thermodynamic model of gas solubility under high-temperature and high-pressure conditions"–"Thermodynamic convention and gas–water partition definition", a zero-dimensional Rayleigh-type box model was developed to describe the macroscopic stripping of dissolved helium by methane. The model treats the formation water as a well-mixed reservoir and assumes that each increment of injected methane reaches local gas–water equilibrium before leaving the system with the stripped gas components. The symbol $K_i^{\textrm{g}/\textrm{w,strip}}$ is used throughout this section to distinguish the normalized stripping index from the pure-gas reference coefficient $K_i^{\textrm{g}/\textrm{w,pure}}$.

The stripping efficiency of gas component *i* is controlled by a normalized effective gas-to-water stripping index, defined consistently with the gas-to-water convention in Equation (43):

51 $$\begin{aligned} K_i^{\textrm{g}/\textrm{w,strip}}=\frac{y_{i,n}^{\textrm{eq}}}{x_{i,n}^{\textrm{aq}}} \end{aligned}$$

where $y_{i,n}^{\textrm{eq}}$ is the equilibrium mole fraction of component *i* in the outgoing methane-rich gas increment at stripping step *n*, and $x_{i,n}^{\textrm{aq}}$ is the corresponding aqueous mole fraction. The superscript “strip” indicates that this quantity is the coefficient used in the Rayleigh-type methane-stripping update; it retains the gas-to-water direction of $K_i^{\textrm{g}/\textrm{w,pure}}$ but represents an effective methane-rich gas–water exchange index rather than a pure-component Henry reference. All stripping equations below use $K_i^{\textrm{g}/\textrm{w,strip}}$, not the reciprocal water-to-gas form. A larger $K_i^{\textrm{g}/\textrm{w,strip}}$ indicates a stronger tendency for the component to partition into the gas phase and a faster aqueous depletion rate. Under the deep reservoir conditions considered in this study, helium has the strongest gas-phase preference because of its extremely low aqueous solubility, providing the thermodynamic basis for preferential methane-driven helium extraction.

For a trace component *i*, the initial dissolved amount in the aqueous phase is defined as:

52 $$\begin{aligned} N_{i,0}^{\textrm{aq}} = C_i^{*}(T,P,I)M_w \end{aligned}$$

where $C_i^{*}(T,P,I)$ is the equilibrium solubility obtained from the thermodynamic model, and $M_w$ is the mass of formation water. During each stripping step, an incremental gas-to-water ratio $\Delta \textrm{GWR}$ of methane is introduced. Here, $\Delta \textrm{GWR}=\Delta N_{\mathrm {CH_4}}^{\textrm{inj}}/n_w$ is a dimensionless molar gas-to-water ratio, where $n_w=M_w/M_{\mathrm {H_2O}}$ is the number of water moles. Thus, $K_i^{\textrm{g}/\textrm{w,strip}}\Delta \textrm{GWR}$ is dimensionless. The discrete update follows from mass conservation and local partition equilibrium,

53 $$\begin{aligned} N_{i,n-1}^{\textrm{aq}}=N_{i,n}^{\textrm{aq}}+N_{i,n}^{\textrm{gas}}, \qquad \frac{N_{i,n}^{\textrm{gas}}}{\Delta N_{\mathrm {CH_4}}^{\textrm{inj}}}=K_i^{\textrm{g}/\textrm{w,strip}}\frac{N_{i,n}^{\textrm{aq}}}{n_w} \end{aligned}$$

which gives the residual aqueous amount after the *n*-th step as:

54 $$\begin{aligned} N_{i,n}^{\textrm{aq}} = \frac{N_{i,n-1}^{\textrm{aq}}}{1 + K_i^{\textrm{g}/\textrm{w,strip}}\,\Delta \textrm{GWR}} \end{aligned}$$

and the amount stripped into the methane gas phase is:

55 $$\begin{aligned} N_{i,n}^{\textrm{gas}} = N_{i,n-1}^{\textrm{aq}} - N_{i,n}^{\textrm{aq}} \end{aligned}$$

For small stripping increments, the cumulative Rayleigh-type depletion can be approximated as:

56 $$\begin{aligned} N_i^{\textrm{aq}}(\textrm{GWR}) = N_{i,0}^{\textrm{aq}} \exp (-K_i^{\textrm{g}/\textrm{w,strip}}\,\textrm{GWR}) \end{aligned}$$

where $\textrm{GWR}$ is the cumulative dimensionless gas-to-water molar ratio. This expression shows that components with larger $K_i^{\textrm{g}/\textrm{w,strip}}$ are removed from the aqueous phase more rapidly.

Methane was treated differently from trace gases because it acts as the continuously supplied carrier phase. Instead of being depleted from the aqueous phase, dissolved CH$_4$ was maintained near its equilibrium saturation concentration:

57 $$\begin{aligned} C_{\mathrm {CH_4}}^{\textrm{aq}} \approx C_{\mathrm {CH_4}}^{*}(T,P,I) \end{aligned}$$

This buffer condition reflects continuous replenishment by the injected methane phase and prevents methane from being treated as a passive trace component.

The gas-phase composition of the stripped product at each step was calculated as:

58 $$\begin{aligned} y_{i,n}^{\textrm{out}} = \frac{N_{i,n}^{\textrm{gas}}}{\Delta N_{\mathrm {CH_4}}^{\textrm{inj}} + \sum _j N_{j,n}^{\textrm{gas}}} \end{aligned}$$

where $\Delta N_{\mathrm {CH_4}}^{\textrm{inj}}$ is the amount of methane injected during the current step, and *j* includes the stripped trace components. This formulation links aqueous depletion to the transient enrichment of trace gases in the produced methane stream.

**Fig. 24:**
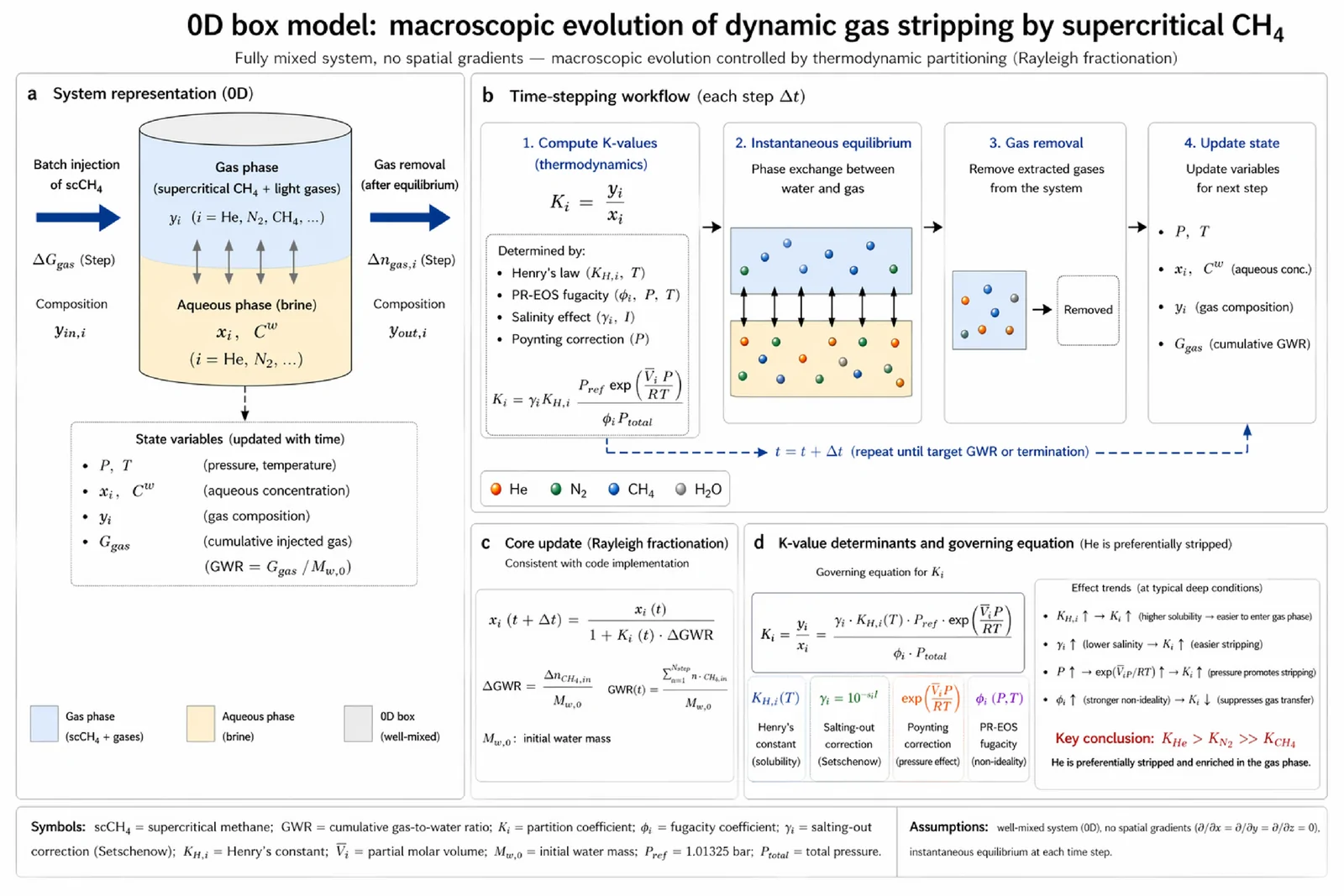
Evolution of aqueous gas concentrations during supercritical methane stripping in the 0D Rayleigh box model. The cumulative gas-to-water ratio controls the progressive removal of dissolved trace gases from formation water. Helium shows the fastest depletion because of its strong gas-phase preference, whereas N$_2$ and CO$_2$ are removed more slowly. Dissolved CH$_4$ remains approximately constant because methane is continuously supplied as the carrier gas and maintained near saturation.

As shown in Fig. 24, dissolved helium decreases most rapidly with increasing gas-to-water ratio, whereas N$_2$ and CO$_2$ show slower depletion. CH$_4$ remains nearly constant because of the imposed saturation-buffer condition. The steep decline of aqueous helium indicates that early methane charging is highly effective in extracting helium from formation water.

**Fig. 25:**
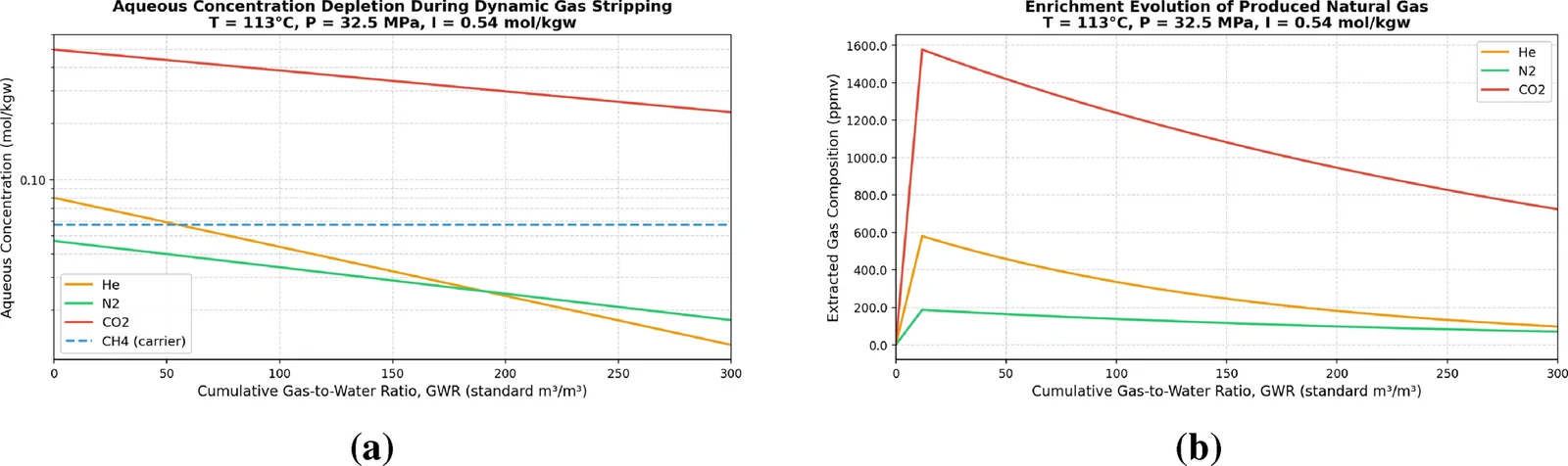
Macroscopic evolution of multicomponent gas stripping during supercritical methane charging in the 0D box model. (**a**) Depletion of aqueous gas concentrations during methane stripping, shown on a logarithmic scale. CH$_4$ remains nearly constant because it is continuously replenished by the injected methane phase and maintained close to its aqueous saturation limit. In contrast, He, N$_2$, and CO$_2$ show component-dependent depletion controlled by their effective gas-to-water stripping indices, $K_i^{\textrm{g}/\textrm{w,strip}}=y_{i,n}^{\textrm{eq}}/x_{i,n}^{\textrm{aq}}$. (**b**) Evolution of trace-gas enrichment in the produced gas phase. He shows an early enrichment peak because of its high effective gas-to-water stripping index and rapid extraction from formation water. Continued methane injection subsequently dilutes the produced gas as the aqueous He pool becomes depleted, whereas N$_2$ and CO$_2$ display delayed enrichment behaviour.

As shown in Fig. 25(a), the aqueous concentrations of different gas components decrease at different rates during supercritical methane stripping. On the logarithmic scale, the depletion trajectories directly reflect differences in the effective gas-to-water stripping index, $K_i^{\textrm{g}/\textrm{w,strip}}=y_{i,n}^{\textrm{eq}}/x_{i,n}^{\textrm{aq}}$. CH$_4$ remains nearly constant throughout the process because it is continuously supplied by the injected methane phase and maintained close to its aqueous saturation concentration. Therefore, CH$_4$ acts as a buffered carrier gas rather than a passively depleted trace component.

The trace gases show clear component-dependent depletion. He exhibits the steepest decline and is rapidly removed from the aqueous phase at relatively low gas-to-water ratios, especially when GWR *< 100*. N$_2$ shows an intermediate depletion rate, whereas CO$_2$ is removed more slowly because of its stronger affinity for the aqueous phase. This differential stripping sequence indicates that early methane charging is particularly effective in mobilizing dissolved He from formation water.

The produced-gas composition in Fig. 25(b) further illustrates the component-dependent enrichment behaviour during stripping. He displays an early enrichment peak because it has the highest effective gas-to-water stripping index, $K_i^{\textrm{g}/\textrm{w,strip}}$, among the trace components considered. As a result, dissolved He is preferentially transferred into the first increments of methane-rich gas. After the aqueous He pool becomes depleted, continued injection of methane mainly dilutes the produced gas, leading to a rapid decline in He concentration after the peak.

By contrast, N$_2$ and CO$_2$ exhibit more delayed enrichment patterns and require larger cumulative gas-to-water ratios to be fully released from the aqueous phase. This delayed response reflects their lower effective gas-to-water stripping indices, $K_i^{\textrm{g}/\textrm{w,strip}}$, compared with He. The 0D box model therefore provides a macroscopic thermodynamic explanation for why methane charging can preferentially extract dissolved He before other components.

However, the box model assumes instantaneous mixing and equilibrium exchange between gas and water. It does not resolve pore-scale limitations associated with gas–water contact area, tortuous diffusion pathways, or isolated water-bearing nano-pores in tight sandstone. Therefore, the following section uses pore-scale lattice Boltzmann simulations based on the reconstructed digital core to examine whether this thermodynamic stripping tendency can be realized under spatially heterogeneous pore-network conditions.

## Pore-scale LBM simulation of supercritical methane-driven helium extraction

The thermodynamic and 0D box models in Section "Multicomponent gas solubility and macroscopic stripping kinetics under deep conditions" indicate that dissolved helium tends to transfer from formation water into the methane-rich gas phase. However, these models assume instantaneous mixing and cannot resolve the effects of pore-scale heterogeneity, capillary confinement, local gas–water contact, and diffusion-limited release from isolated water-bearing pores^30,62,69^. In tight sandstone, these pore-scale constraints are critical because the gas–water interface is spatially discontinuous and the transport pathway is strongly controlled by micro–nano pore geometry.

To examine whether the thermodynamic stripping tendency can be realized under realistic pore-scale constraints, a grayscale lattice Boltzmann model was constructed using the reconstructed sub-voxel porosity field as the computational domain^63,70^. The model simulates methane invasion, water displacement, and helium release in nine rotated two-dimensional slices extracted from different *z* positions of the reconstructed three-dimensional porosity volume. The porosity slices were smoothed and clipped to preserve continuous pore–solid transitions, allowing unresolved sub-voxel heterogeneity to influence fluid transport without imposing a binary pore–matrix boundary.

In this model, water and methane are treated as two interacting fluid phases using a Shan–Chen multiphase LBM formulation^71,72^, whereas helium is treated as a dissolved scalar transported by the local velocity field. Helium extraction is activated only in cells where methane and water coexist, representing interfacial transfer from the aqueous phase to the mobile methane phase. This design links the macroscopic stripping potential predicted in Section "Multicomponent gas solubility and macroscopic stripping kinetics under deep conditions" with the spatially heterogeneous pore-scale transport process controlled by the reconstructed tight sandstone structure. The resulting extraction dynamics therefore reflect coupled slip-enhanced migration, pore-scale accessibility, and diffusion-controlled tailing in tight pore networks^73–77^.

Compared with a conventional scalar-transport LBM, the present implementation introduces three additional constraints. First, a spatially heterogeneous helium relaxation-time field is calculated from the porosity-dependent pore radius and a Knudsen hindrance factor, representing Bosanquet-type diffusion limitation. This parameterization is used only for helium scalar diffusion in the 2D grayscale LBM domain and is distinct from the hydraulic radius used for permeability inversion in Section "Preferential gas-phase migration and slippage mechanisms in tight pore–throat systems". Second, the parameterized local transfer term increases with flow velocity, representing Sherwood-type convective mass-transfer enhancement during methane flushing. Third, a mass-conserving rescaling step is applied after helium collision to prevent artificial mass creation caused by positivity clipping. These treatments improve the physical consistency and numerical stability of long-term methane-driven helium extraction simulations.

### Coupled Shan–Chen LBM model for methane invasion and helium extraction

The pore-scale flow simulation was implemented using a D2Q9 lattice Boltzmann scheme with a BGK collision–streaming operator, following the standard lattice BGK formulation of Qian et al. and related LBM developments^32,57,58^. Two distribution functions, $f_i^w$ and $f_i^c$, were used to describe water and methane, respectively. Their evolution follows the BGK collision–streaming equation:

59 $$\begin{aligned} f_i^{m}(\textbf{x}+e_i\Delta t,t+\Delta t)=f_i^{m}(\textbf{x},t)-\frac{1}{\tau _{m}}\left[ f_i^{m}(\textbf{x},t)-f_i^{m,\textrm{eq}}(\textbf{x},t)\right] , \qquad m\in \{w,c\} \end{aligned}$$

where *m* denotes water or methane, $e_i$ is the discrete lattice velocity, $\tau _{m}$ is the relaxation time, and $f_i^{m,\textrm{eq}}$ is the equilibrium distribution function. The equilibrium distribution is expressed as:

60 $$\begin{aligned} f_i^{m,\textrm{eq}}=w_i\rho _{m}\left[ 1+3(e_i\cdot u)+\frac{9}{2}(e_i\cdot u)^2-\frac{3}{2}u^2\right] \end{aligned}$$

where $w_i$ is the lattice weight, $\rho _{m}$ is the phase density, and *u* is the mixture velocity. In the simulation, both water and methane relaxation times were set to 1.0 for numerical stability.

The interaction between methane and water was represented using a Shan–Chen pseudopotential force. The pseudopotential function was defined as:

61 $$\begin{aligned} \psi (\rho )=1-\exp (-\rho ) \end{aligned}$$

For phase *m*, the interaction force was written as:

62 $$\begin{aligned} F_{m}(\textbf{x})=-\Psi _{\textrm{sc}}\psi _{m}(\textbf{x})\,\phi (\textbf{x})\sum _i w_i\,\psi _{n}(\textbf{x}+e_i)\,e_i, \qquad m,n\in \{w,c\},\quad m\ne n \end{aligned}$$

where *w* and *c* denote water and methane, respectively. The porosity term allows the reconstructed grayscale pore structure to directly modulate local phase interaction and invasion behaviour. In the updated implementation, $\Psi _{\textrm{sc}}$ was not treated as an arbitrary constant. Instead, it was parameterized as:

63 $$\begin{aligned} \Psi _{\textrm{sc}}(T,P,S)=\textrm{clip}\left[ \Psi _{\textrm{sc},0}-a_T(T-20)-a_P P+a_S S,\,\Psi _{\textrm{sc},\min },\Psi _{\textrm{sc},\max }\right] \end{aligned}$$

where *T* is temperature in $^\circ \textrm{C}$, *P* is pressure in MPa, and *S* is salinity in wt%. The adopted parameters were $\Psi _{\textrm{sc},0}=5.2$, $a_T=0.006$, $a_P=0.015$, $a_S=0.04$, $\Psi _{\textrm{sc},\min }=3.6$, and $\Psi _{\textrm{sc},\max }=6.0$. This empirical mapping was used to represent the first-order decrease in gas–water interfacial resistance with increasing temperature and pressure, and the increase in interfacial resistance with increasing salinity. The clipping bounds keep $\Psi _{\textrm{sc}}$ within the Shan–Chen numerical stability interval.

To represent partially resolved pore boundaries, a porosity-dependent volume-bounce-back weight was introduced:

64 $$\begin{aligned} \omega _{\textrm{bb}}(\textbf{x})=1-[1-\phi (\textbf{x})]^3 \end{aligned}$$

In high-porosity regions, $\omega _{\textrm{bb}}$ approaches 1 and free streaming dominates. In low-porosity regions, the bounce-back contribution increases, representing stronger pore-wall restriction. This treatment allows the simulation to preserve the continuous nature of the sub-voxel porosity field.

Helium was represented by an independent scalar distribution function $g_i$. The local dissolved helium concentration is recovered as:

65 $$\begin{aligned} C_{\textrm{He}}(\textbf{x},t)=\sum _i g_i(\textbf{x},t) \end{aligned}$$

and its transport equation is:

66 $$\begin{aligned} g_i(\textbf{x}+e_i\Delta t,t+\Delta t)=g_i(\textbf{x},t)-\frac{1}{\tau _{\textrm{He}}(\textbf{x})}\left[ g_i(\textbf{x},t)-g_i^{\textrm{eq}}(\textbf{x},t)\right] \end{aligned}$$

with the scalar equilibrium distribution:

67 $$\begin{aligned} g_i^{\textrm{eq}}=w_i C_{\textrm{He}}\left[ 1+3(e_i\cdot u)\right] \end{aligned}$$

Because the gas-slippage analysis and the dynamic LBM stripping simulation serve different purposes, two pore-size proxies were used. The three-dimensional hydraulic radius $r_{\textrm{hyd}}$ was used for permeability inversion and apparent gas slippage, whereas the two-dimensional diffusion proxy $R_{\textrm{diff}}$ was used only to construct the heterogeneous helium relaxation-time field in the grayscale LBM model. Sensitivity tests on $\Delta x_{\textrm{LBM}}$ and $R_{\textrm{diff}}$ are required in future work to further quantify the uncertainty introduced by this approximation.

Accordingly, $\tau _{\textrm{He}}(\textbf{x})$ is the local helium relaxation time. Unlike a uniform-diffusivity scalar model, $\tau _{\textrm{He}}$ was defined as a spatially heterogeneous field controlled by local porosity and Knudsen diffusion limitation.

For the scalar helium-transport calculation in the two-dimensional grayscale LBM domain, the local diffusion length scale was approximated using a porosity-weighted pore-radius proxy:

68 $$\begin{aligned} R_{\textrm{diff}}(\textbf{x})=\Delta x_{\textrm{LBM}}\,\phi (\textbf{x}) \end{aligned}$$

Here, $R_{\textrm{diff}}$ is not used as the hydraulic pore–throat radius for permeability inversion. Instead, it represents an effective local diffusion length in the grayscale LBM slice, and $\Delta x_{\textrm{LBM}}$ denotes the lattice spacing of the two-dimensional grayscale LBM domain. The hydraulic radius $r_{\textrm{hyd}}$ defined in Section "Preferential gas-phase migration and slippage mechanisms in tight pore–throat systems" was used for three-dimensional pore–throat permeability and slip-flow analysis, whereas $R_{\textrm{diff}}$ was used only to parameterize Knudsen-limited helium diffusion in the two-dimensional scalar-transport simulation.

The Knudsen hindrance factor was introduced as a Bosanquet-type diffusion parameterization to account for pore-confinement-limited helium diffusion in the 2D grayscale LBM domain.

69 $$\begin{aligned} C_{\textrm{Kn}}(\textbf{x})=\frac{1}{1+\lambda /R_{\textrm{diff}}(\textbf{x})} \end{aligned}$$

where *λ* is the molecular collision length scale used for the He–water system. The effective diffusion coefficient was then obtained as:

70 $$\begin{aligned} D_{\textrm{eff}}(\textbf{x})=D_{\textrm{bulk}}\,C_{\textrm{Kn}}(\textbf{x}) \end{aligned}$$

This formulation should be interpreted as a numerically stable Bosanquet-type diffusion parameterization rather than a direct substitution for the hydraulic radius used in the permeability model. Diffusion remains close to bulk diffusion in larger pores but becomes strongly restricted in nano-confined regions. This parameterization is used only for helium scalar diffusion in the 2D grayscale LBM domain and is distinct from the hydraulic radius used for permeability inversion in Section "Preferential gas-phase migration and slippage mechanisms in tight pore–throat systems".

All LBM evolution equations were solved in lattice units. Unless otherwise stated, one numerical iteration corresponds to $\Delta t_{\textrm{LBM}}=1$. The physical time scale $\Delta t_{\textrm{phys}}$ was used only when converting the effective diffusion coefficient to lattice diffusivity:

71 $$\begin{aligned} D_{\textrm{LBM}}(\textbf{x})=D_{\textrm{eff}}(\textbf{x})\frac{\Delta t_{\textrm{phys}}}{\Delta x_{\textrm{LBM}}^2}, \qquad \tau _{\textrm{He}}(\textbf{x})=0.5+3D_{\textrm{LBM}}(\textbf{x}) \end{aligned}$$

Therefore, $k_{\textrm{loc}}$ is interpreted as a dimensionless helium removal fraction per LBM step, rather than a continuous-time rate constant. With numerical bounds imposed to maintain stability, solid or nearly solid cells were assigned a neutral relaxation time and excluded from helium transport.

Helium extraction was activated only in cells satisfying:

72 $$\begin{aligned} \phi (\textbf{x})>10^{-3},\qquad \rho _w(\textbf{x})>0.15,\qquad \rho _c(\textbf{x})>0.7 \end{aligned}$$

The local extraction coefficient used in the LBM extraction model is an implementation-specific parameterization rather than an independently calibrated Sherwood-number correlation. It is used to represent the combined effects of diffusion-limited helium supply and velocity-enhanced interfacial stripping within the pore-scale analogue model. Therefore, the extraction-rate results should be interpreted as parameterized pore-scale simulations, and their sensitivity to empirical parameters is evaluated separately. In these cells, the parameterized local transfer term was defined as:

73 $$\begin{aligned} k_{\textrm{loc}}(\textbf{x},t)=k_{\textrm{diff}}(\textbf{x})+\chi _u|\textbf{u}(\textbf{x},t)| \end{aligned}$$

where $k_{\textrm{loc}}$ is the dimensionless per-step helium removal fraction, $k_{\textrm{diff}}$ represents the diffusion-limited contribution, $|\textbf{u}|$ is the local methane-flow velocity in lattice units, and $\chi _u$ is the velocity-enhancement coefficient. The symbol $\chi _u$ is used to avoid confusion with $\beta _t$, which is reserved for the throat-constriction factor in the hydraulic-radius model. Unless otherwise stated, the reference values $k_{\textrm{diff}}(\textbf{x})=0.05$ and $\chi _u=3.0$ were used.

The amount of helium removed from the aqueous phase during one LBM step was:

74 $$\begin{aligned} \Delta C_{\textrm{He}}^{\textrm{ext}}(\textbf{x},t)=k_{\textrm{loc}}(\textbf{x},t)C_{\textrm{He}}(\textbf{x},t) \end{aligned}$$

and the local concentration was updated as:

75 $$\begin{aligned} C_{\textrm{He}}(\textbf{x},t+\Delta t_{\textrm{LBM}})=C_{\textrm{He}}(\textbf{x},t)-\Delta C_{\textrm{He}}^{\textrm{ext}}(\textbf{x},t) \end{aligned}$$

with $$\Delta t_{\textrm{LBM}}=1$$.

The cumulative extracted helium mass was defined as the sum of two non-overlapping contributions:

76 $$\begin{aligned} N_{\textrm{ext}}(t)=N_{\textrm{sink}}^{\textrm{cum}}(t)+N_{\textrm{out}}^{\textrm{cum}}(t) \end{aligned}$$

where

77 $$\begin{aligned} N_{\textrm{sink}}^{\textrm{cum}}(t)=\sum _{m=1}^{t}\sum _{x\in \Omega _{\textrm{int}}}\Delta C_{\textrm{He}}^{\textrm{ext}}(x,m) \end{aligned}$$

and

78 $$\begin{aligned} N_{\textrm{out}}^{\textrm{cum}}(t)=\sum _{m=1}^{t}\sum _{x\in \Omega _{\textrm{out}}}\sum _i g_i(x,m) \end{aligned}$$

Here, $\Omega _{\textrm{int}}$ denotes CH$_4$–water coexistence cells, and $\Omega _{\textrm{out}}$ denotes the outlet boundary region. The outlet contribution was counted only once because the helium distributions in outlet cells were cleared immediately after being accumulated. The helium recovery factor was then calculated as:

79 $$\begin{aligned} R_{\textrm{He}}(t)=\frac{N_{\textrm{ext}}(t)}{N_{\textrm{He},0}}\times 100\% \end{aligned}$$

where $N_{\textrm{He},0}$ is the initial dissolved helium mass.

**Table 6:** Parameters used in the Shan–Chen LBM helium extraction simulation.

Symbol	Meaning	Value or range	Unit/note
$\tau _w$	Water relaxation time	1.0	lattice unit
$\tau _c$	Methane relaxation time	1.0	lattice unit
*T*	Reservoir temperature	113–120	$^\circ \textrm{C}$; representative range
*P*	Reservoir pressure	20–32.5	MPa; representative range
*S*	Salinity	2.7–5.0	wt%; representative range
$\Psi _{\textrm{sc},0}$	Reference Shan–Chen phase-separation strength	5.2	lattice unit
$a_T$	Temperature coefficient in $\Psi _{\textrm{sc}}(T,P,S)$	0.006	$^\circ \textrm{C}^{-1}$
$a_P$	Pressure coefficient in $\Psi _{\textrm{sc}}(T,P,S)$	0.015	MPa$^{-1}$
$a_S$	Salinity coefficient in $\Psi _{\textrm{sc}}(T,P,S)$	0.04	wt%$^{-1}$
$\Psi _{\textrm{sc},\min },\Psi _{\textrm{sc},\max }$	Stability bounds of $\Psi _{\textrm{sc}}$	3.6, 6.0	lattice unit
$\Delta x_{\textrm{LBM}}$	Physical grid size	10–500; reference 50	nm per pixel
$\Delta t_{\textrm{phys}}$	Physical time per LBM step	0.1–10; reference 1.0	ns
*λ*	He–water collision length scale	0.3	nm
$k_{\textrm{diff}}$	Baseline He removal fraction per step	0–0.20; reference 0.05	dimensionless per step
$\chi _u$	Velocity-enhancement coefficient	0–10; reference 3.0	lattice-unit scaling
$k_{\textrm{loc}}$	Local He removal fraction per step	*łe 1.0*	dimensionless per step; not a calibrated physical mass-transfer coefficient
$\rho _w^{\textrm{crit}}$	Water threshold for extraction activation	0.15	lattice density
$\rho _c^{\textrm{crit}}$	Methane threshold for extraction activation	0.70	lattice density
$\phi _{\min }$	Minimum porosity for active transport	$10^{-3}$	dimensionless
$S_{wr}$	Residual water saturation in gas-accessible pores	0.25	dimensionless

A key numerical challenge in scalar LBM simulations is that positivity clipping of $g_i$ can artificially create or remove mass. To avoid this problem, the updated model applies a mass-conserving positivity rescaling after the helium collision step. Negative scalar distributions are first removed:

80 $$\begin{aligned} g_i^{*}(\textbf{x},t)=\max \left[ g_i(\textbf{x},t),0\right] \end{aligned}$$

and the post-collision distribution is rescaled as:

81 $$\begin{aligned} g_i^{\textrm{new}}(\textbf{x},t)=g_i^{*}(\textbf{x},t)\frac{C_{\textrm{He}}(\textbf{x},t)}{\sum _i g_i^{*}(\textbf{x},t)+\varepsilon } \end{aligned}$$

where *\varepsilon* is a small numerical constant. This procedure preserves the local macroscopic helium concentration while preventing negative distributions and unphysical mass generation (Table 6).

The simulation outputs include methane invasion patterns, residual aqueous helium concentration, cumulative extracted helium, average helium concentration in water, the dynamic local transfer term, and helium recovery factor. These diagnostics allow the model to quantify how methane flushing, pore-scale heterogeneity, and diffusion-limited release jointly control the conversion of dissolved helium into mobile gas-phase helium under deep tight sandstone conditions.

**Fig. 26:**
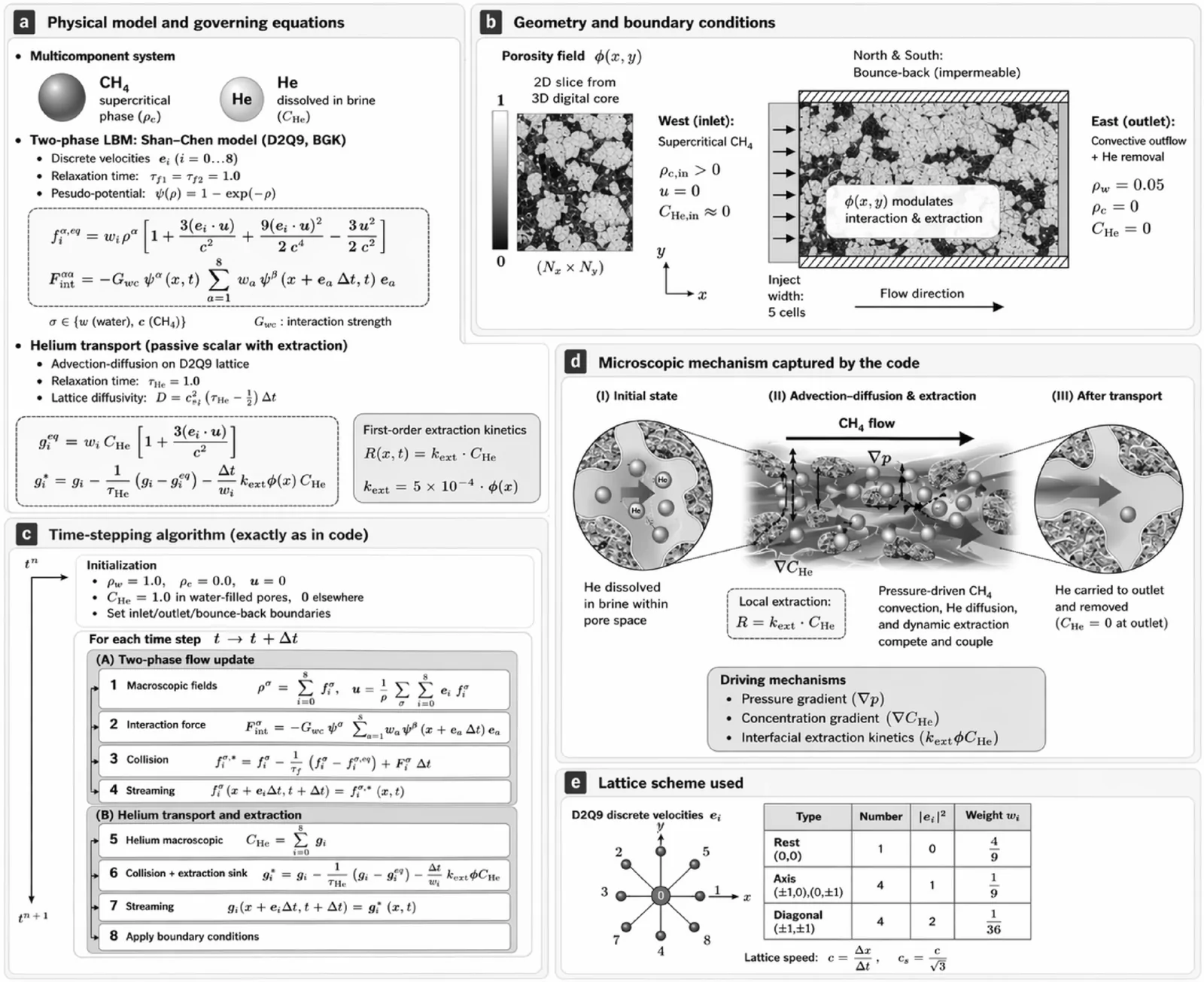
Lattice Boltzmann modelling workflow for supercritical CH$_4$-driven He extraction at the pore scale. The workflow integrates Shan–Chen multiphase LBM for CH$_4$–water flow, scalar advection–diffusion transport for dissolved He, porosity-controlled streaming and bounce-back, and a local per-step extraction term activated at CH$_4$–water coexistence regions. The reconstructed grayscale porosity field defines the computational domain and controls phase interaction, pore-wall restriction, and He transport. The model outputs phase distribution, He concentration, velocity field, cumulative extracted He, and recovery factor, which are used to evaluate methane-driven helium mobilization in tight sandstone. The local extraction term is a dimensionless per-step removal fraction used for mechanism-oriented simulation rather than an experimentally calibrated mass-transfer coefficient.

The complete numerical workflow is summarized in Fig. 26. Supercritical CH$_4$–water flow is first solved using the Shan–Chen LBM, followed by He advection–diffusion and local extraction. The reconstructed grayscale porosity field constrains phase transport, pore-wall restriction, and He diffusion. He extraction is activated only in CH$_4$–water coexistence regions through a per-step removal amount, $\Delta C_{\textrm{He}}^{\textrm{ext}}=k_{\textrm{loc}}C_{\textrm{He}}$. The outputs include phase distribution, He concentration, velocity field, cumulative extracted He, and recovery factor.

### Parameterized local transfer coefficient and Sherwood–Péclet diagnostic analysis

**Fig. 27:**
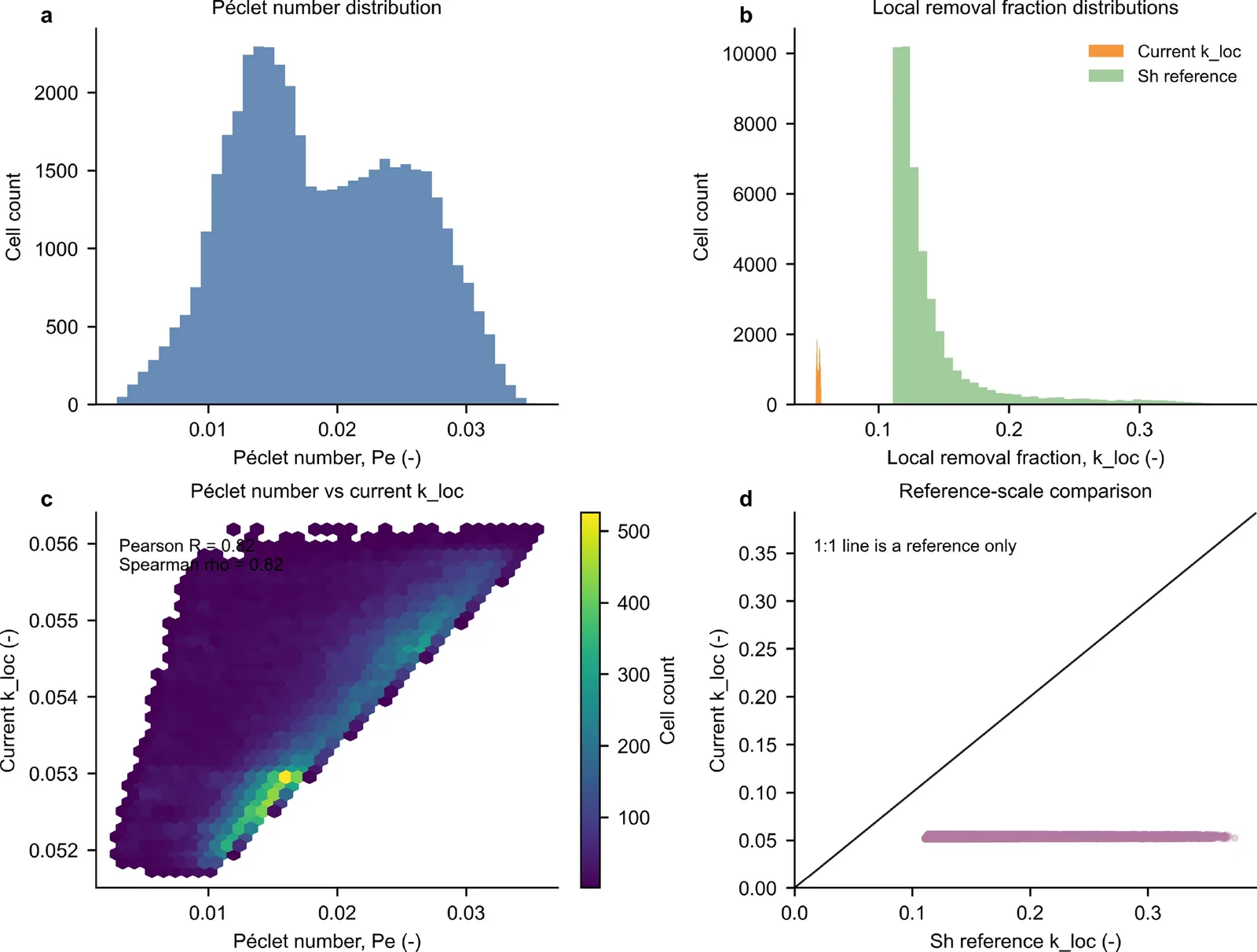
Sherwood–Péclet diagnostic analysis of the parameterized local removal fraction. (**a**) Distribution of the local Péclet number in gas–water contact regions. (**b**) Comparison between the current lattice-normalized $k_{\textrm{loc}}$ and the Sherwood-based reference removal fraction. (**c**) Relationship between $\textrm{Pe}$ and the current $k_{\textrm{loc}}$. The positive Pearson and Spearman correlations indicate that the parameterized $k_{\textrm{loc}}$ follows the local velocity/Péclet trend. (**d**) Reference-scale comparison between the current $k_{\textrm{loc}}$ and the Sherwood-based reference. The 1:1 line is shown only as a reference. The current $k_{\textrm{loc}}$ is a dimensionless per-step helium removal fraction in lattice units and is not calibrated to the Sherwood-based reference magnitude.

To clarify the physical meaning and uncertainty of the local extraction term, $k_{\textrm{loc}}$ is defined here as a dimensionless per-step helium removal fraction in lattice units, rather than as an experimentally calibrated physical mass-transfer coefficient. It is used as a parameterized local transfer term activated in CH$_4$–water coexistence cells, with $k_{\textrm{loc}}(\textbf{x},t)=k_{\textrm{diff}}(\textbf{x})+\chi _u|\textbf{u}(\textbf{x},t)|$, where $k_{\textrm{diff}}$ represents the baseline diffusive removal fraction and $\chi _u|\textbf{u}|$ represents velocity-enhanced transfer during methane flushing. To relate this simplified form to established convective mass-transfer concepts, a Sherwood–Péclet diagnostic analysis was added for gas–water contact cells (Fig. 27). The local Péclet number was calculated as $\textrm{Pe}=|\textbf{u}|L_c/D_{\textrm{eff}}$, and a Sherwood-type reference form was used only as a reference-scale diagnostic, not as a calibration target. Across the analysed slices, the current $k_{\textrm{loc}}$ shows a positive correlation with $\textrm{Pe}$ (Pearson *R=0.82*, Spearman *ρ =0.82*), indicating consistency with the local velocity/Péclet trend, although its magnitude remains lower than the Sherwood-based reference value.

**Fig. 28:**
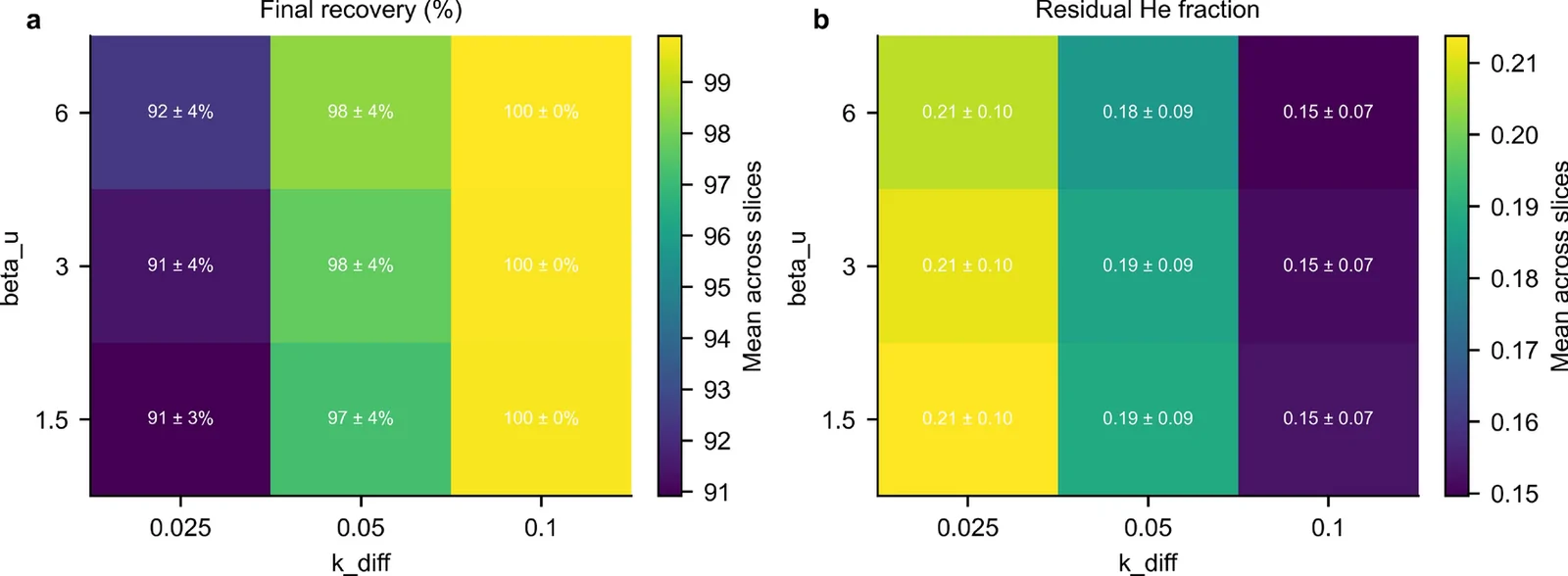
Sensitivity screening of the parameterized local transfer term. (**a**) Mean final recovery and (**b**) residual He fraction for different $k_{\textrm{diff}}$ and $\chi _u$ combinations. Values are reported as mean ± SD across slices. Increasing $k_{\textrm{diff}}$ increases final recovery and decreases residual He retention, whereas $\chi _u$ has a secondary effect within the tested range. The results are interpreted as a sensitivity screening of the parameterized transfer term rather than a full experimental calibration.

A sensitivity screening for $k_{\textrm{diff}}$ and $\chi _u$ further shows that increasing $k_{\textrm{diff}}$ from 0.025 to 0.10 increases the mean final recovery from approximately 91–92% to nearly 100% and decreases the residual He fraction from approximately 0.21 to 0.15, whereas the CH$_4$ contact-area fraction remains nearly unchanged at approximately *0.36± 0.03* (Fig. 28). Therefore, the LBM extraction results are interpreted as parameterized, mechanism-oriented pore-scale simulations, and the absolute extraction rate should be treated cautiously in the absence of direct pore-scale experimental calibration.

For clarity and reproducibility, Table 7 summarizes the correspondence between mathematical symbols and code variables used in the LBM diagnostics.

**Table 7:** Compact LBM nomenclature table. All LBM variables are either dimensional input parameters or lattice-normalized variables. The local removal fraction $k_{\textrm{loc}}$ is a dimensionless per-step helium removal fraction clipped to [0, 1], not a calibrated physical mass-transfer coefficient.

Symbol	Code variable	Meaning	Unit/normalization
$\rho _w$	rho_w	Water pseudo-density	lattice-normalized
$\rho _{\mathrm {CH_4}}$	rho_c	Methane pseudo-density	lattice-normalized
$\textbf{u}$	ux_f, uy_f	Local velocity	lattice velocity
$c_{\textrm{He}}$	c_he	Helium concentration scalar	normalized concentration
$\tau _{\textrm{He}}$	tau_he_field	He relaxation time	lattice time
$D_{\textrm{eff}}$	D_eff_lattice	Effective diffusivity	lattice cell$^2$ per step
$k_{\textrm{diff}}$	k_diff	Baseline local removal fraction	dimensionless per step
$\chi _u$	chi_u	Velocity-enhancement coefficient	per lattice velocity
$k_{\textrm{loc}}$	k_field	Local He removal fraction	dimensionless per step
$M_{\textrm{ext}}$	extracted_he_field	Extracted He inventory	normalized mass
*R*	recovery_factor	Recovery factor	%
$\textrm{Pe}$	Pe	Péclet number	dimensionless
$\textrm{Sh}$	Sh	Sherwood number	dimensionless

### Pore-scale flow-field evolution during dynamic methane stripping

After the thermodynamic boundary conditions were introduced into the LBM framework, supercritical CH$_4$ invasion and helium extraction exhibited strongly heterogeneous pore-scale dynamics. The displacement process was not characterized by piston-like advance. Instead, the methane front preferentially migrated through high-porosity connected channels and larger pore throats, producing localized fingering and channelized flow. This behaviour reflects the combined effects of low gas–water interfacial resistance, continuous methane injection, and the strong geometrical heterogeneity of the reconstructed tight sandstone pore network.

These results indicate a two-stage pattern for methane-driven helium extraction in the tested tight-sandstone analogue. The early stage is dominated by preferential methane breakthrough and rapid interfacial stripping along connected flow paths. The late stage is controlled by slow depletion of residual helium from isolated water-bearing pores. This transition explains why macroscopic recovery curves tend to rise rapidly at first and then gradually approach a plateau.

**Fig. 29:**
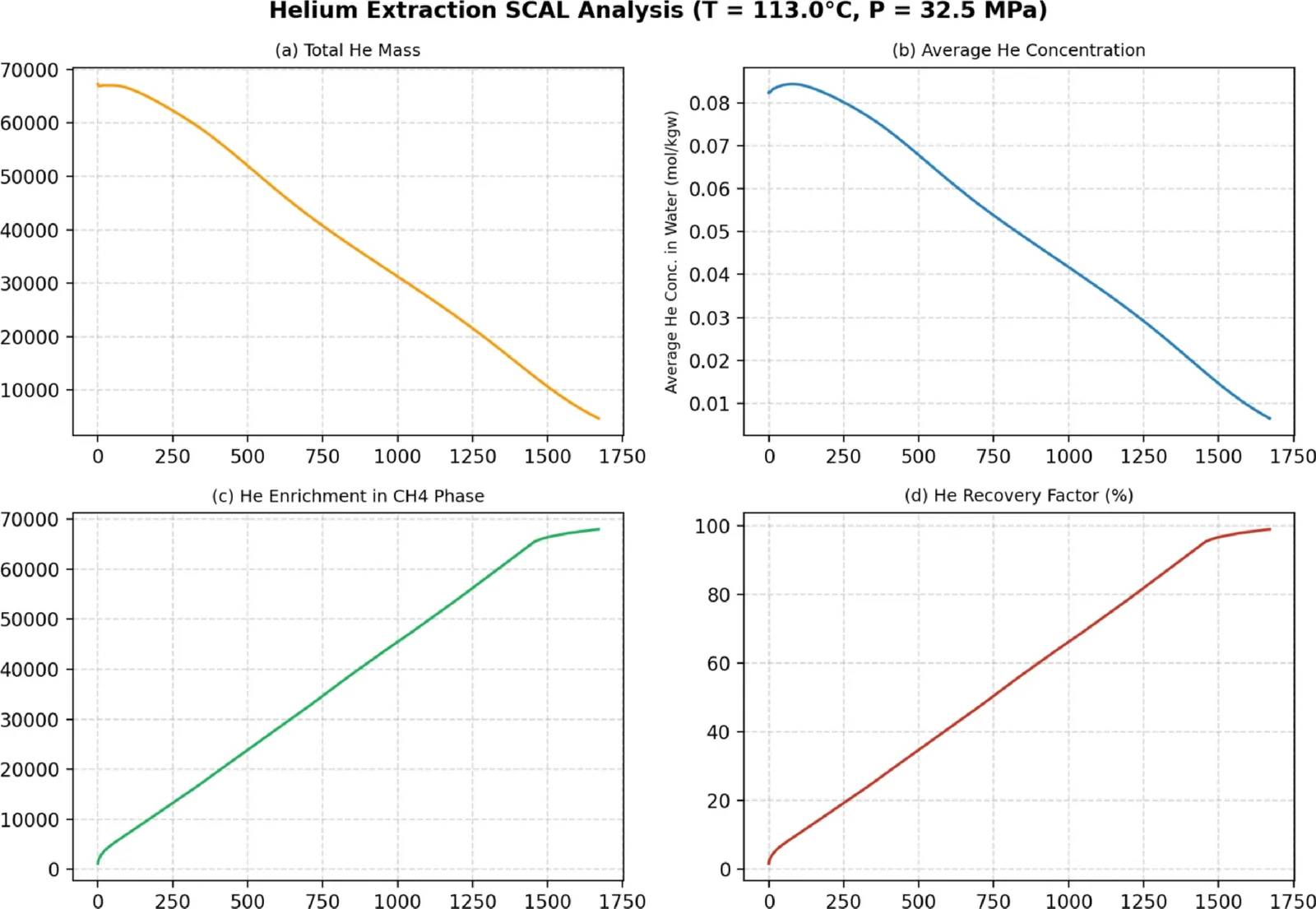
Integrated pore-scale flow-field and kinetic curves during supercritical CH$_4$-driven He extraction. (**a**) Total aqueous He mass during CH$_4$ injection. (**b**) Average aqueous He concentration. (**c**) Cumulative He enrichment in the CH$_4$ phase. (**d**) He recovery factor. Together, these panels show that the methane phase preferentially migrates through connected high-porosity channels, while dissolved He is progressively removed from water-bearing regions through coupled advection, diffusion, and local interfacial extraction.

**Fig. 30:**
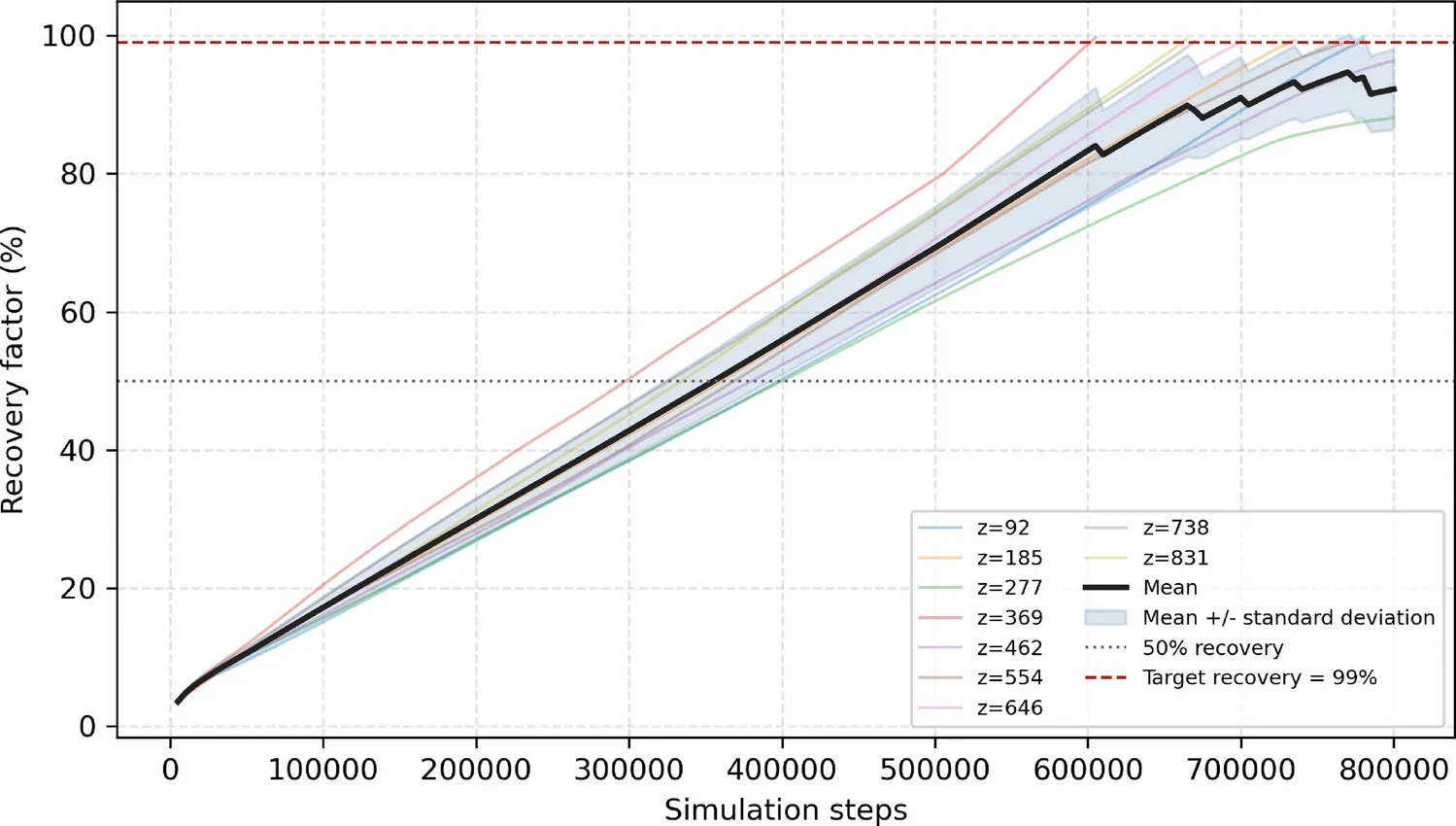
Multi-slice extraction metrics for the nine rotated 2D LBM slices. The curves summarize slice-to-slice variability in He recovery during CH$_4$-driven extraction. The mean trend and uncertainty band indicate that the extraction behaviour is reproducible across slices and is not based on a single favourable slice.

The multi-slice extraction metrics in Fig. 30 summarize slice-to-slice variability in the recovery trajectories.

The coupled flow and extraction behaviour is summarized in Fig. 29. In the early stage, methane rapidly forms preferential flow paths through the main pore network. These pathways increase the gas–water contact area and activate local extraction in cells where CH$_4$ and water coexist. Along the margins of the main methane channels, local velocity gradients enhance interfacial mass transfer, leading to rapid removal of dissolved helium from the adjacent water phase. By contrast, helium retained in low-connectivity pores, microfractures, and dead-end regions is released more slowly because transport is mainly controlled by diffusion rather than convective flushing.

**Fig. 31:**
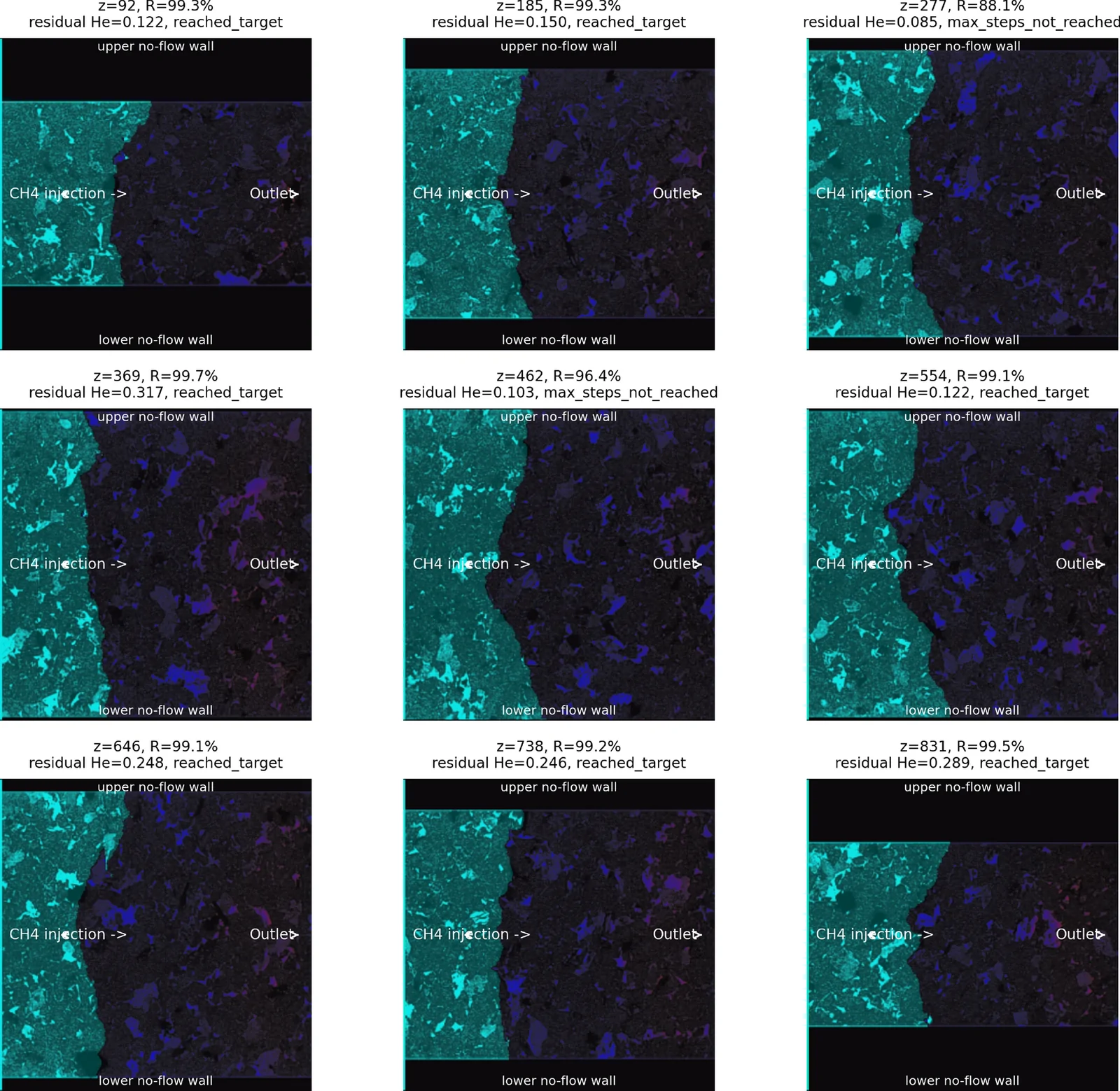
Multi-slice final states of CH$_4$-driven He extraction in rotated 2D LBM simulations. Nine slices were extracted from different *z* positions of the reconstructed 3D porosity volume and rotated clockwise by 90$^\circ$ to align the pore structure with the left-to-right CH$_4$ injection configuration. Cyan indicates CH$_4$-rich regions, blue–purple indicates residual aqueous He, and grey–black indicates the rock matrix or no-flow boundaries. The panels show that CH$_4$ invasion and He depletion are reproducible across different slices but vary in front geometry, residual He distribution, and recovery status because of slice-scale pore heterogeneity. These results are used as a multi-slice 2D representativeness check and do not constitute a full 3D LBM simulation.

The spatial evolution of the LBM animation is further illustrated in Fig. 31, where representative snapshots are shown at helium recovery factors of 20%, 40%, 60%, and 80%. At 20% recovery, methane begins to penetrate the major flow channels and the high-He regions remain widely distributed in water-bearing pores. At 40% recovery, the methane front expands laterally and helium depletion becomes concentrated along gas–water interfaces. At 60% recovery, most connected pore regions have been swept by methane, while residual helium is mainly preserved in isolated or weakly connected water-filled domains. At 80% recovery, the remaining helium is restricted to small residual pockets and low-velocity regions, indicating that the late stage of extraction is controlled by diffusion-limited release rather than channelized advection.

### Extraction kinetics and helium recovery evaluation

The extraction kinetics were evaluated using the diagnostic variables defined in Section "Coupled Shan–Chen LBM model for methane invasion and helium extraction", including residual aqueous He mass, average aqueous He concentration, cumulative extracted He, and He recovery factor. These variables were tracked throughout the simulation to quantify the conversion of dissolved He in water-bearing pores into mobile He carried by the CH$_4$ phase.

As shown in Fig. 29, the total aqueous He mass decreases continuously during CH$_4$ injection, whereas cumulative He enrichment in the CH$_4$ phase increases. The recovery curve exhibits a rapid-rise followed by slow-approach pattern. The initial rapid increase corresponds to CH$_4$ breakthrough through connected pore channels, where convective flushing and large gas–water contact area promote efficient He removal, as also illustrated by the early depletion patterns in Figure 31.

At higher recovery levels, the slope of the recovery curve decreases, indicating that easily accessible He has already been removed from connected pores. The remaining He is mainly stored in isolated water films, dead-end pores, and low-velocity microfractures. In these regions, CH$_4$ contact is limited and He release is controlled by diffusion-limited transport. Therefore, late-stage recovery cannot be explained by thermodynamic partitioning alone; it is strongly regulated by pore-network connectivity and local mass-transfer limitation.

The LBM results provide a pore-scale explanation for the difference between the idealized 0D box model and the more heterogeneous extraction process represented by the analogue tight-sandstone slices. The 0D model predicts thermodynamic favourability for methane-driven helium stripping, whereas the LBM simulation indicates that recovery is controlled by spatial heterogeneity, preferential flow, and diffusion-limited residual helium. In the analogue simulations, high modelled recovery therefore requires not only favourable gas–water partitioning, but also sufficient CH$_4$ access to water-bearing nano-pore networks.

## Discussion

Methane-driven helium mobilization in the tested tight-sandstone analogue is controlled by the coupling between thermodynamic partitioning and pore-scale accessibility^2–4,8,10–14^. The thermodynamic calculations indicate that helium has very low aqueous solubility and a strong gas-phase preference under the representative high-temperature, high-pressure, and high-salinity conditions used in this study^33,34,37–39^. The 0D stripping model further suggests that methane charging can generate a transient early-stage helium enrichment in the gas phase. However, this thermodynamic tendency can be realized only where methane physically contacts helium-bearing formation water. In tight sandstone, water may occur as thin films, isolated clusters, or dead-end pore fillings, so gas–water contact area, pore connectivity, and diffusion pathways become as important as equilibrium partitioning^24,29–32^. The mechanism inferred here is therefore not simply that methane acts as a passive carrier gas, but that methane behaves as a stripping phase only when it invades accessible water-bearing pore regions^33,34^.

The pore-scale simulations provide a mechanism-oriented interpretation of how this transfer may proceed within a heterogeneous tight-sandstone pore network. The reconstructed effective porosity field preserves continuous micro–nano-scale heterogeneity that would be partly lost in a purely binary pore model^21–25,78^, and the LBM simulations show a two-stage extraction pattern. Helium is first removed rapidly along connected methane-invasion pathways, where convective flushing and local gas–water coexistence promote interfacial transfer^18,29^. After these accessible regions are depleted, the release becomes slower and is controlled by diffusion-limited replenishment from weakly connected pores and residual water-bearing domains^30,74,77^. The He–CH$_4$ apparent-permeability contrast also supports a relative mobility advantage of helium in nano-confined throats, but this advantage is strongly pressure dependent and is substantially suppressed under representative deep-reservoir pressure^73–76^. Thus, the simulated enrichment behaviour reflects the combined effects of gas–water partitioning, methane accessibility, pressure-dependent slip-flow contrast, and late-stage diffusion tailing.

Several limitations constrain the transferability of these results. First, the pore geometry was obtained from the Wilcox Tight Gas Sandstone digital-rock dataset rather than from the Ninggu 3 target interval in the Ordos Basin. The Ordos data constrain depth, temperature, pressure, and formation-water salinity, whereas the Wilcox digital rock provides only an analogue tight-sandstone pore network^23,24,27^. Second, the extraction simulations were performed on multiple two-dimensional slices, which improves representativeness relative to a single slice but still cannot fully reproduce three-dimensional connectivity, isolated pore clusters, or tortuous gas–water interfaces^24,29,71,72^. Third, the grayscale-derived effective porosity field, Bosanquet-type diffusion limitation, and Sherwood-type local transfer enhancement remain parameterized approximations that require further calibration^32,76,77^. Therefore, the simulated extraction rate, depletion pattern, and length scale should be regarded as analogue pore-scale results rather than field-scale predictions for the Ordos Basin. Future work should apply the same set of calculations to target-interval Ordos digital cores and combine micro-CT, FIB-SEM, mineralogical mapping, and laboratory gas–water displacement or diffusion experiments to evaluate reservoir-specific transferability^23,24,27^.

## Conclusions

We examined methane-driven helium extraction from formation water under representative deep tight-reservoir conditions using a mechanism-oriented pore-scale modelling workflow. The Ordos Basin data defined temperature, pressure, and salinity constraints, whereas the Wilcox Tight Gas Sandstone digital rock was used as an analogue pore geometry rather than as a direct sample from the Ninggu 3 reservoir^2,3,23,24,27^.

Thermodynamic calculations indicate that helium has low aqueous solubility and a strong gas-phase partitioning tendency under the tested high-temperature, high-pressure, and high-salinity conditions^33,34,38,39^. The 0D methane-stripping model suggests that dissolved helium can be preferentially transferred into methane-rich gas during the early stage of methane charging, followed by dilution and depletion as the aqueous helium pool is progressively exhausted.

In the analogue pore-scale simulations, helium release is spatially heterogeneous and concentrated near methane–water contact regions^18,29^. Methane preferentially invades connected high-porosity pathways, producing rapid early extraction, whereas helium remaining in weakly connected pores or residual water-bearing domains is released more slowly by diffusion-limited replenishment^30,74,77^. Helium shows a relative apparent-permeability advantage over methane in nano-confined throats, but this contrast is pressure dependent and is strongly suppressed under representative deep-reservoir pressure^73–75^.

The results should be interpreted as analogue pore-scale evidence for methane-driven helium mobilization, not as direct reservoir-scale prediction. Quantitative extraction efficiency, depletion length scale, and field-scale enrichment potential remain uncertain because the simulations use an analogue digital rock, slice-based pore-scale calculations, grayscale-derived effective porosity, and parameterized local mass-transfer terms^23,24,27,32,71,72,76,77^. Target-interval Ordos digital cores and laboratory gas–water displacement or diffusion experiments are required before reservoir-specific conclusions can be drawn.

## Supplementary Information

Below is the link to the electronic supplementary material.

Supplementary Information 1.

Supplementary Information 2.

## Data Availability

The Wilcox Tight Gas Sandstone digital-rock dataset used as the analogue pore geometry is publicly available from the Digital Porous Media Portal and is cited in the manuscript. Reservoir temperature–pressure constraints, formation-water chemistry data, and Ordos tight-sandstone petrophysical comparison data were compiled from the literature sources cited in the Methods section and in Table 2. The processed datasets, simulation outputs, plotting data, parameter files, and analysis codes generated in this study have been deposited in Zenodo under 10.5281/zenodo.20605863. The deposited files include the reconstructed effective porosity fields or representative slice data, pore-throat radius distributions, Knudsen-number distributions, apparent-permeability and He–CH$_4$ mobility-contrast data, thermodynamic solubility and gas–water partitioning calculations, 0D methane-stripping outputs, multi-slice LBM extraction metrics, final-state maps, and the source data used to generate the main and supplementary figures. A README file in the repository describes the relationship between each dataset and the corresponding figure or table in the manuscript. The codes used for grayscale weak-label generation, CNN-based porosity reconstruction, pore-throat transport-parameter calculation, thermodynamic gas–water partitioning, methane-stripping calculation, LBM-based pore-scale extraction simulation, sensitivity analysis, and figure generation are available in the same repository, together with input-parameter files and a Python environment file. The original third-party Wilcox micro-CT volume is not redistributed in the repository because it is available from the Digital Porous Media Portal; users should obtain the original volume from that source and use the provided scripts and parameter files to reproduce the processed inputs used in this study.
