# Supplementary material for: Pore-scale modelling of methane-driven helium extraction in deep tight sandstones
Source: Sci Rep. 2026 Aug 25;16:26678. doi: 10.1038/s41598-026-60903-1 (PMC13507092; doi:10.1038/s41598-026-60903-1)
Supplement: Supplementary file 1 [file 41598_2026_60903_MOESM2_ESM.pdf]

Supplementary Information

Pore-scale modelling of methane-driven helium extraction in deep tight sandstones

Supplementary Data 1: Supplementary_Data_multislice.xlsx

Contents

Section	Contents
Supplementary Note S1 Geological boundary conditions	Geological logs, pressure-temperature constraints, and boundary-condition table
Supplementary Note S2 CNN reconstruction benchmark	CNN benchmark and patch-overlap consistency
Supplementary Note S3 Parameter sensitivity analysis	Parameter, Knudsen/slip, thermodynamic, and figure-clarity checks
Supplementary Note S4 Multi-slice extraction validation	Multi-slice extraction validation and Supplementary Data 1 reference
Supplementary Note S5 Local transfer coefficient and Sherwood-Péclet diagnostics	Local transfer coefficient, Sherwood-Péclet terms, and LBM nomenclature
Supplementary Tables	5 formal table entries
Supplementary Data file summary	Contents of Supplementary_Data_multislice.xlsx

Supplementary Note S1 Geological boundary conditions

The geological boundary conditions are defined from the selected well-log interval and the burial-depth trends for reservoir temperature and pressure. These constraints provide the stratigraphic and pressure-temperature setting used for the pore-scale calculations.

Supplementary Figure S1. Geological boundary conditions for the selected reservoir interval.

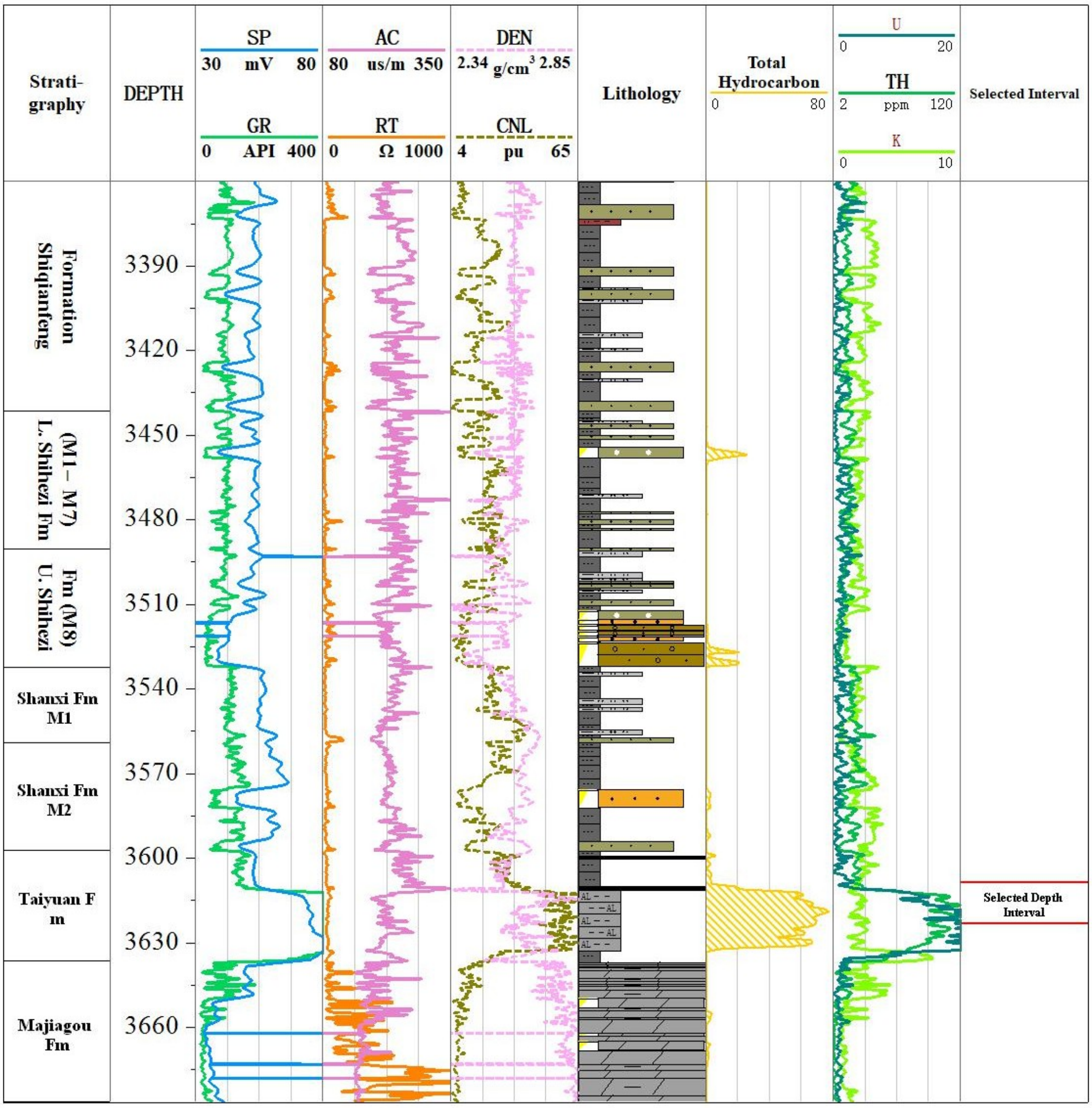

Composite well-log panel showing stratigraphy, depth, spontaneous potential, gamma ray, acoustic, resistivity, density, compensated neutron log, lithology, total hydrocarbon response, uranium, thorium and potassium logs, and the selected depth interval used to constrain the geological setting of the pore-scale modelling.

Supplementary Figure S2. Depth-dependent reservoir temperature and pressure constraints.

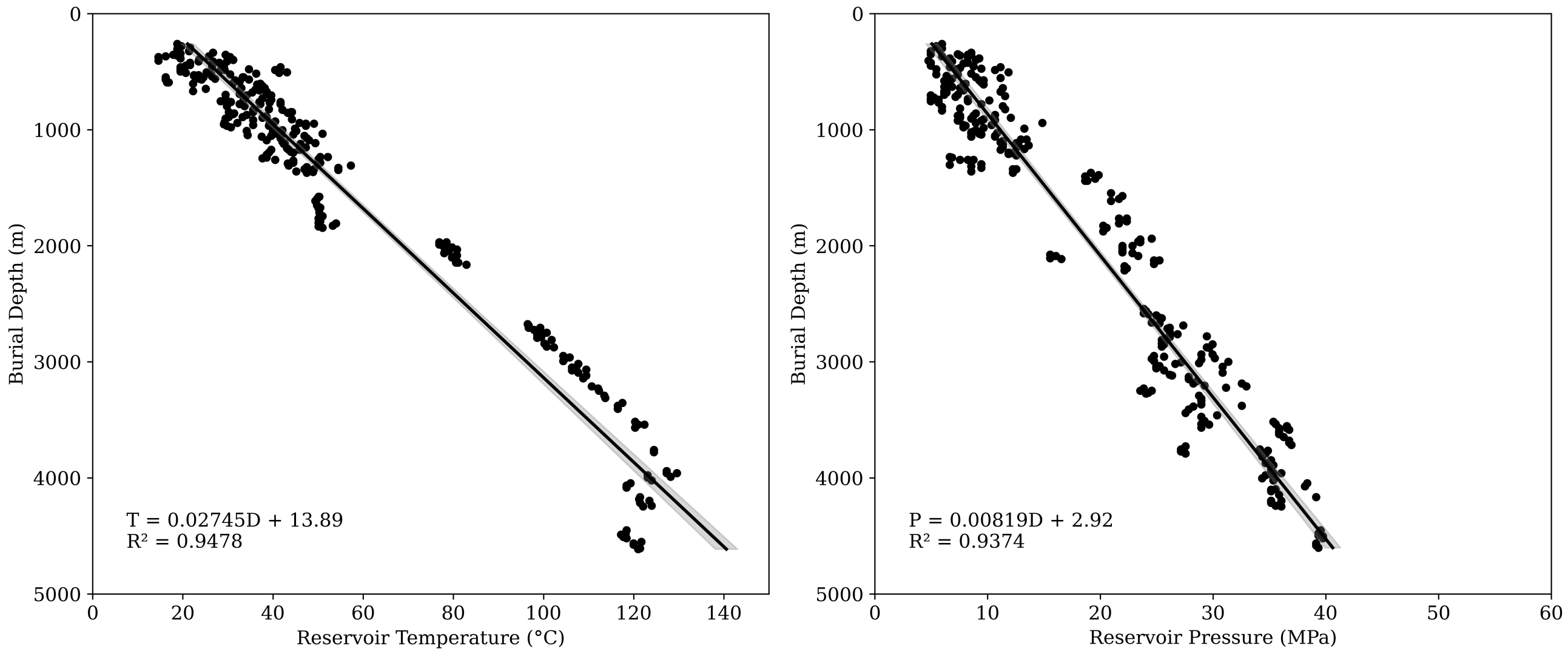

Black points show compiled burial-depth observations, and black lines show the fitted temperature-depth and pressure-depth trends. These trends set the pressure-temperature range used for the extraction calculations.

Supplementary Note S2 CNN reconstruction benchmark

The CNN reconstruction was checked against independent hold-out slices and a porosity-matched threshold baseline. The threshold case is used as a conventional comparison, while the continuous CNN field is used for the pore-scale calculations.

The patch-overlap configuration used in the revised manuscript is a 96 x 96 x 96 voxel patch, 16 voxel overlap, and 80 voxel stride for full-volume sliding-window inference.

Supplementary Figure S3. CNN reconstruction hold-out slice examples.

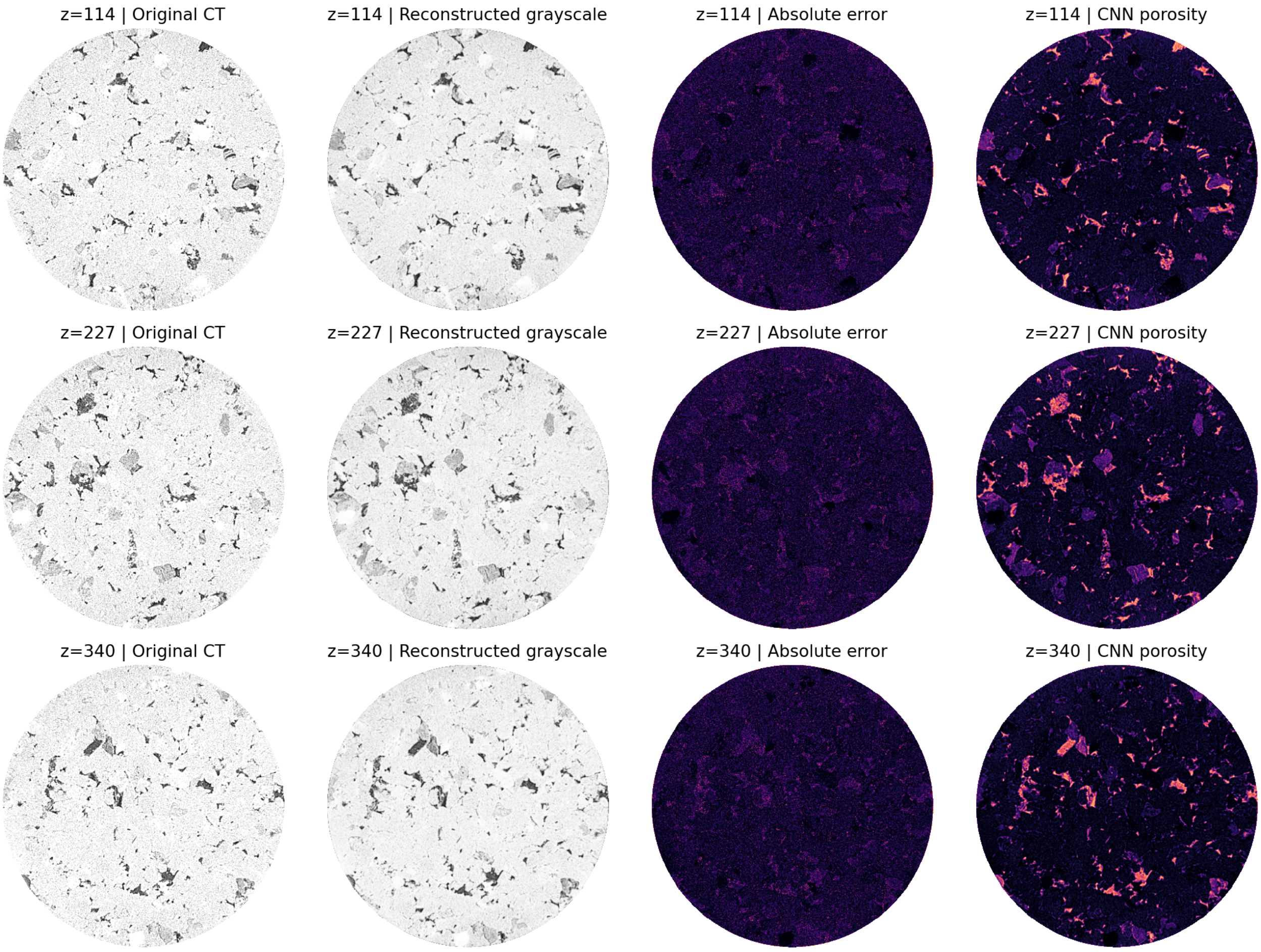

Representative hold-out slices used to assess visual and structural plausibility of the CNN-based porosity reconstruction. The panels compare image-derived pore features and reconstructed porosity fields at independent slice positions.

Supplementary Figure S4. Knudsen-number distribution comparison between threshold and CNN reconstructions.

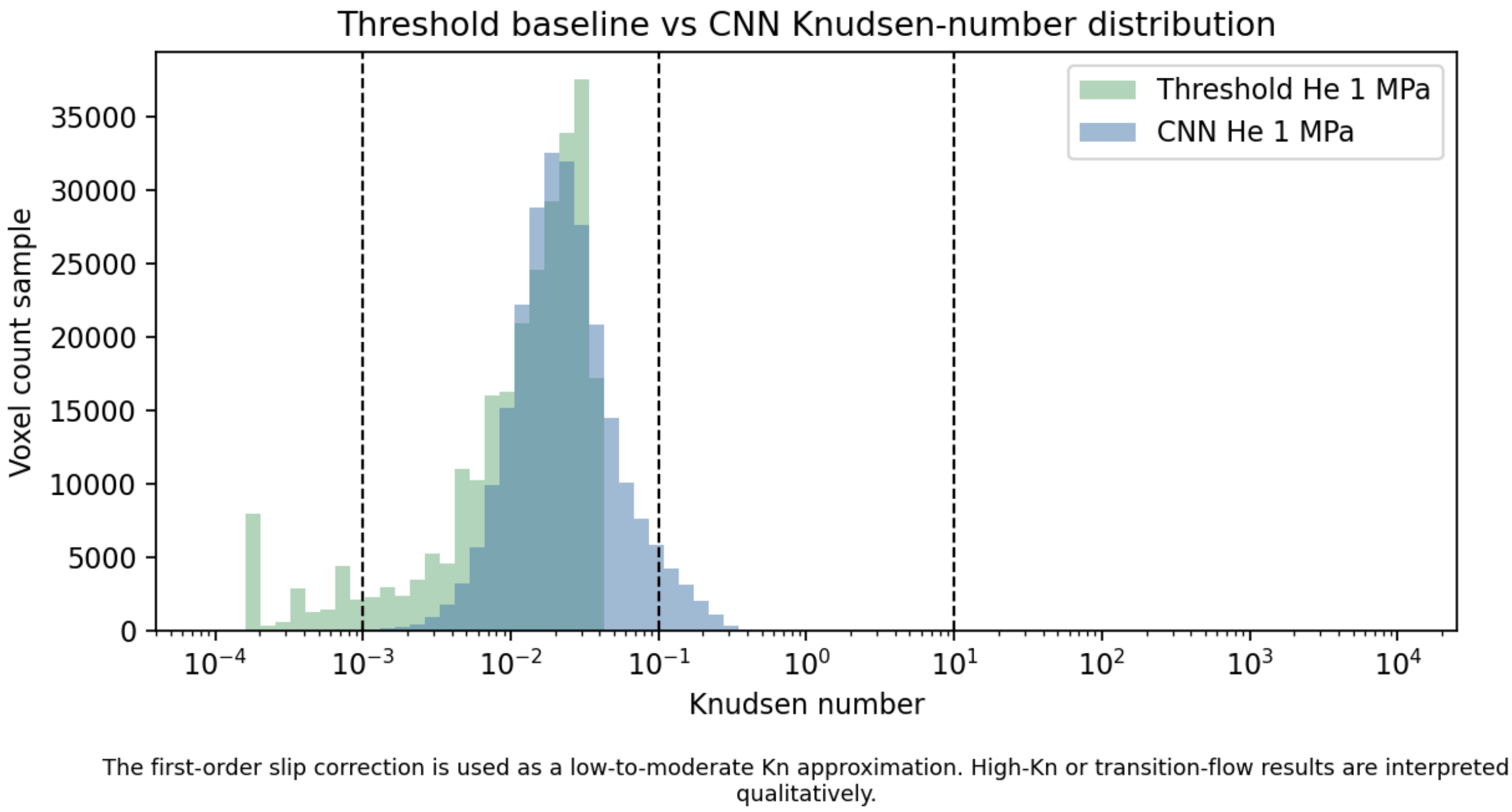

He Knudsen-number distributions are shown for the porosity-matched threshold baseline and the CNN reconstruction. The two curves indicate how the reconstruction choice affects the inferred flow-regime distribution.

Supplementary Figure S5. Permeability proxy benchmark for threshold and CNN reconstructions.

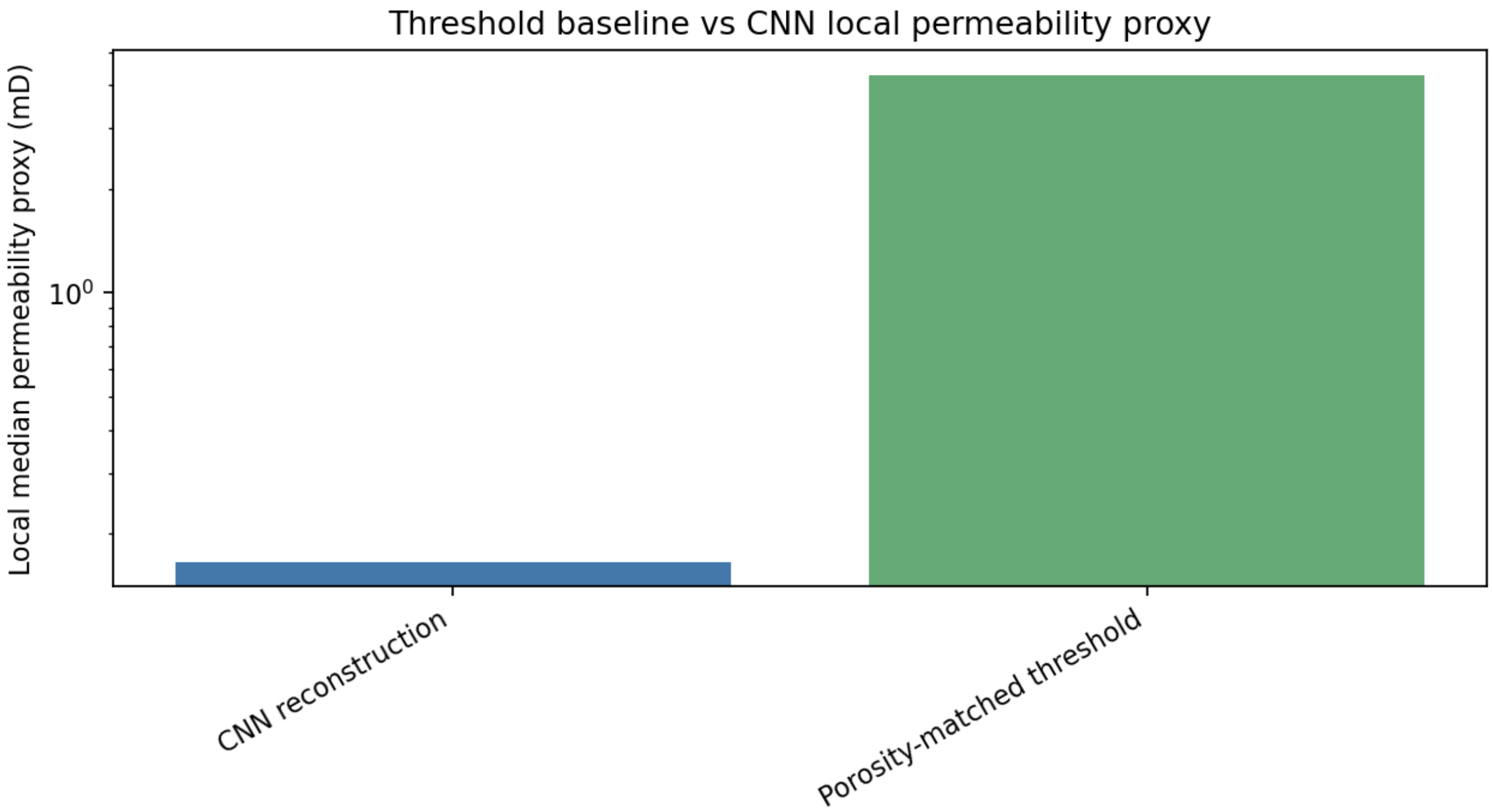

Voxel-scale permeability proxies are used only for relative comparison and should not be interpreted as direct core-scale permeability predictions.

Permeability-proxy metrics from threshold segmentation and CNN reconstruction show how binary thresholding can distort pore-throat connectivity and flow-capacity estimates relative to the continuous reconstructed field.

Supplementary Figure S6. Porosity benchmark for threshold and CNN reconstructions.

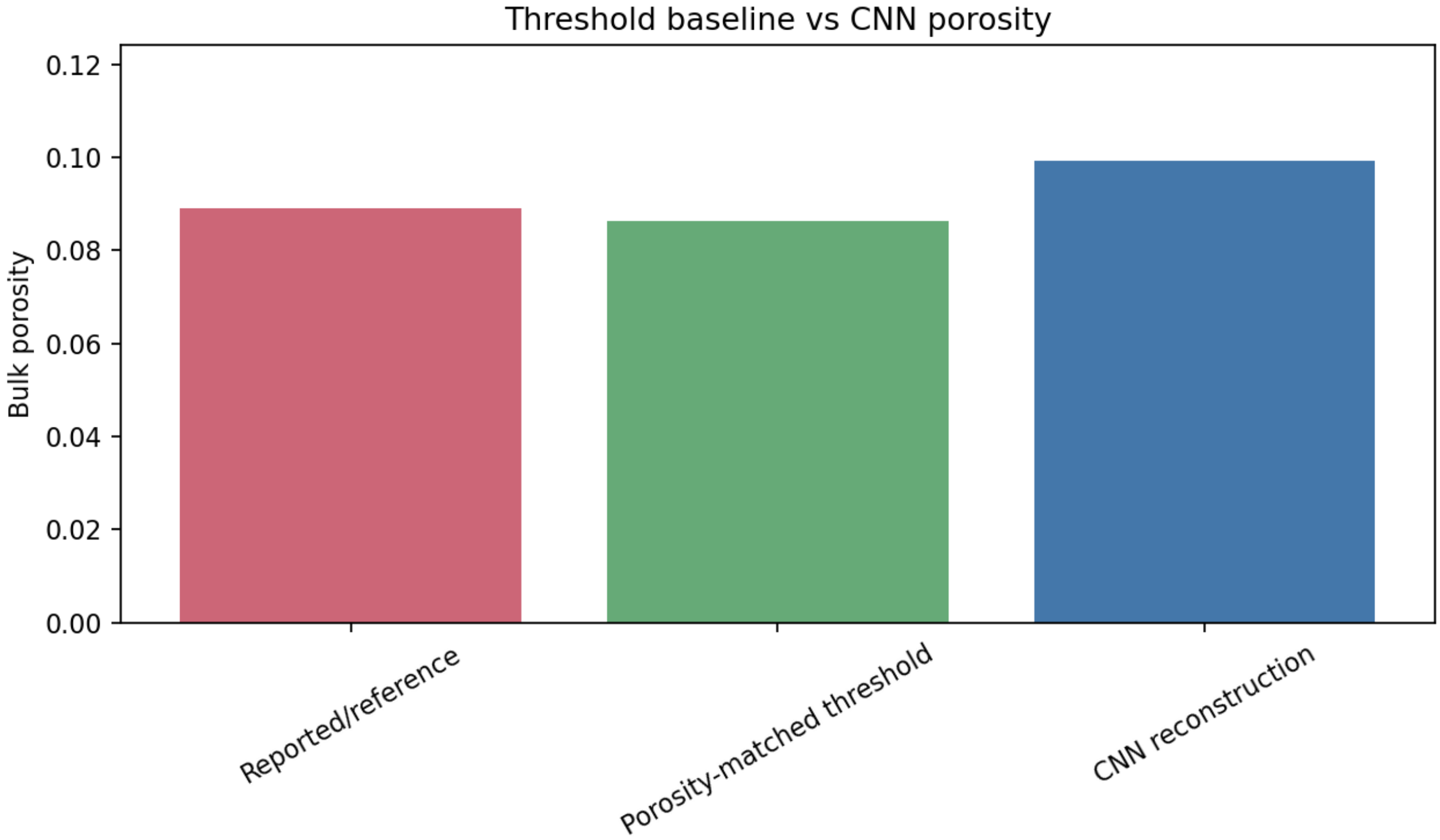

Bars compare bulk and connected porosity metrics for the threshold baseline and CNN reconstruction. The values show how pore volume and connectivity differ between the two reconstruction choices used for pore-scale flow analysis.

Supplementary Figure S7. Representative threshold versus CNN slice comparison.

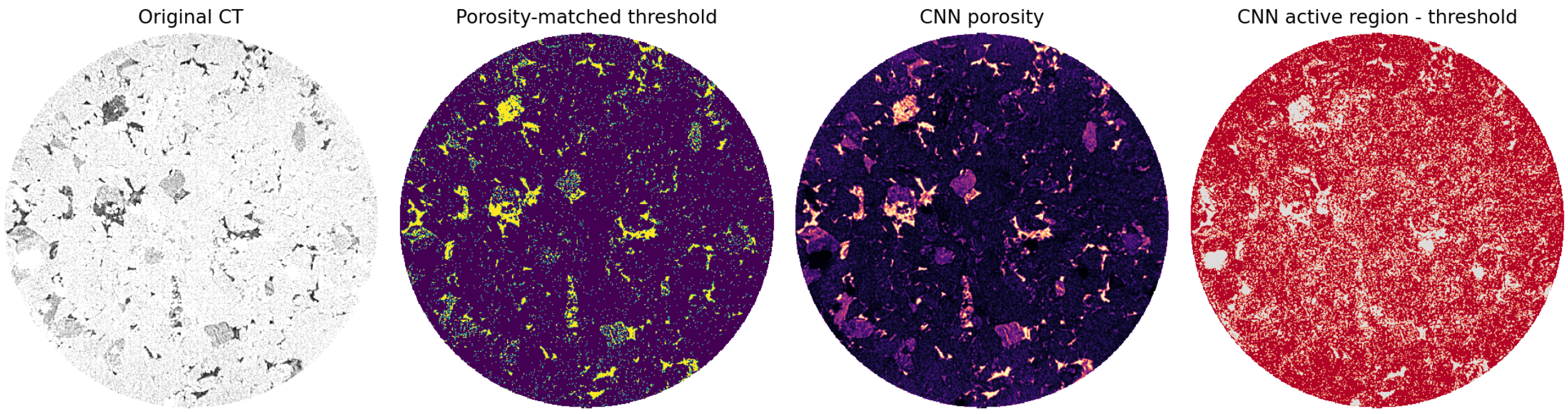

A representative slice contrasts porosity-matched threshold segmentation with the CNN reconstructed porosity field, illustrating why the threshold case is used as a conventional baseline rather than a ground-truth pore network.

Supplementary Note S3 Parameter sensitivity analysis

The following figures test how hydraulic-radius truncation, Knudsen/slip-flow ranges, water-distribution parameters, and thermodynamic conventions affect the numerical results. Together, they separate parameter-sensitive quantities from trends that remain stable across the tested ranges.

Permeability and extraction quantities reported here are diagnostic or proxy quantities unless explicitly stated otherwise.

Supplementary Figure S8. Water-distribution parameter sensitivity of the recovery proxy.

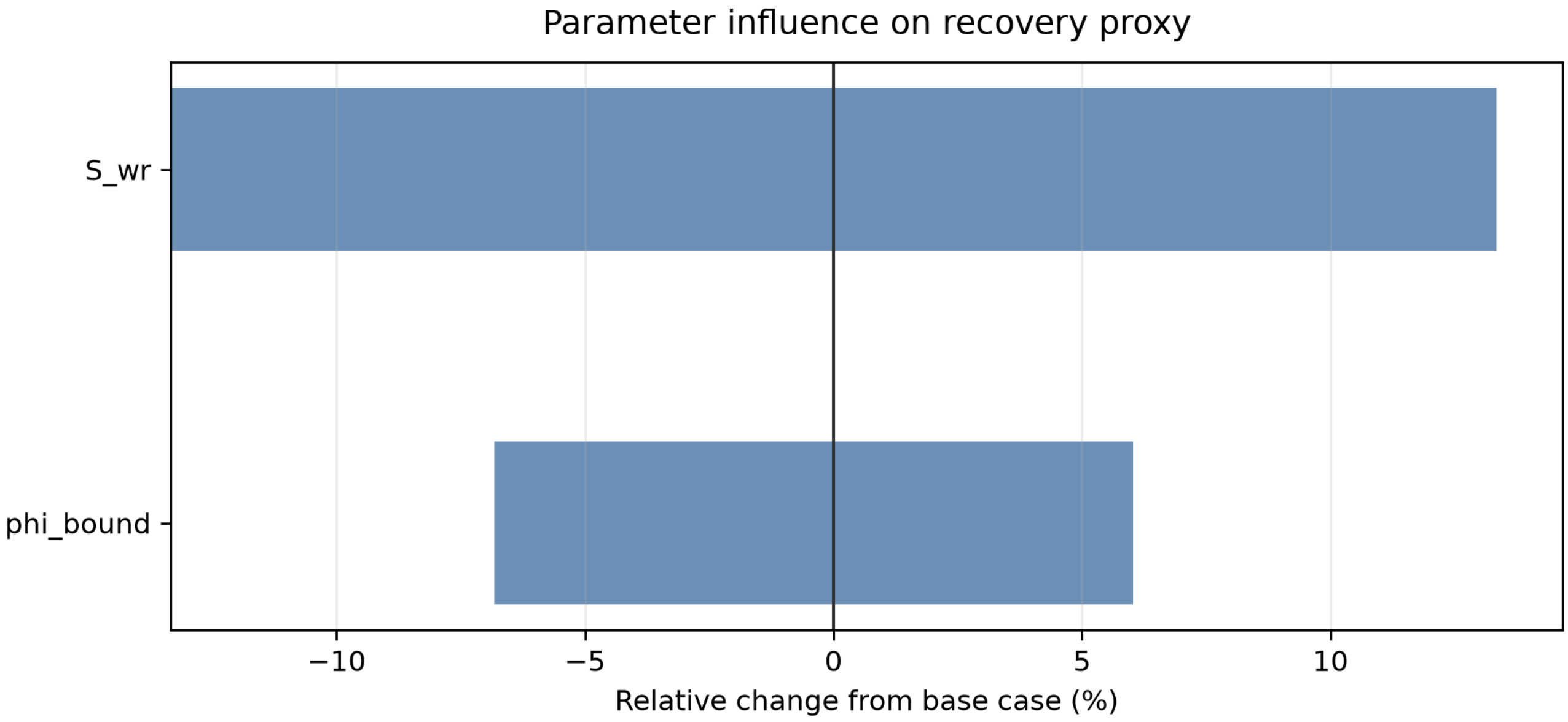

Horizontal bars show the relative change in the recovery proxy when phi_bound and S_wr are varied from the base case. Negative and positive bar ends mark the lower and upper tested parameter settings, showing that water distribution mainly changes available dissolved-helium inventory and the extraction response.

Supplementary Figure S9. Default-range hydraulic-radius cutoff sensitivity of Knudsen-number distributions.

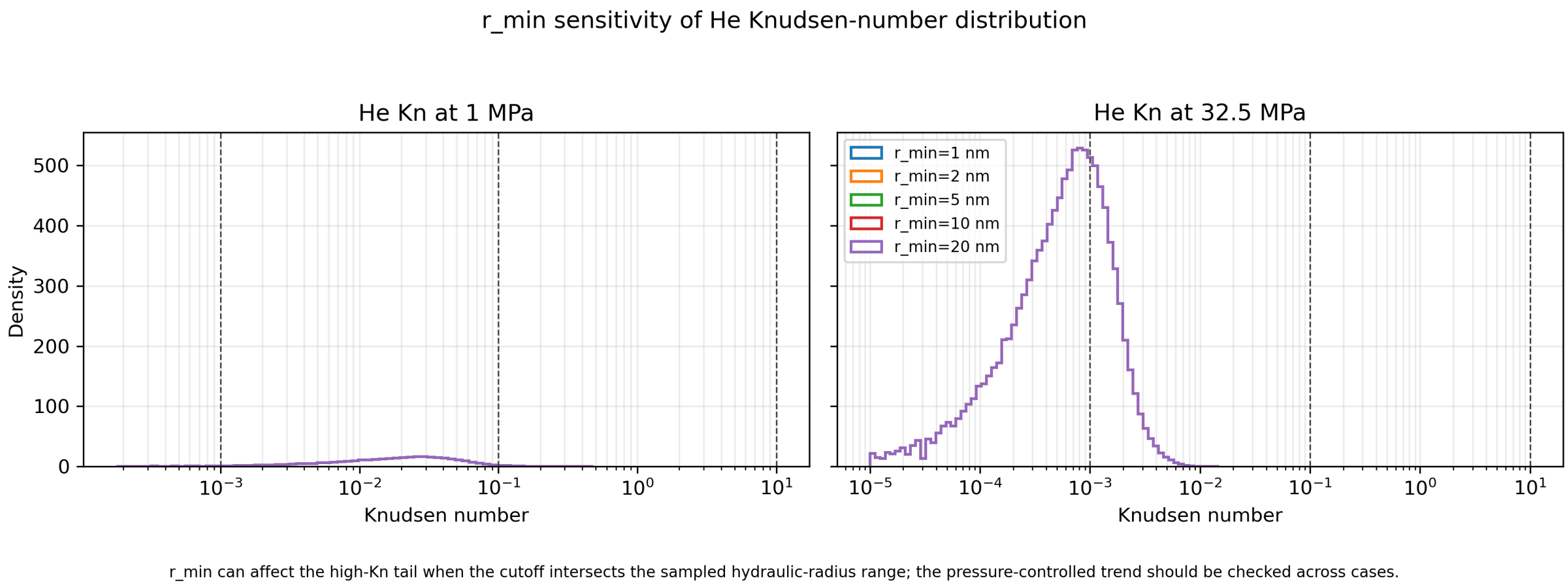

He Knudsen-number distributions for r_min values in the physically plausible 1-20 nm range overlap closely, indicating that the lower cutoff is below the main sampled hydraulic-radius population for this pore field.

Supplementary Figure S10. Default-range r_min sensitivity of median Kn and flow-regime fractions.

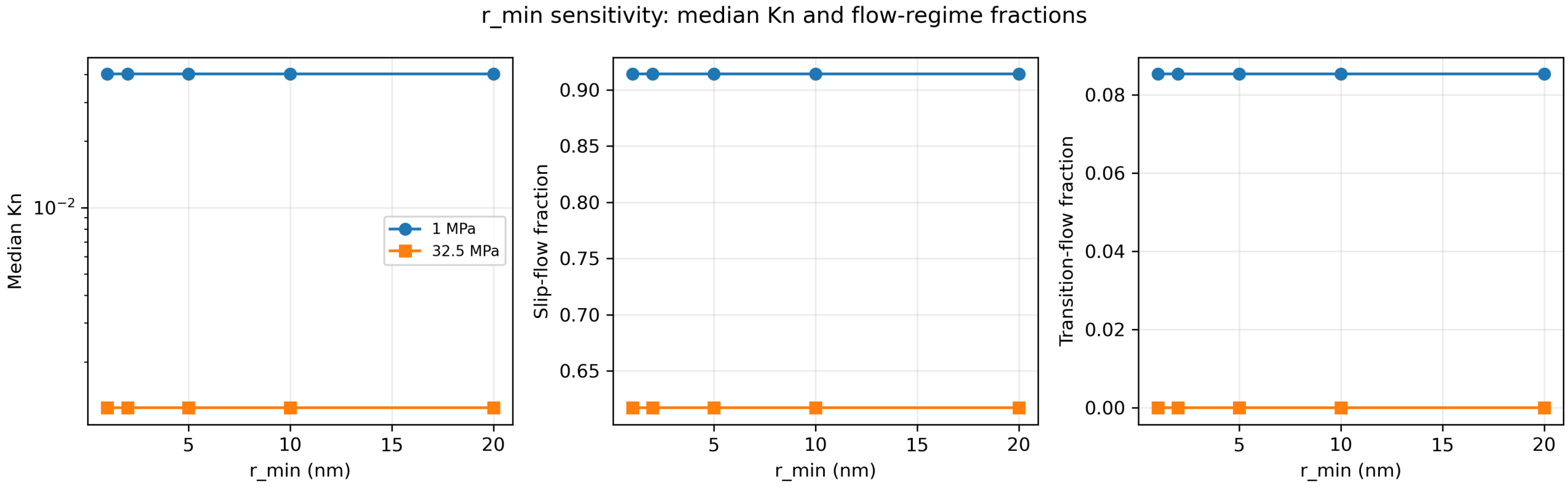

Lines show median Knudsen number and continuum/slip/transition fractions as r_min is varied from 1 to 20 nm. The flat response indicates that the main flow-regime assignment is not controlled by the cutoff in the default range.

Supplementary Figure S11. Extended r_min stress test of Knudsen-number distributions.

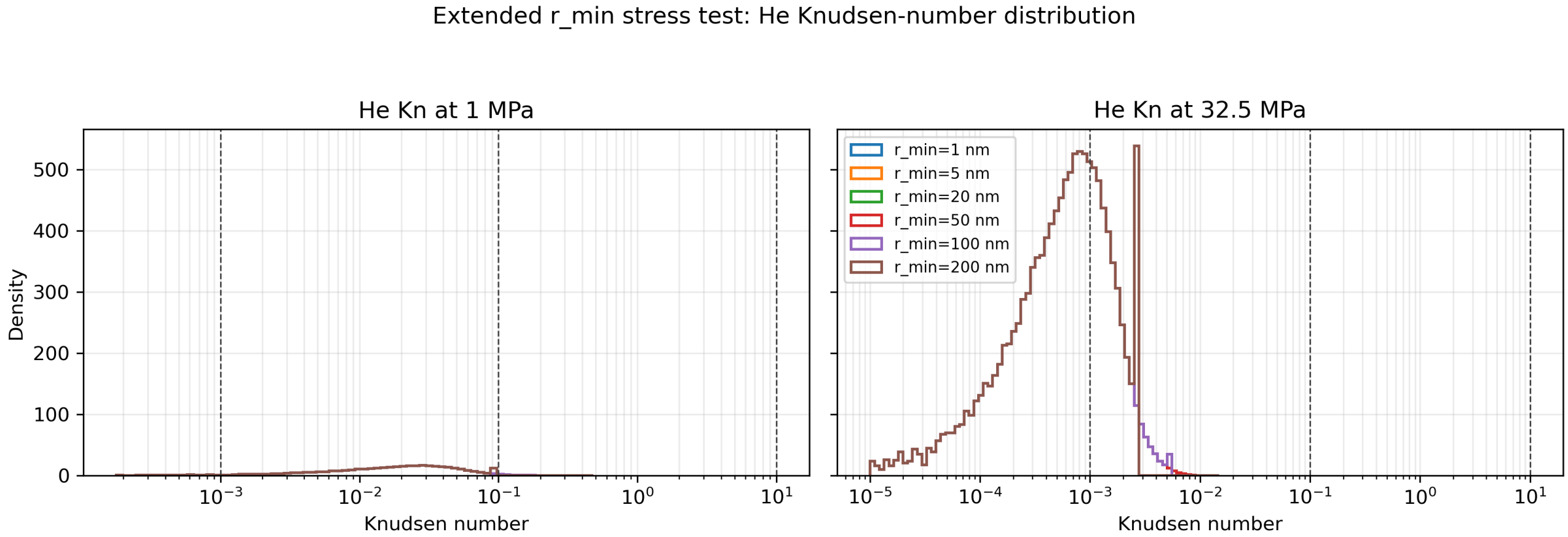

The extended r_min stress test is used to examine when hydraulic-radius truncation begins to affect the Kn distribution. It is not treated as the default calibrated parameter range.

Distribution curves show the He Knudsen-number response when r_min is increased beyond the calibrated range. Shifts appear only when the cutoff approaches the sampled hydraulic-radius population, so this panel is used as a stress test rather than a calibrated parameter range.

Supplementary Figure S12. Extended r_min stress test of median Kn and flow-regime fractions.

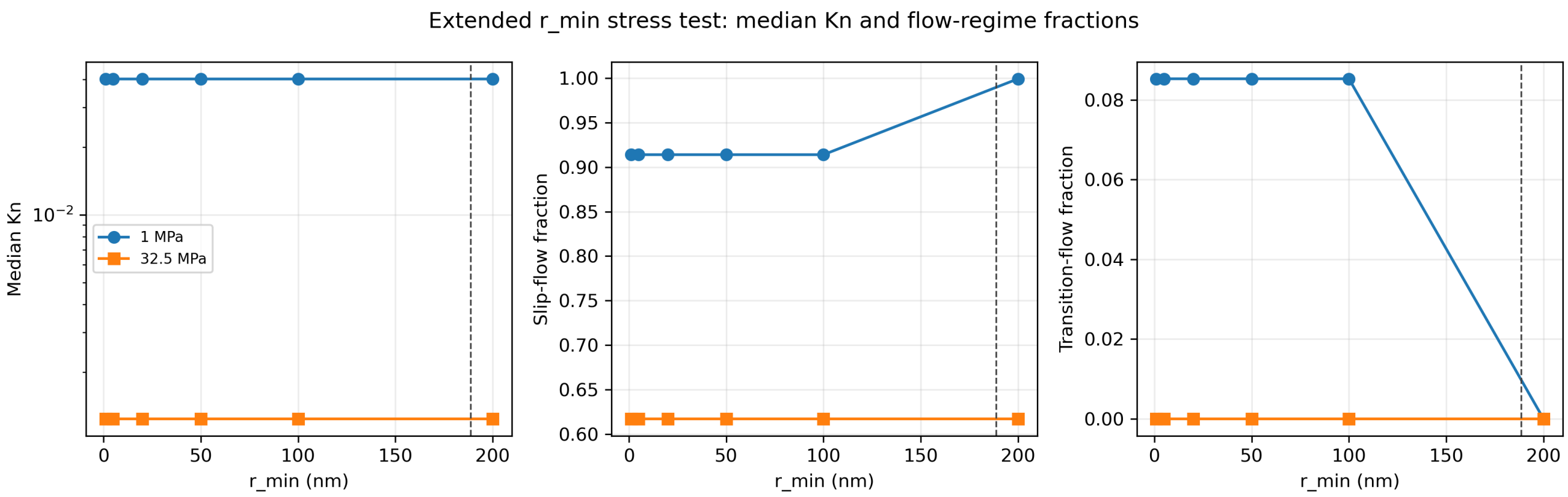

Median Knudsen number and flow-regime fractions are summarized for the extended truncation test, showing that unrealistically large cutoffs can affect regime fractions whereas the default range remains stable.

Supplementary Figure S13. Knudsen-number distribution with slip-correction applicability range.

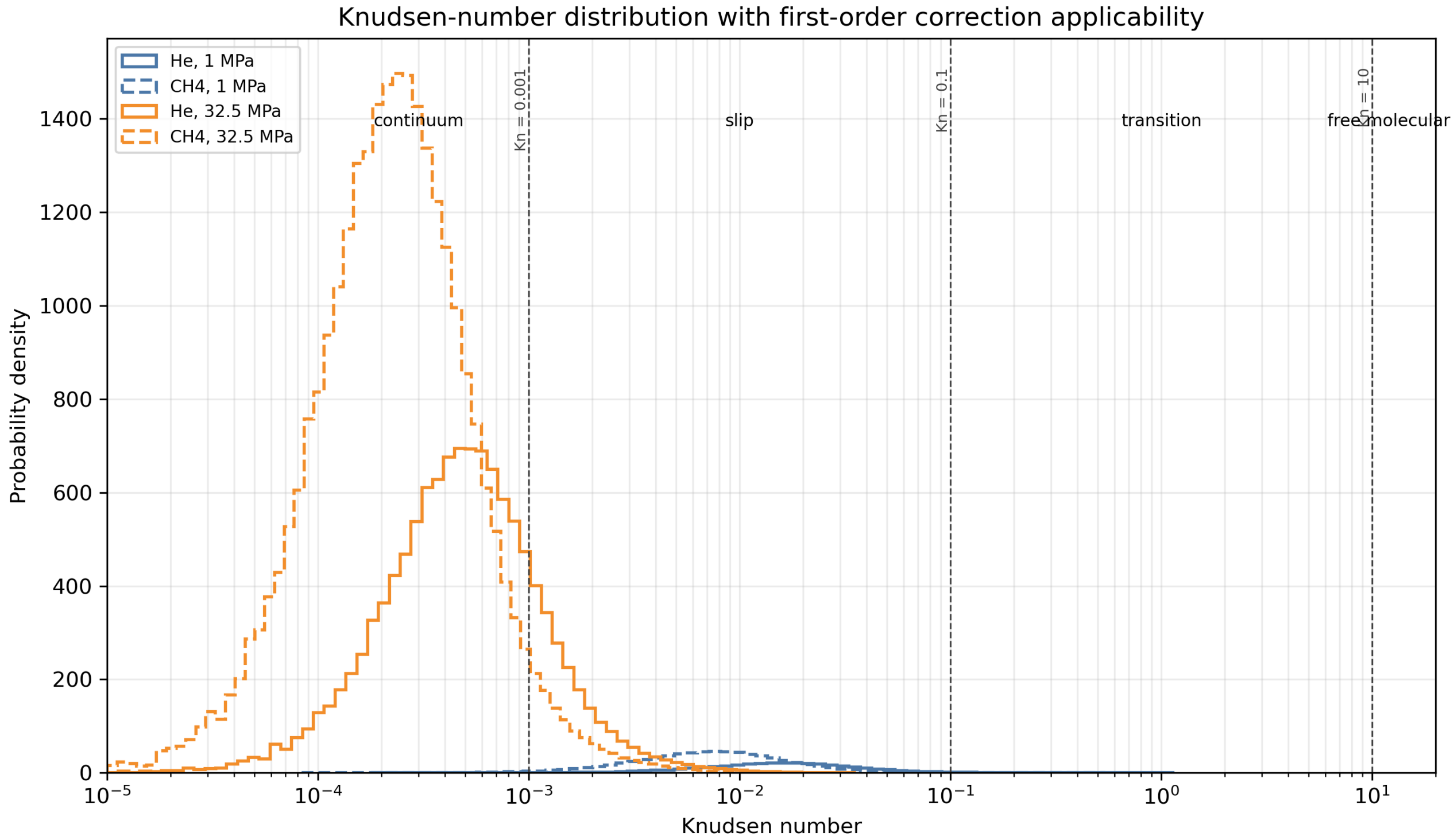

Knudsen-number distributions are annotated by continuum, slip-flow, and transition-flow regimes to define where the first-order apparent-permeability correction can be interpreted quantitatively.

Supplementary Figure S14. Pressure dependence of Knudsen-regime fractions.

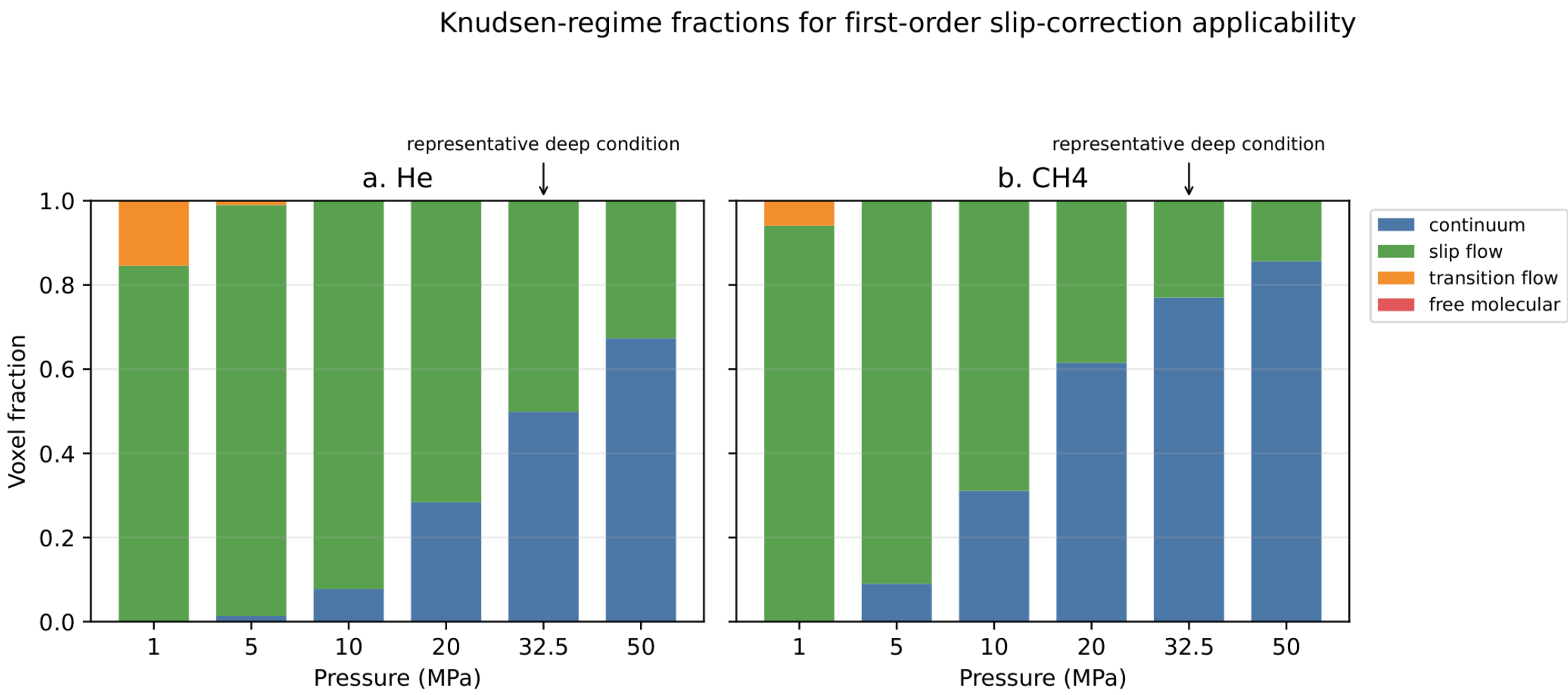

Colored bars show the fractions of active pore voxels in continuum, slip-flow, and transition-flow regimes from 1 to 50 MPa. The pressure trend defines where the first-order slip correction is treated as quantitative or semi-quantitative.

Supplementary Figure S15. Spatial maps of Knudsen regimes at low and deep-reservoir pressure.

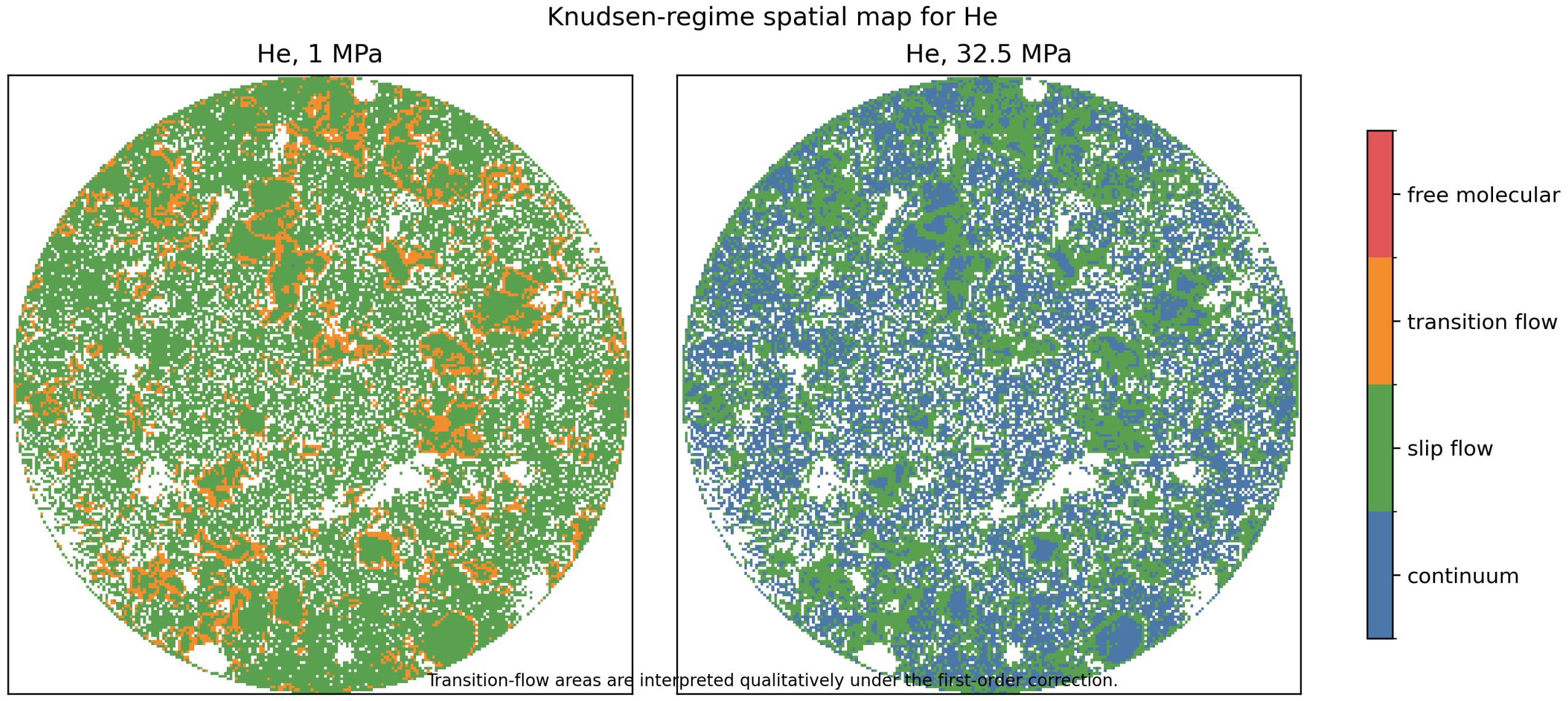

Spatial distributions of Knudsen regimes at 1 MPa and 32.5 MPa show that transition-flow contributions are larger at low pressure, whereas representative deep pressure is dominated by continuum/slip-flow conditions.

Supplementary Figure S16. Summary of first-order slip-correction applicability.

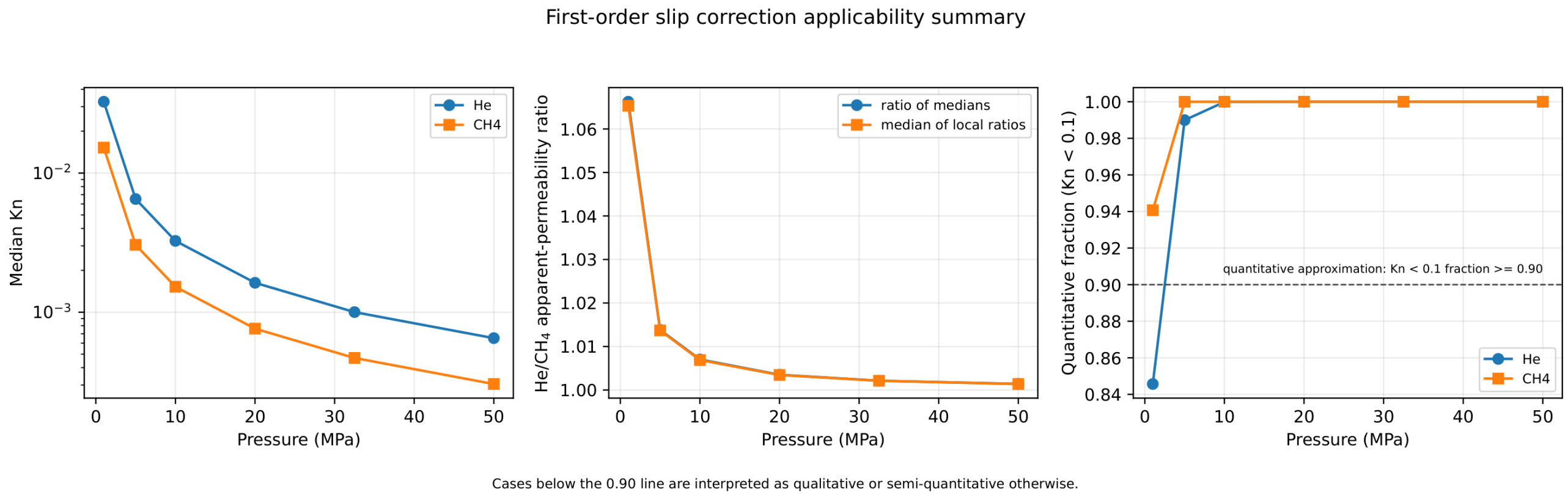

Pressure-dependent applicability metrics summarize the fraction of active pore space where first-order apparent-permeability correction is suitable for quantitative interpretation.

Supplementary Figure S17. Helium salting sensitivity at representative reservoir conditions.

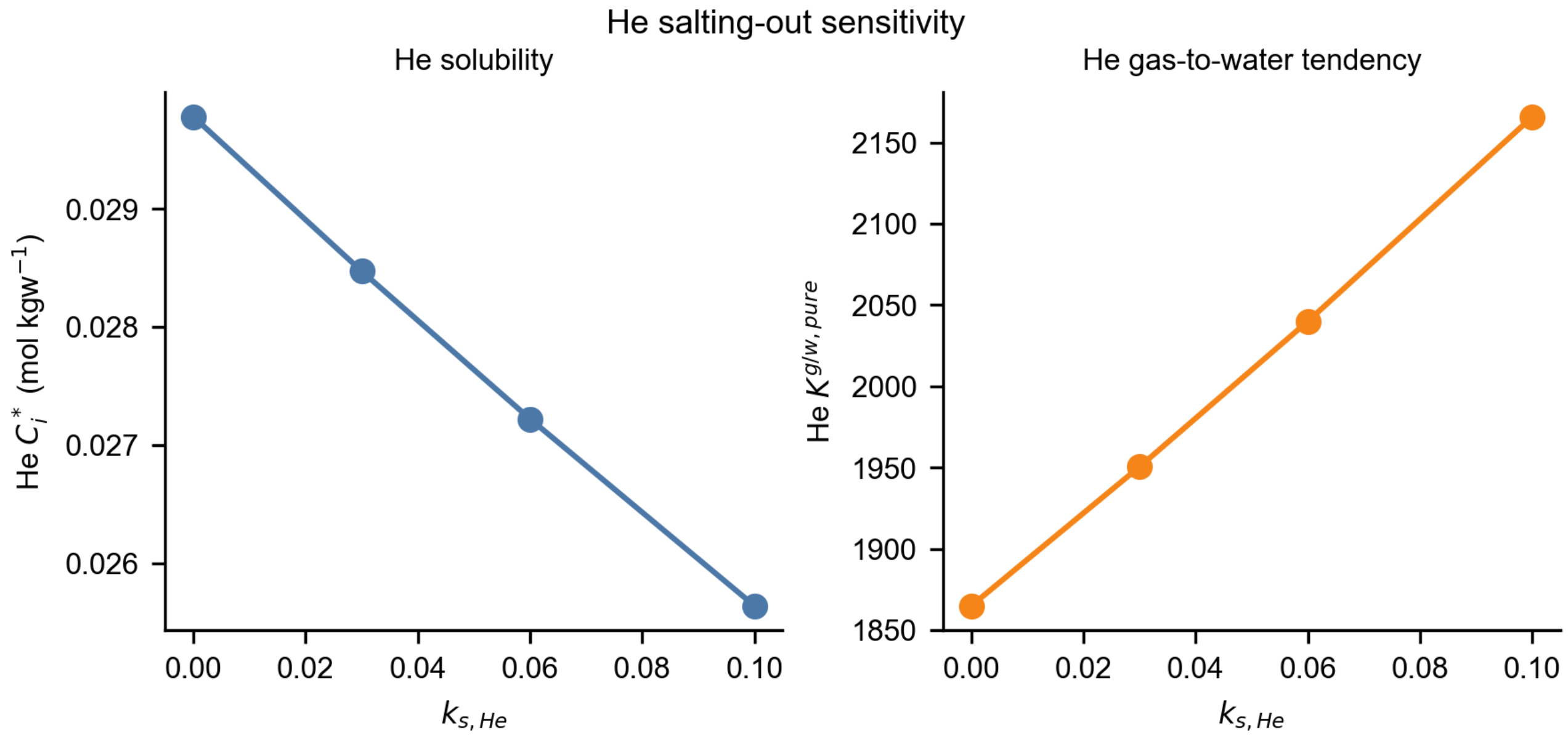

Sensitivity of calculated helium solubility and gas-water partitioning to the Setschenow salting correction at 113 °C, 32.5 MPa, and representative brine ionic strength.

Supplementary Figure S18. Gas-water partition coefficient direction check.

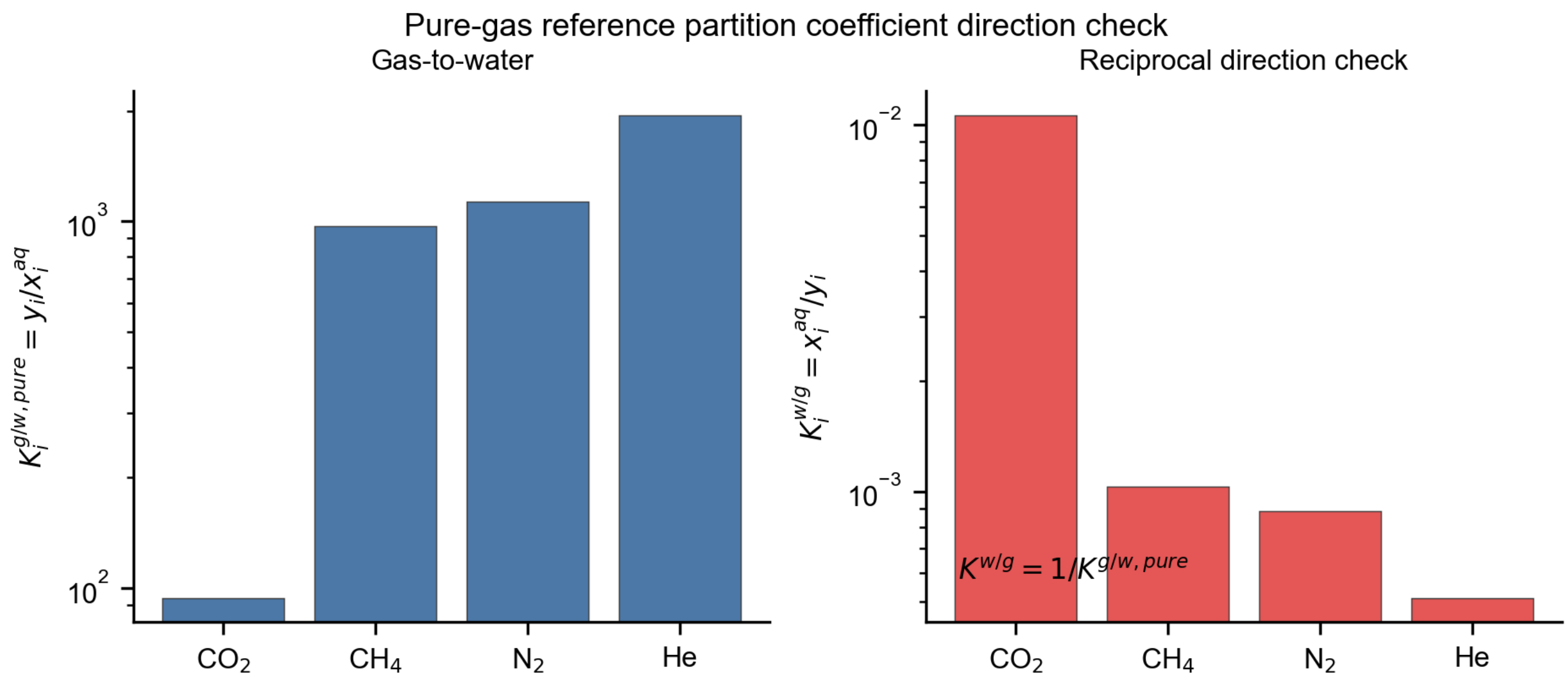

Revised convention check confirms the direction of gas-water partition coefficients and prevents inversion of solubility and stripping-response interpretations.

Supplementary Figure S19. Inverse relation between solubility and partition coefficient.

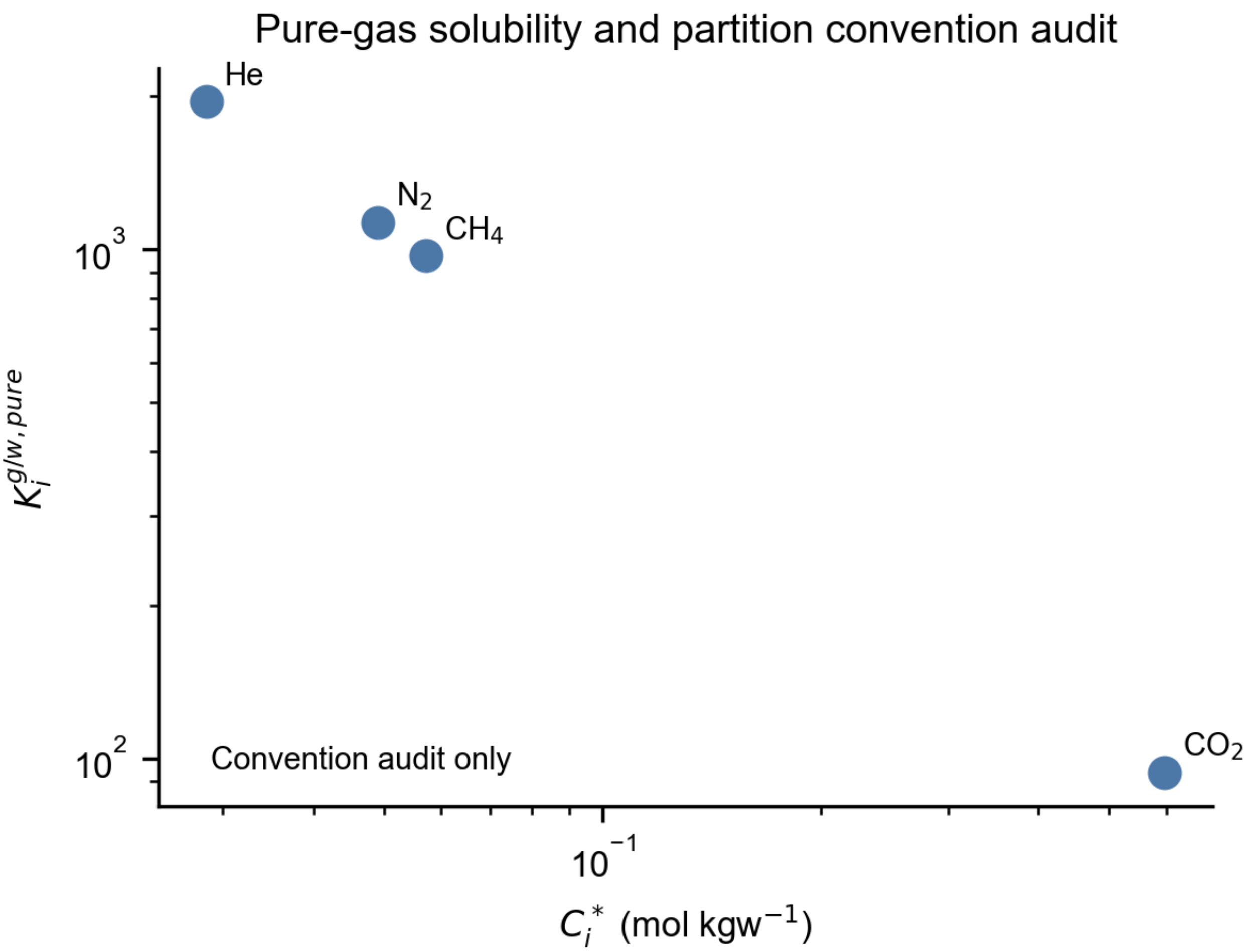

The plot relates aqueous solubility to the adopted gas-water partition coefficient. Higher solubility corresponds to lower effective gas-water partition tendency under this convention.

Supplementary Figure S20. Revised stripping-response sequence.

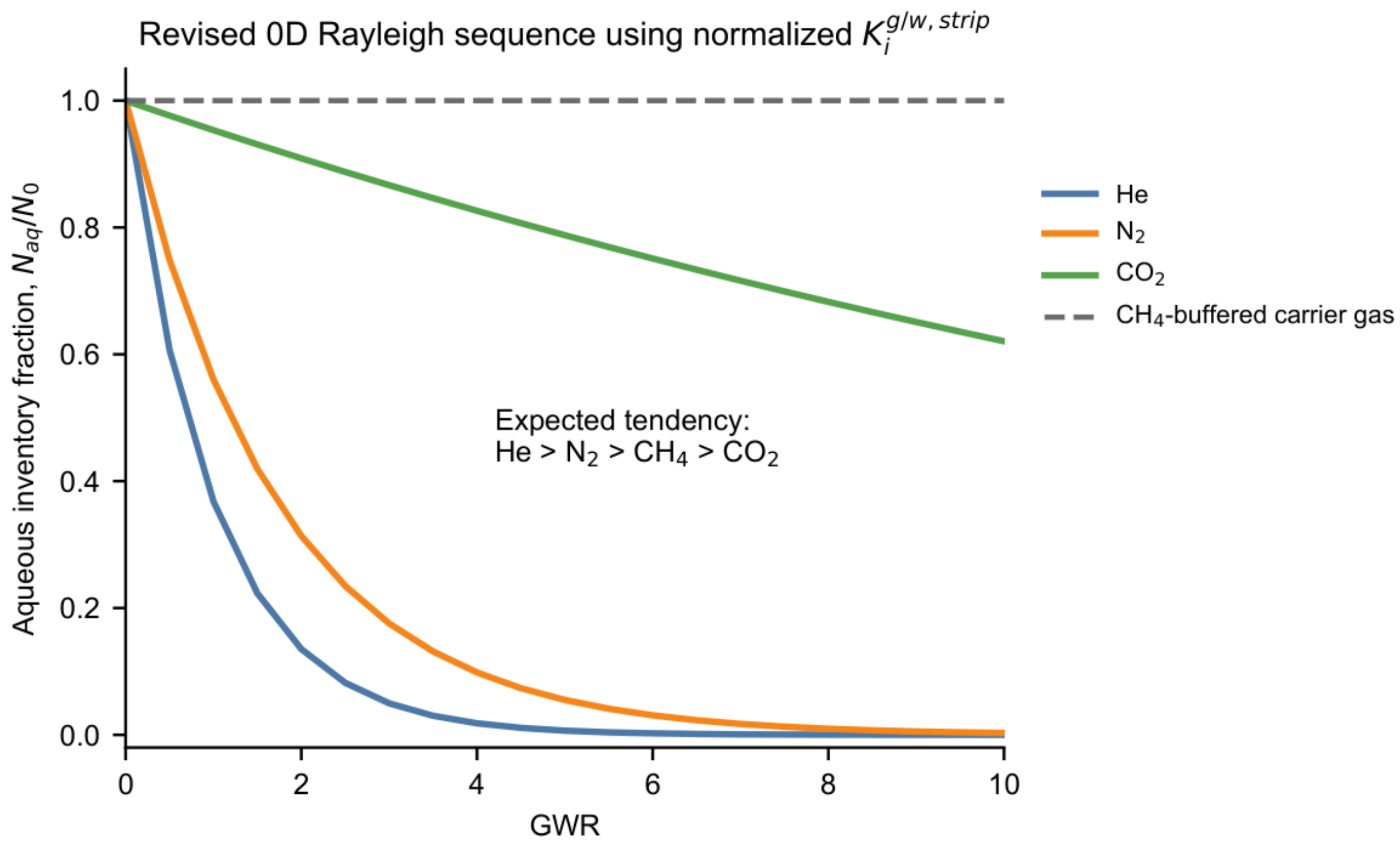

Zero-dimensional stripping-response curves compare passive gas behavior and aqueous-inventory depletion under the revised gas-water partition convention.

Supplementary Figure S21. Thermodynamic partitioning convention audit.

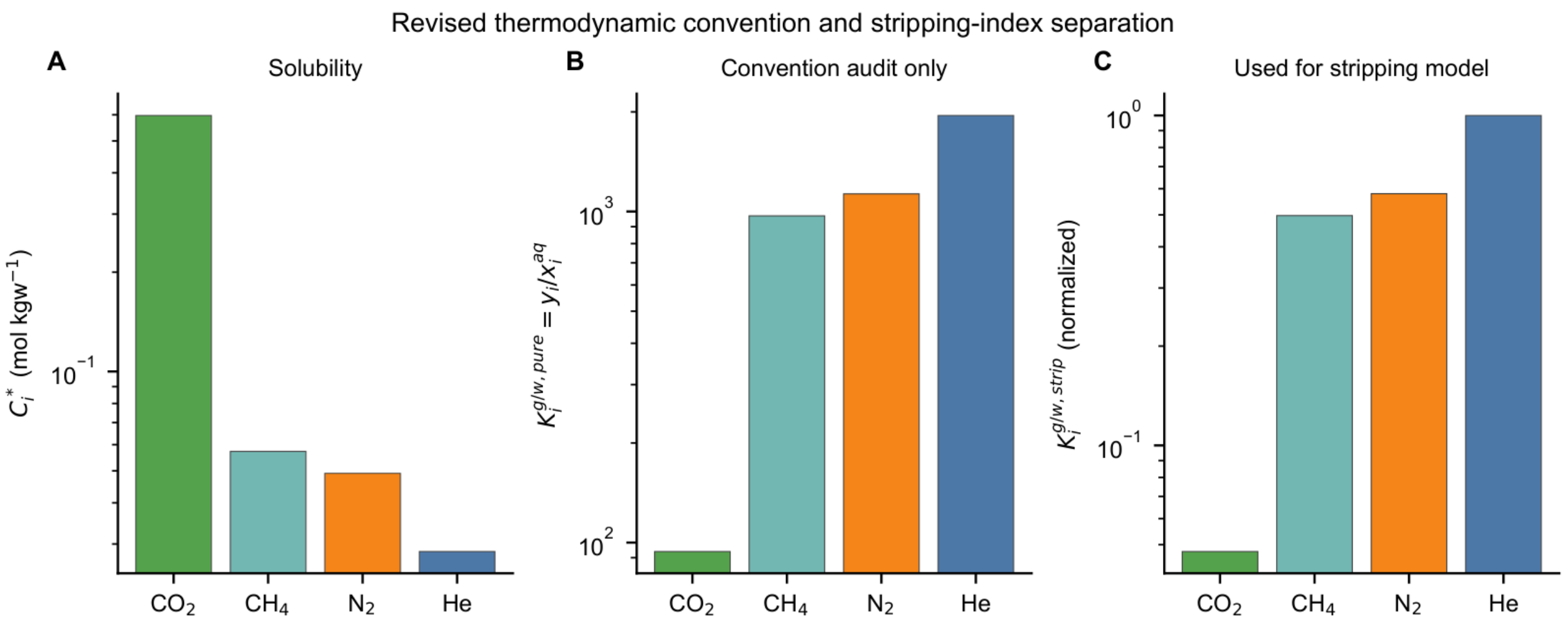

Representative-condition gas-water partition factors are audited to ensure consistent use of Henry, fugacity, Poynting, and salting corrections in the revised manuscript.

Supplementary Figure S22. Thermodynamic correction factors at representative conditions.

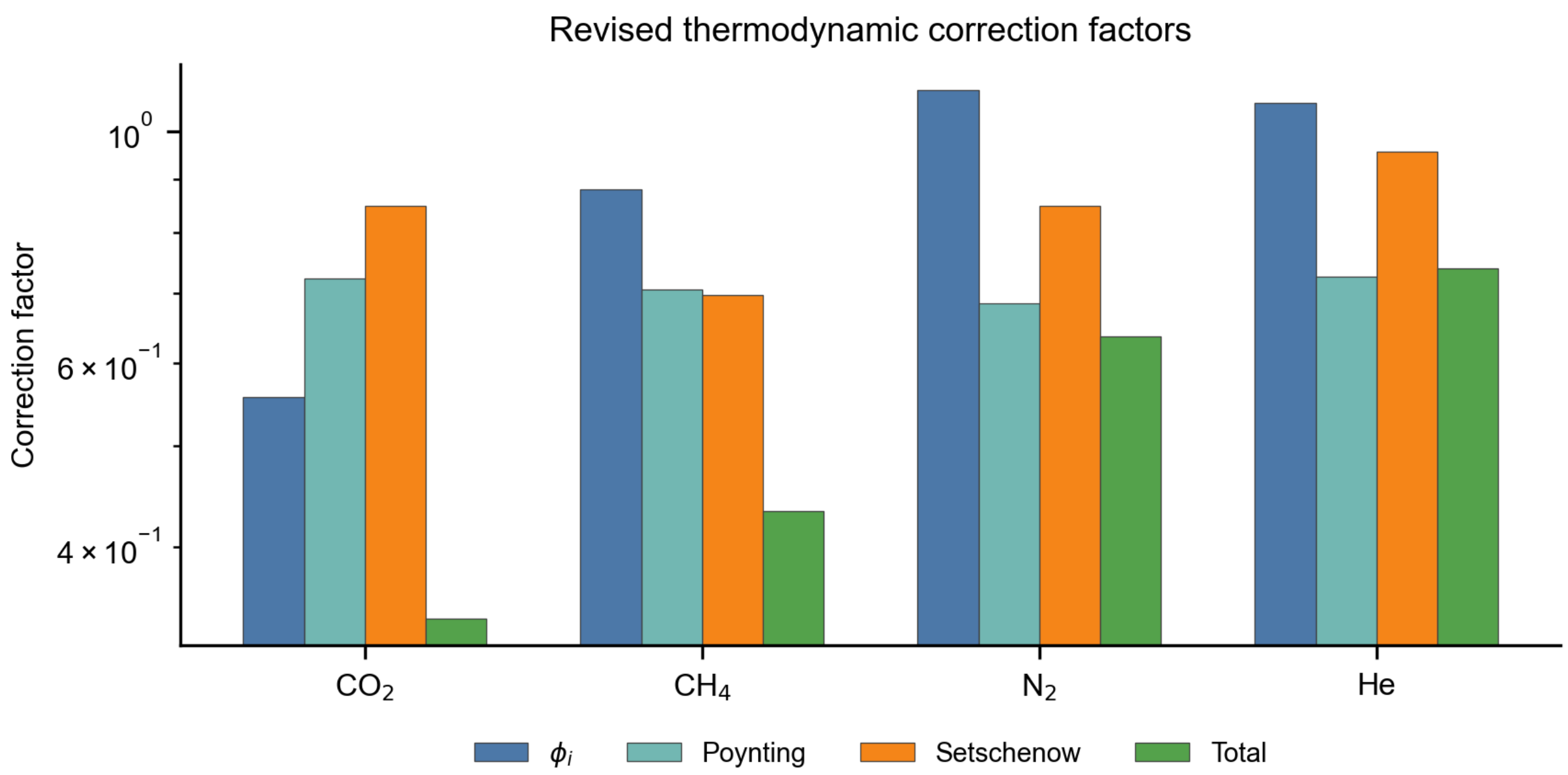

Grouped bars summarize fugacity, Poynting, and salting correction factors for each gas at the representative condition. The panel shows which correction terms control the revised partition calculation.

Supplementary Figure S23. Local-scale slippage sensitivity heatmap.

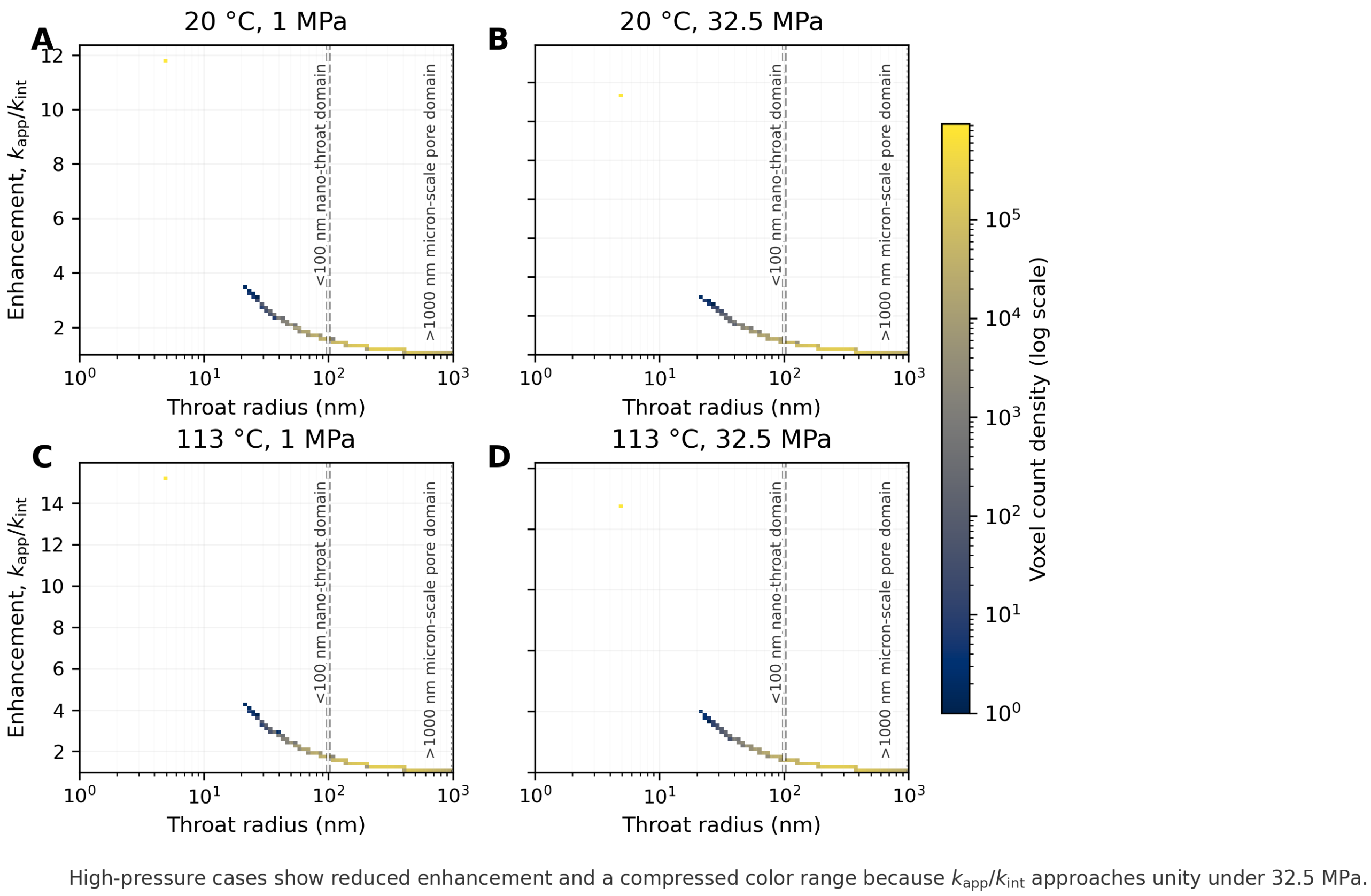

Two-dimensional density heatmaps show pore-throat radius versus apparent-permeability enhancement factor for He under pressure-temperature conditions using local color scaling to improve within-panel readability.

Supplementary Figure S24. Unified-scale slippage sensitivity heatmap.

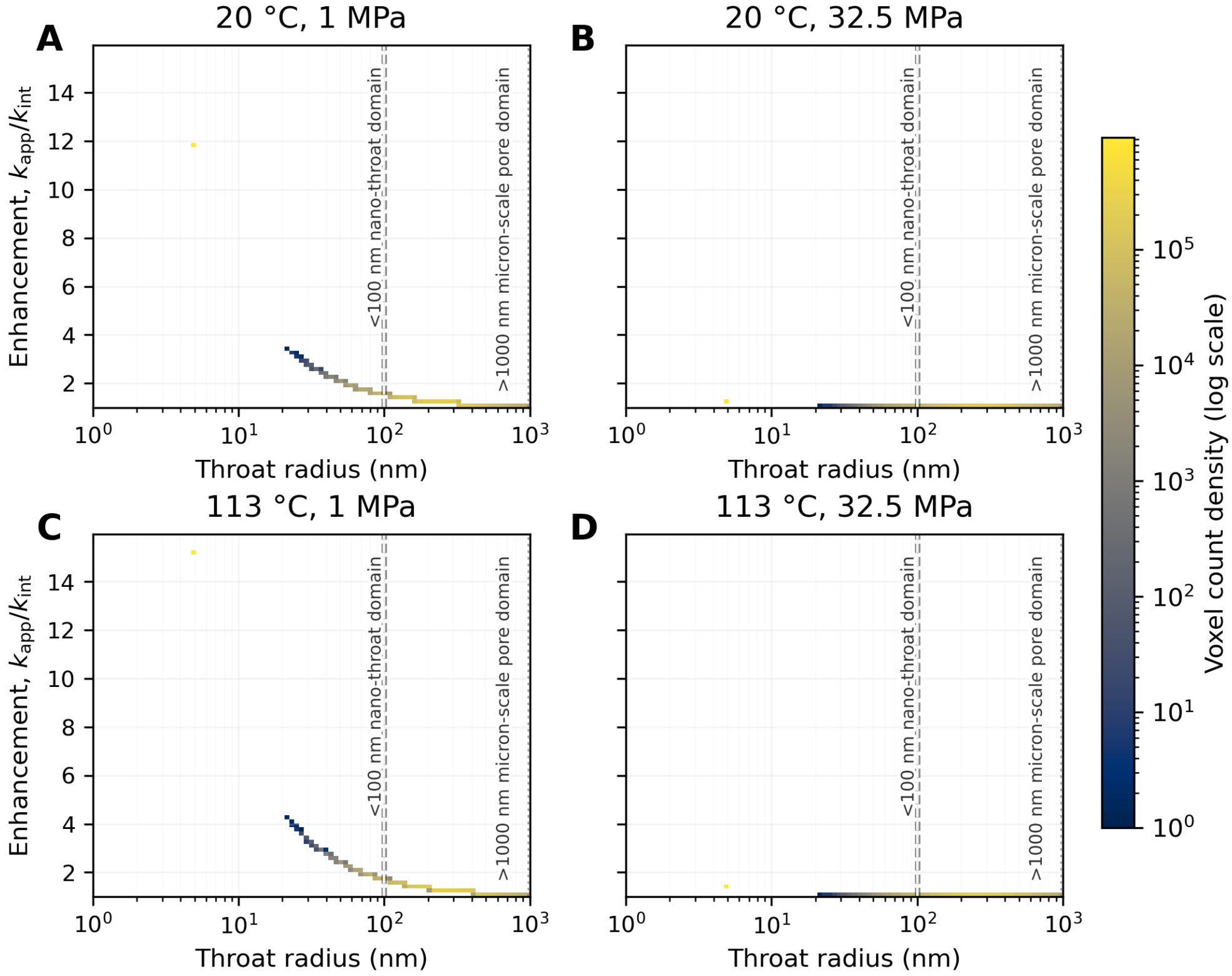

High-pressure cases show reduced enhancement and a compressed color range because k_{app}/k_{int} approaches unity under 32.5 MPa.

Two-dimensional density heatmaps show pore-throat radius versus apparent-permeability enhancement factor for He under pressure-temperature conditions using a shared logarithmic color scale for cross-panel comparison.

Supplementary Note S4 Multi-slice extraction validation

The following figures compare nine rotated 2D slices used to check whether the extraction trend depends strongly on the selected slice. The same temperature, pressure, salinity, injection setting, and local transfer parameters were used for all slices.

The numerical data underlying Supplementary Figures related to multi-slice extraction are provided in Supplementary Data 1: Supplementary_Data_multislice.xlsx.

Supplementary Figure S25. Multi-slice methane injection-state panel.

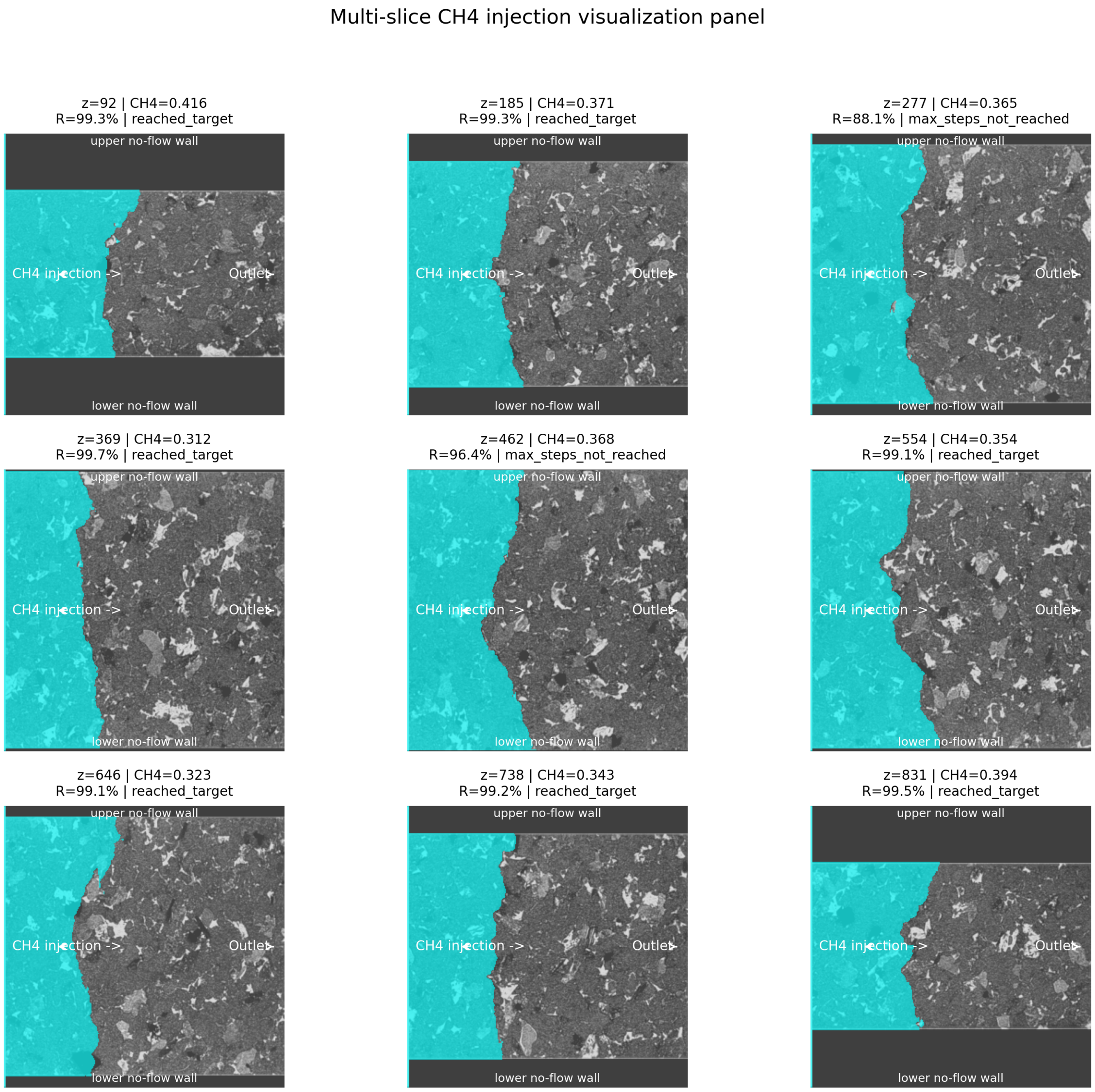

Cyan regions mark methane-rich cells for the nine rotated extraction slices. The panels show how methane invasion patterns vary with slice position under the same left-to-right injection setting.

Supplementary Figure S26. Multi-slice final helium state panel.

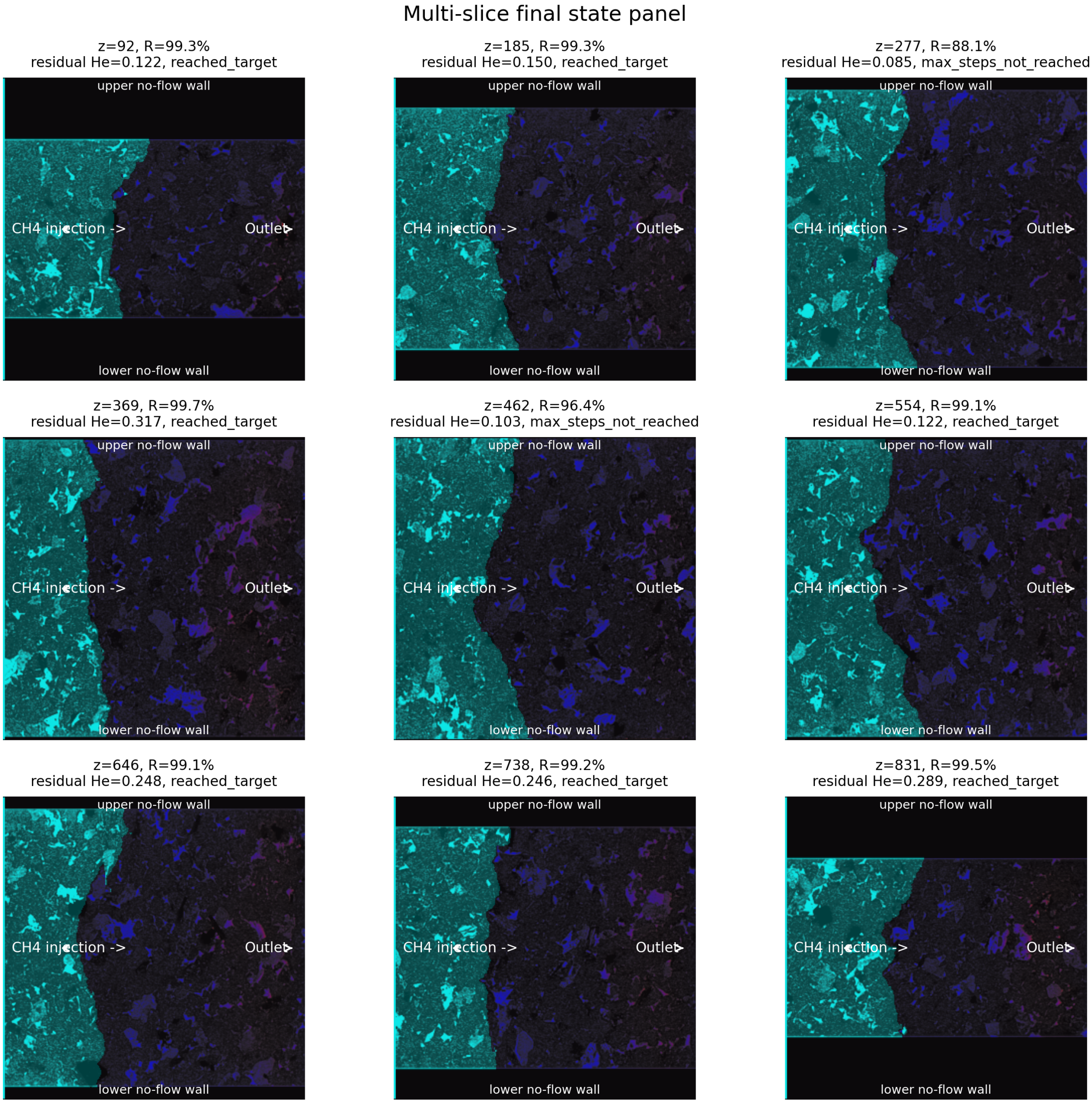

Final residual-helium fields are compared across the nine extraction slices to assess whether end-state helium retention is controlled by a single slice or remains consistent across the multi-slice ensemble.

Supplementary Figure S27. Multi-slice porosity panel.

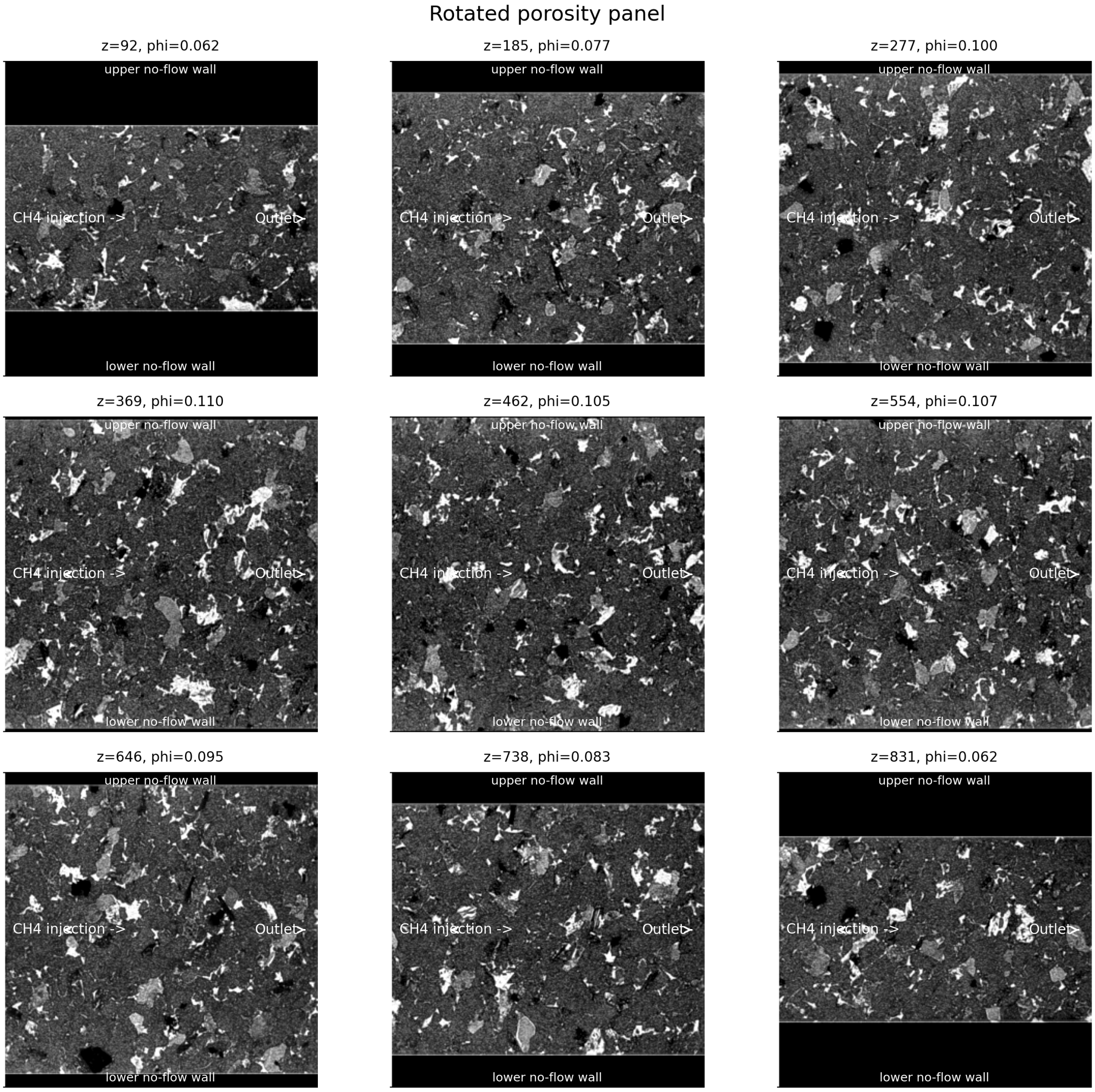

Grey-scale panels show reconstructed porosity fields for the nine rotated slices. The slice set captures structural differences in pore space before the extraction calculation is repeated.

Supplementary Figure S28. Multi-slice helium recovery curves.

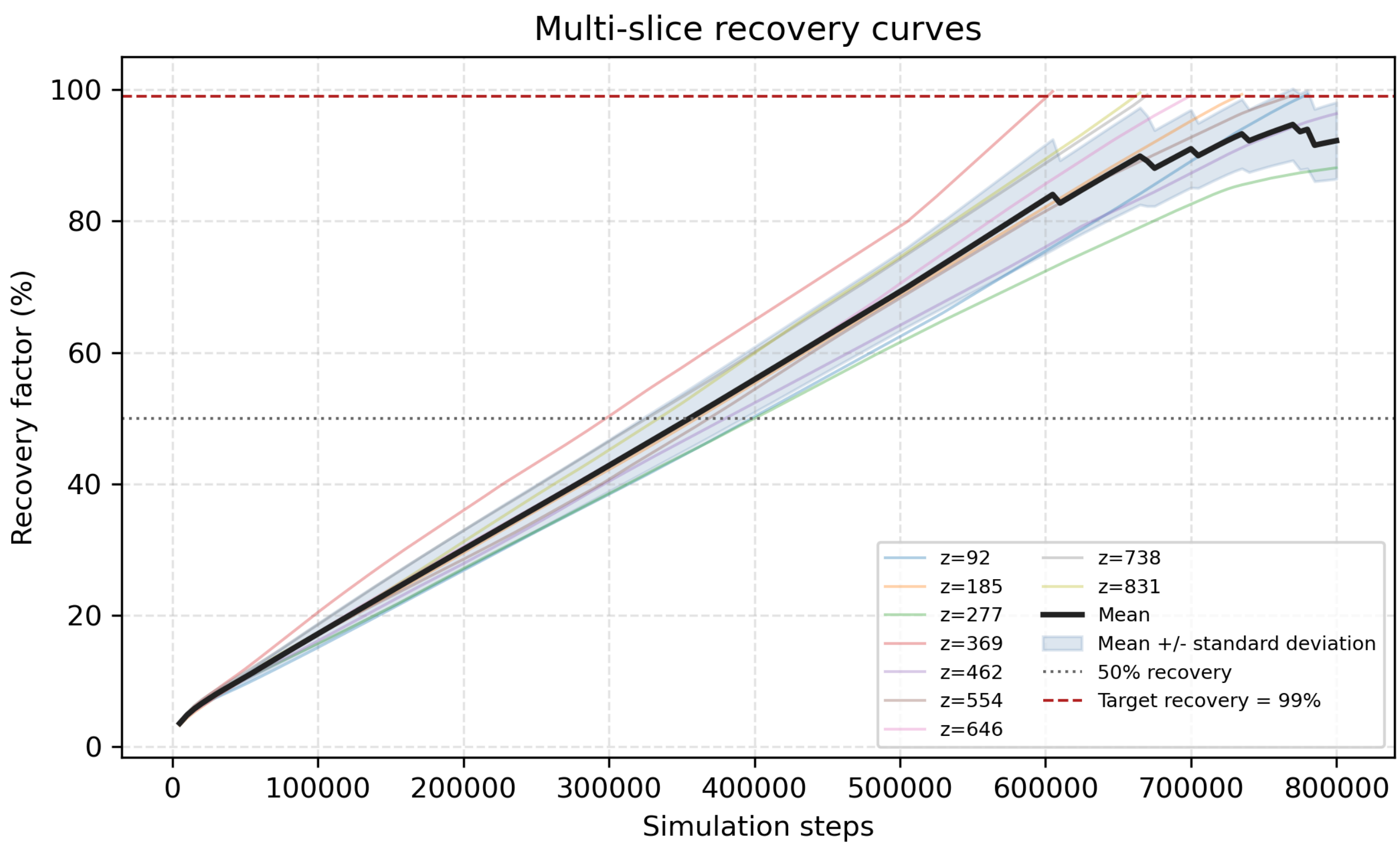

Simulations were run until the target recovery, plateau criterion, or maximum prescribed steps was reached.

Recovery-factor trajectories for the nine rotated slices quantify slice-to-slice variability in methane-driven helium extraction under identical simulation settings.

Supplementary Figure S29. Multi-slice summary metrics.

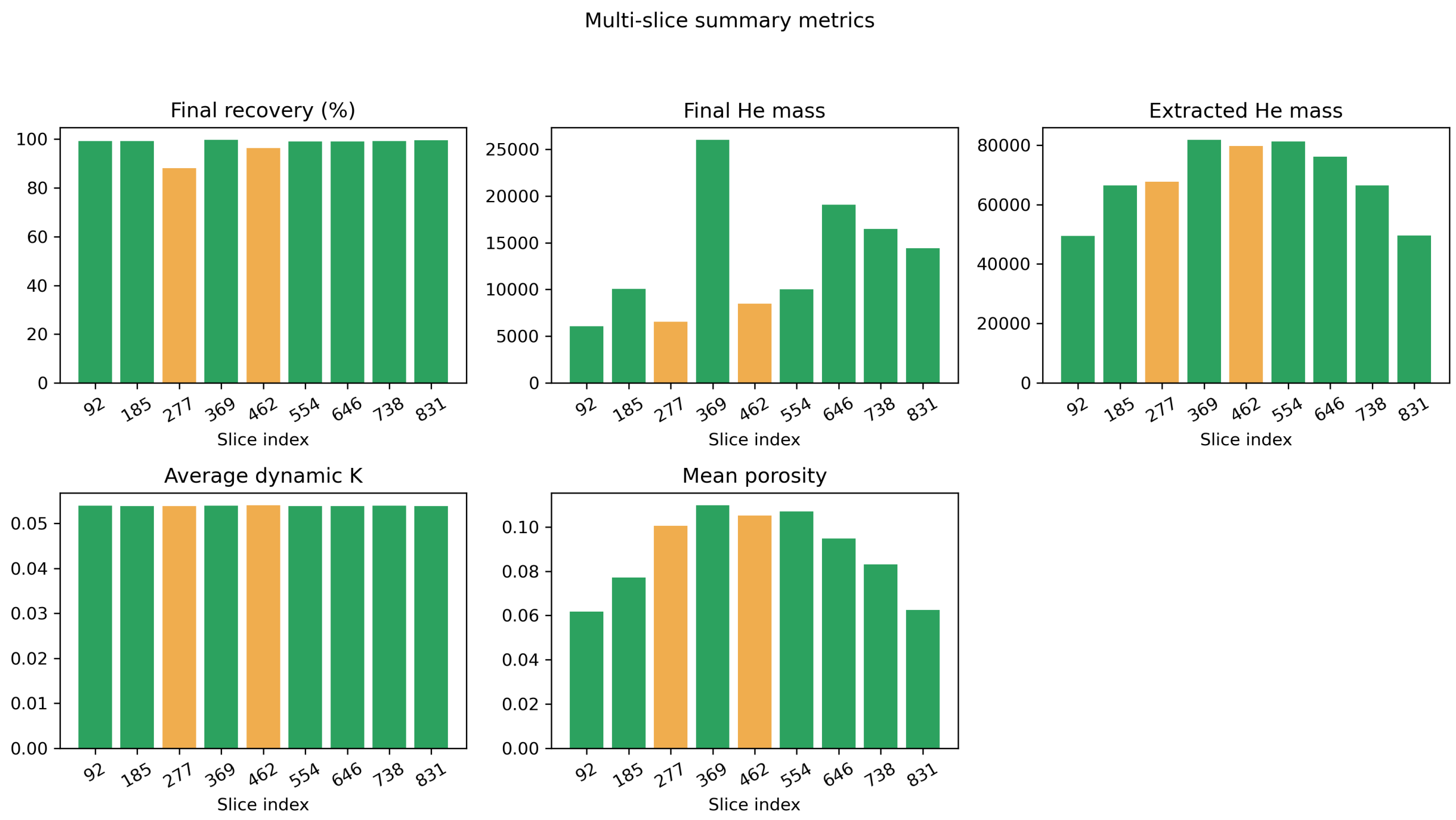

Final recovery, steps to 50 percent recovery, residual helium fraction, and methane contact-area fraction are summarized across the nine slices as mean and slice-level values.

Supplementary Note S5 Local transfer coefficient and Sherwood-Péclet diagnostics

The following figures show how the local transfer term was applied in gas-water contact cells and how its parameters affect the extraction response. The reported k_loc is a dimensionless per-step removal fraction in lattice units and is not a dimensional mass-transfer coefficient.

Supplementary Figure S30. Spatial maps of local transfer and Sherwood-Péclet terms.

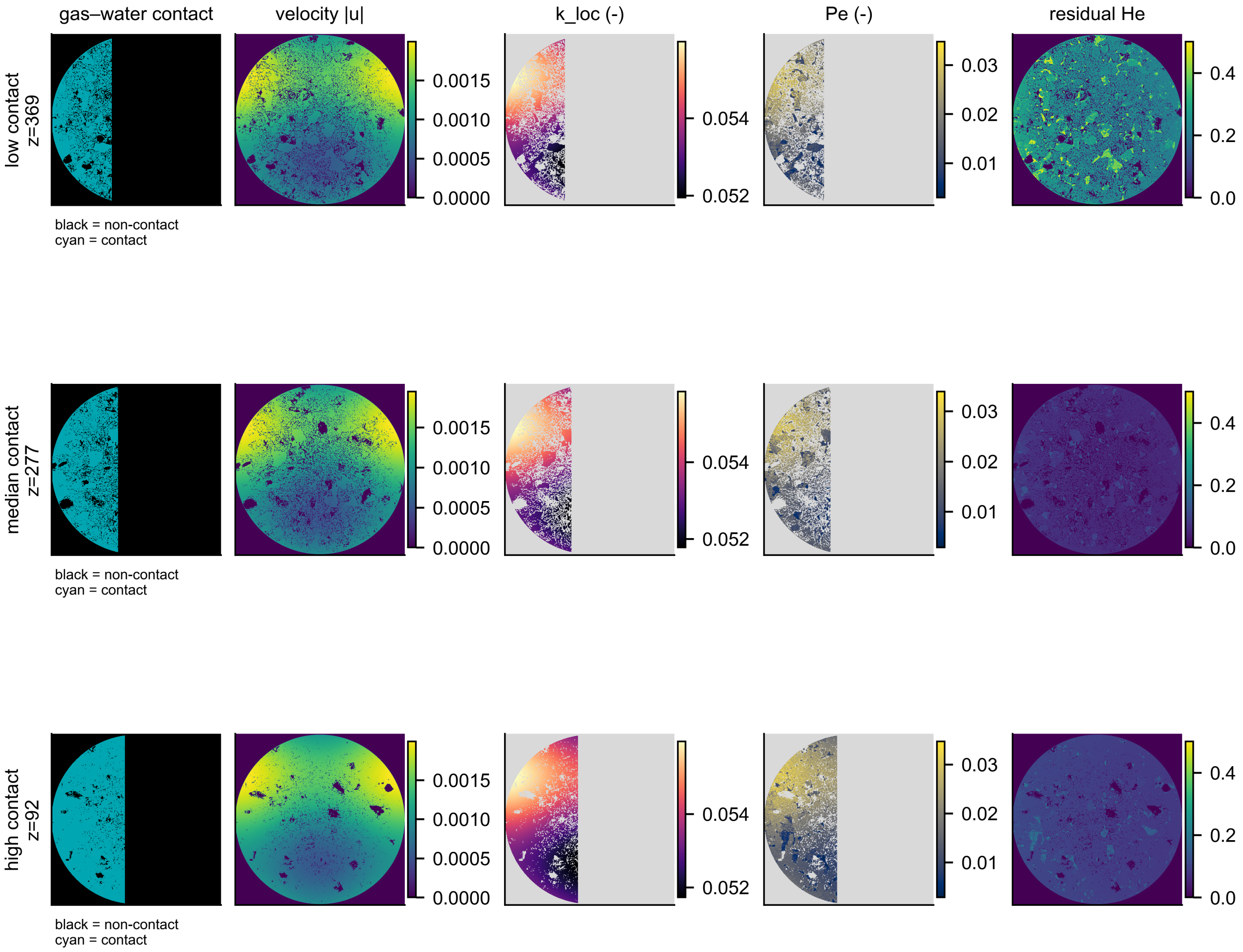

Columns show gas-water contact, velocity magnitude, k_loc, Péclet number, and residual helium for low-, median-, and high-contact slices. Grey or black masked regions are outside gas-water contact; k_loc and Péclet values are interpreted only in contact cells.

Supplementary Figure S31. Sensitivity of local transfer parameters k_diff and chi_u.

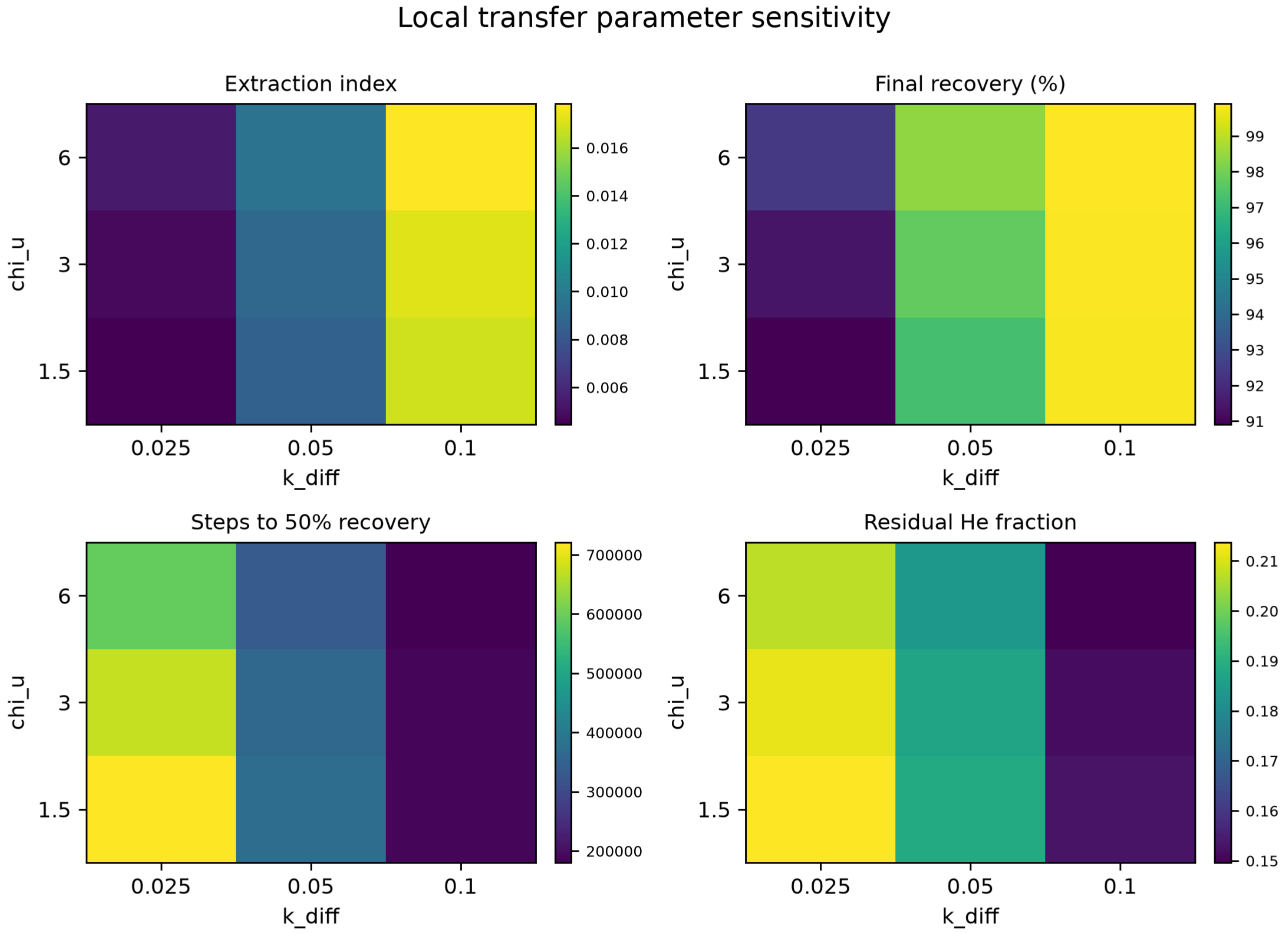

Heatmaps show how k_diff and chi_u change the extraction index, final recovery, steps to 50 percent recovery, and residual helium fraction across slices. The parameter grid uses the same methane-contact fields and therefore isolates the transfer-term response.

Supplementary Figure S32. LBM variable-transfer schematic.

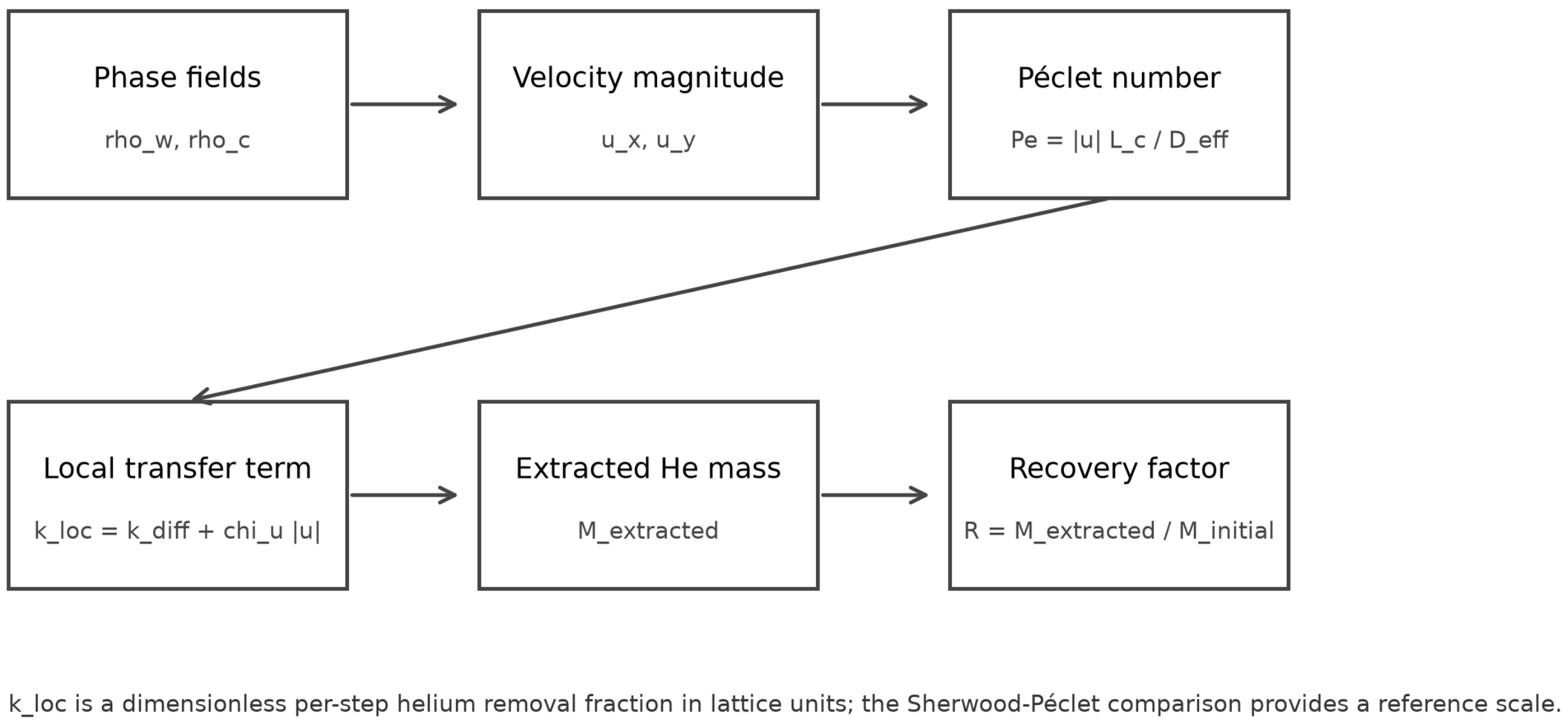

Boxes show the sequence from phase fields and velocity magnitude to Péclet number, $k_{loc} = k_{diff} + chi_u|u|$, extracted helium mass, and recovery factor. The schematic clarifies that k_loc is a lattice-normalized per-step removal fraction.

Supplementary Tables

The formal supplementary tables are listed below by title and purpose. File provenance and internal source paths are not part of the submitted Supplementary Tables; numerical multi-slice data are provided as Supplementary Data 1.

No.	Title	Brief description
Supplementary Table S1	Equation provenance and model components.	Summarizes the model equations, implementation-specific terms, and literature-supported components used in the revised manuscript.
Supplementary Table S2	CNN reconstruction plausibility benchmark.	Compares reported reference properties, the porosity-matched threshold baseline, and the CNN reconstruction for porosity, connectivity, hydraulic radius, and permeability-proxy metrics.
Supplementary Table S3	Hydraulic-radius and Knudsen sensitivity.	Lists how r_min, pressure, and pore-throat metrics affect Knudsen-number distributions and flow-regime fractions.
Supplementary Table S4	Thermodynamic convention and solubility checks.	Reports the representative gas-water partition quantities and correction factors used to check the revised convention.
Supplementary Table S5	LBM variable nomenclature and normalization.	Defines the symbols used for the LBM extraction calculation, including k_loc, k_diff, chi_u, Péclet number, and recovery factor.

Supplementary Data file summary

Supplementary Data 1 is provided as Supplementary_Data_multislice.xlsx. It contains the multi-slice extraction summary, time-series, metrics, reviewer-metric summary, and a source_manifest sheet within the same workbook.

Sheet	Description	Rows x columns
multislice_summary	Slice-level extraction summary metrics.	9 x 33
multislice_timeseries	Recovery, mass-balance, contact-area, and residual-helium time-series.	1305 x 29
multislice_metrics	Reviewer-facing metrics by slice.	9 x 7
reviewer_metrics_summary	Mean, standard deviation, and sample-size summary for key multi-slice metrics.	4 x 7
source_manifest	Workbook-level sheet inventory.	4 x 4
